# Leveraging spatial structure to design spatially-targeted malaria interventions at the community-scale

**DOI:** 10.1371/journal.pcbi.1014857

**Published:** 2026-10-15

**Authors:** Michelle V. Evans, Benjamin Roche, Vincent Herbreteau, Christophe Révillion, Thibault Catry, Matthew H. Bonds, Karen E. Finnegan, Ezra Mitsinjoniala, Felana A. Ihantamalala, Mauricianot Randriamihaja, Oméga Raobela, Andres Garchitorena

**Affiliations:** 1 MIVEGEC, Université de Montpellier, CNRS, IRD, Montpellier, France; 2 Pivot, Ranomafana, Ifanadiana, Madagascar; 3 Department of Global Health and Social Medicine, Blavatnik Institute, Harvard Medical School, Boston, Massachusetts, United States of America; 4 ESPACE-DEV, IRD, Université de Montpellier, Université de Perpignan, Université de Guyane, Université de La Réunion, Montpellier, France; 5 KHEOBS Laboratory, Institute of Technology of Cambodia, Phnom Penh, Cambodia; 6 Espace-Dev, Université de la Réunion, IRD, Université de Montpellier, Université de Perpignan, Université de Guyane, Saint-Pierre, La Réunion, France; 7 ED 168 CBS2, Université de Montpellier, Montpellier, France; 8 National Malaria Program, Ministry of Health, Antananarivo, Madagascar; The University of British Columbia, CANADA

## Abstract

Progress in malaria control has stagnated since the early 21st century in many countries, requiring new approaches such as the use of spatially-targeted interventions. However, evidence on the effectiveness of spatially-targeted interventions is mixed, depending on both the setting and intervention. In addition, the potential role of community health programs in implementing spatial targeting is underexplored, with models focusing on coarser spatial scales than those needed to guide programmatic activities. We developed a two-age-class, spatially-explicit model of malaria at the community-scale for a district in southeastern Madagascar, accounting for environmental heterogeneity and spatial structure, to explore spatially-targeted community health interventions. The model was fit to field-based case notifications and malaria prevalence data and then used to simulate three interventions: indoor residual spraying (IRS), supplemental coverage of long-lasting insecticide-treated nets (LLIN), and active case detection (ACD). We compared five scenarios, one with no spatial targeting and others targeting communities (i) nearest to clinics, (ii) furthest from clinics, (iii) with highest incidence, and (iv) that are spatially central. The non-targeted intervention was generally the most effective, but the least resource efficient. The second most effective intervention was based on spatial centrality, which reached a larger population while using fewer transportation resources than the non-targeted intervention. No combination of interventions was able to eliminate malaria in the district, although detecting symptomatic cases through ACD could avert 100% of severe malaria cases at high detection rates. These results highlight the potential for targeted malaria interventions, especially in low-income settings, that take into account spatial structure in the human population and transport networks to reduce malaria burdens using fewer resources than conventional district-wide interventions. [A French language abstract is available as Text A in S1 Appendix].

## Author summary

As progress against malaria slows, spatially-targeted interventions are one tool to control malaria in resource-limited contexts. Fine-scale targeting requires acting at the community scale, relying on community health workers to help implement programs. Using a mathematical model based on field-derived environmental, prevalence, and transport network data, we modeled malaria at the community-scale for a health district in Madagascar. We then used this model to compare different scenarios of spatially-targeted interventions using supplemental mosquito net distributions (LLIN), indoor residual spraying (IRS), and active case detection (ACD). IRS and ACD averted the most simple and severe malaria cases, respectively. Non-targeted interventions were the most effective, but required the most resources to implement; targeting based on the underlying transport and population structure averted a similar number of cases while using 2/3 of the resources. This work can help inform community malaria programs that wish to implement spatially-targeted interventions and is supported by a web application to explore different scenarios for community health programs in the district.

## Introduction

Following nearly two decades of steady reductions in the global malaria burden, progress began to stall in the late 2010s and, in some countries, has since reversed [1,2]. This has been due to a combination of factors. *Anopheles* mosquito vectors have become more resistant to the insecticide used in long-lasting insecticidal bednets (LLINs) and indoor residual spraying (IRS) campaigns [3], altering mosquito behavior to bite outside of homes, where exposure to insecticide is lessened [4]. The *Plasmodium falciparum* parasite itself is becoming more resistant to treatments; first, to monotherapies such as chloroquine, and now to the artemisinin-based combination therapy used globally to treat *P. falciparum* infections [5,6]. Global changes are shifting the endemic ranges of malaria, exposing new geographic regions and immune-naive populations to the disease [7,8]. Global spending on malaria control has been consistently below the WHO-recommended target by nearly 50% [9], with funding expected to become even more limited given predicted reductions in development funding [10,11]. New approaches are therefore needed to continue reducing malaria burdens globally and prevent losing recent progress while adapting to more limited resources.

One such approach is the use of spatially-targeted interventions, rather than “one-size-fits-all” national campaigns [12,13]. However, there is conflicting evidence on the effectiveness of spatially-targeted malaria interventions [14]. Prior work has found that targeted interventions are most effective in elimination settings, where malaria persists in isolated patches [15]. The metric chosen to guide a spatially-targeted intervention can also impact its effectiveness, with the proper metric dependent on the planned intervention [16]. In addition, the scale of the intervention and the geographic range of expected impact can influence the measured impacts [17–19]. Specifically, down-scaling targeted interventions to allow district health teams to design and guide the interventions within their district has been proposed as one method to successfully implement spatially-targeted campaigns while overcoming some of these weaknesses [17].

Within a health district, community health workers (CHWs) play a particularly important role in providing care. As local members of the community, CHWs provide basic health services to small catchments of the population [20], and often serve an integral role in disease control programs [21]. Due to their close proximity to patients, CHWs are well-positioned to conduct proactive care, such as active case detection (ACD) of malaria in patients with febrile illness [22,23], and encourage prophylaxis and preventive behaviors, such as intermittent preventive treatment during pregnancy [24,25]. Finally, because they cover localized catchments, they are a natural implementer of spatially-targeted campaigns to reduce overall costs [26], and have been used for spatially-explicit control of tuberculosis [27], maternal health program support [28], and pandemic response [29]. Notably, the success of these interventions depends on the strength of the community health programs, particularly the existence of reliable pharmaceutical stocks, financial support, and appropriate training and supervision for CHWs undertaking additional responsibilities [30,31]. While there is potential for CHWs to implement community-scale, spatially-targeted malaria campaigns, this requires alignment among data and interventions at appropriately fine spatial scales to guide operations. Few studies have empirically modeled malaria transmission dynamics in response to interventions at this spatial scale across an entire health district to inform local decision-making.

Here, we develop a spatially-explicit dynamic model of malaria that includes spatial structure via a transport network and heterogeneous environmental forcing to evaluate the impact of different control interventions at the community-scale for a high transmission, rural district in southeastern Madagascar. The model includes fine resolution spatial structure via a transport network based on a field-derived mapping exercise of all footpaths in the district and spatially-explicit climatic variables sourced from satellite imagery. We parameterize and fit the model to two independent datasets of malaria case and prevalence rates at the community-scale using Approximate Bayesian Computing (ABC). The fit model is used to compare the cases averted and the resources required by five different scenarios of spatial targeting of three common interventions: long-lasting insecticidal nets (LLIN), indoor residual spraying (IRS), and active case detection (ACD). The resulting estimates of averted cases are integrated into an online application that is available to local stakeholders that allows them to compare potential intervention scenarios within the district.

## Methods

### Study setting

Ifanadiana district is a rural health district located in the Vatovavy region of southeastern Madagascar (Fig A in S1 Appendix). The district’s population of approximately 200,000 is divided into 15 communes and 195 fokontany (the smallest administrative unit comprising several villages) [32]. One paved road runs east-west across the district, with an unpaved road connecting the northern and southern portions. The majority of the population lives in rural villages that are inaccessible by vehicle and are located further than one hour from the nearest health center [33]. Each commune contains at least one primary health center, and six communes contain an additional basic health center that provides more limited services, both of which are able to treat malaria cases of all-ages. Each fokontany has a community health site staffed by CHWs who are able to treat malaria cases in children under 5 years old and pregnant women [34]. While disruptions to the supply chain and staffing can impact the availability of malaria treatment and care from CHWs, the all-cause consultation rate at CHWs in Ifanadiana for children under 5 ranges from 1 -2 per capita consults per year [35], suggesting that they are serving the population in spite of these disruptions. The district includes a strong elevational gradient from an altitude of 1400m in the west to 100m in the east. The landscape is characterized by a forest-savanna matrix interspersed with agricultural land used for rice production. Although the district only encompasses an area of 3970 km ^2^, this diversity of ecoregions results in spatio-temporal heterogeneities in malaria burdens across the district [36]. A population-level survey conducted in 2021 found a district-level prevalence rate for children under 15 years old of 0.41, with village-level prevalence attaining rates as high as 0.8 [37].

Since 2015, Ifanadiana District has served as a model health district as part of a partnership between the Madagascar Ministry of Public Health and the non-governmental organization Pivot. This health system strengthening intervention includes improvements in system readiness and quality of care, a comprehensive monitoring and evaluation platform, and an actionable research program focused on generating evidence that can be used by local decision makers [38]. As part of the monitoring and evaluation platform, a district-representative, longitudinal cohort survey (Ifanadiana Health Outcomes and Prosperity longitudinal Evaluation, IHOPE) has been conducted every two years, collecting information on household demographics, disease incidence, and health system utilization [39]. Data from the 2021 wave of this survey was used to inform treatment rates, symptomatic rates, baseline intervention levels, and malaria prevalence rates in the model, described in detail below.

### Model definition

We developed a mathematical model of host and vector dynamics that modeled patch-specific (i.e., fokontany-level) transition rates on a daily timestep from January 1 2006 - December 31 2021. The model included host movement among patches defined by a modeled mobility matrix *m* derived from distance networks that represented the individual probability of moving from each source patch i to each destination patch j (*m*_*ij*_). Vector dynamics included environmental forcing through the inclusion of patch-specific daily temperature, rainfall, and rice field surface water dynamics. Finally, field-based baseline values of three interventions (treatment rates, long-lasting insecticide net (LLIN) coverage, and indoor residual spraying (IRS) coverage) were included in the model. Hypothetical scenarios then explored increasing these interventions’ coverage following different spatial targeting strategies.

Hosts were modeled via two age classes, children under-five and individuals five years of age and older (referred to as “adults”), corresponding to the age classes of reported case notification data. Each age class included 6 compartments representing different states of infection: susceptible (S_h_), exposed (E_h_), symptomatic infection (I_h_), subclinical infection (A_h_), severe (G_h_), and treated (P_h_) for each patch *i* (Fig 1, Eq. 1 – 6). The mosquito vector was modeled via 4 compartments: larval stage (L_V_), susceptible adults (S_V_), exposed adults (E_V_), and infected adults (I_V_) for each patch *i* (Fig 1, Eq. 7–10). Host and vector dynamics were linked via the entomological inoculation rate of the hosts (*EIR*, Eq. 11) and the force of infection on the mosquito vector (λ^i^_V_, Eq. 12), both of which were a function of the mobility model *m*_*ij*_. Host transition rates were simulated via a stochastic binomial probability and vector transition rates were simulated via a deterministic binomial probability. Parameters were sourced from the primary literature or estimated (Table A in S1 Appendix). Age-specific host parameters are denoted with a ‘*’.

**Fig 1:**
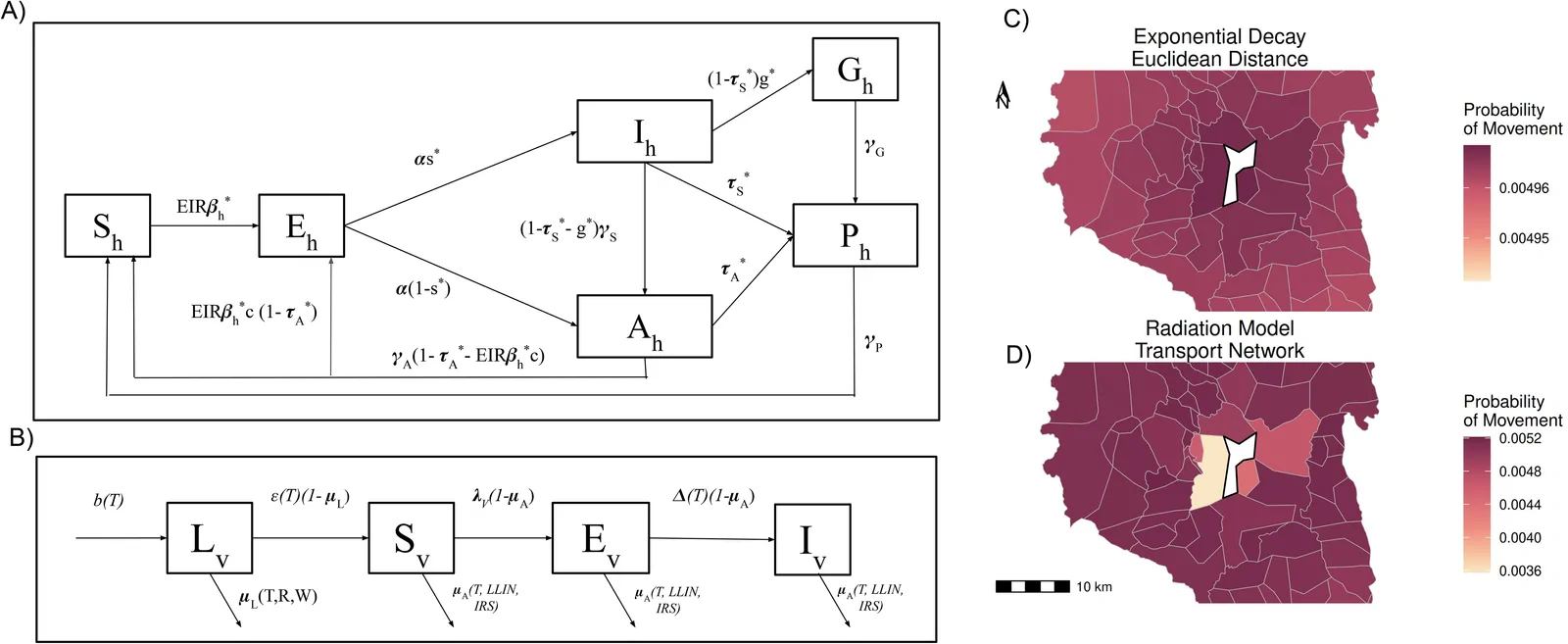
Diagram of compartmental model (A & B) and mobility models (C & D). A) Compartmental diagram of host compartments for one age-class. Parameters with a * superscript are age-specific. B) Compartmental diagram of vector compartments. Parameters represented as f() are a function of temperature T, rainfall R, or flooding W. C) Example metapopulation mobility model based on an exponential decay model on a euclidean distance network. Individuals are moving from the patch (fokontany) highlighted in white. D) Example metapopulation mobility model based on a radiation model on a transport network from OpenStreetMap. Individuals are moving from the patch (fokontany) highlighted in white. All parameters are defined in Table A in S1 Appendix. Boundaries in panels C & D represent fokontany boundaries (Source: UN OCHA, https://data.humdata.org/dataset/cod-ab-mdg).


$$\begin{array}{c} \frac{dS_{h}^{i*}}{dt}=A_{h}^{i*}\gamma_{a}\left(\text{1}-\tau_{A}^{*}-\beta_{h}^{*}EIR_{i}c\right)+P_{h}^{i*}\gamma_{p}-S_{h}^{i*}\beta_{h}^{*}EIR_{i} \end{array}$$ (1)

$$\begin{array}{c} \frac{dE_{h}^{i*}}{dt}=S_{h}^{i*}\beta_{h}^{*}EIR_{i}+A_{h}^{i*}\beta_{h}^{*}EIR_{i}c\left(\text{1}-\tau_{A}^{*}\right)-E_{h}^{i*}\alpha \end{array}$$ (2)

$$\begin{array}{c} \frac{dI_{h}^{i*}}{dt}=E_{h}^{i*}\alpha s^{*}-I_{h}^{i*}\tau_{s}^{*}-I_{h}^{i*}g^{*}\left(\text{1}-\tau_{s}^{*}\right)-I_{h}^{i*}\gamma_{s}\left(\text{1}-\tau_{s}^{*}-g^{*}\right) \end{array}$$ (3)

$$\begin{array}{c} \frac{dA_{h}^{i*}}{dt}=E_{h}^{i*}\alpha \left(\text{1}-s^{*}\right)+I_{h}^{i*}\gamma_{s}\left(\text{1}-\tau_{s}^{*}-g^{*}\right)-A_{h}^{i*}\tau_{A}^{*}-A_{h}^{i*}\beta_{h}^{*}EIR_{i}c\left(\text{1}-\tau_{A}^{*}\right)-A_{h}^{*}\gamma_{a}\left(\text{1}-\tau_{A}^{*}-\beta_{h}^{*}EIR_{i}c\right) \end{array}$$ (4)

$$\begin{array}{c} \frac{dG_{h}^{i*}}{dt}=I_{h}^{i*}g^{*}\left(\text{1}-\tau_{s}^{*}\right)-G_{h}^{i*}\gamma_{G} \end{array}$$ (5)

$$\begin{array}{c} \frac{dP_{h}^{i*}}{dt}=G_{h}^{i*}\gamma_{G}+I_{h}^{i*}\tau_{s}^{*}+A_{h}^{i*}\tau_{A}^{*}-P_{h}^{i*}\gamma_{P} \end{array}$$ (6)

$$\begin{array}{c} \frac{dL_{v}^{i}}{dt}=N_{v}^{i}b\left(T^{i}\right)-L_{v}^{i}\varepsilon \left(T^{i}\mathrm{\backslash rightleft}(\text{1}-\mu_{L}^{i}\right)-L_{v}^{i}\mu_{L}^{i} \end{array}$$ (7)

$$\begin{array}{c} \frac{dS_{v}^{i}}{dt}=L_{v}^{i}\varepsilon \left(T^{i}\mathrm{\backslash rightleft}(\text{1}-\mu_{L}^{i}\right)-S_{v}^{i}\lambda_{V}^{i}\left(\text{1}-\mu_{A}^{i}\right)-S_{v}^{i}\mu_{A}^{i} \end{array}$$ (8)

$$\begin{array}{c} \frac{dE_{v}^{i}}{dt}=S_{v}^{i}\lambda_{V}^{i}\left(\text{1}-\mu_{A}^{i}\right)-E_{v}^{i}\Delta \left(T^{i}\mathrm{\backslash rightleft}(\text{1}-\mu_{A}^{i}\right)-E_{v}^{i}\mu_{A}^{i} \end{array}$$ (9)

$$\begin{array}{c} \frac{dI_{v}^{i}}{dt}=E_{v}^{i}\Delta \left(T^{i}\mathrm{\backslash rightleft}(\text{1}-\mu_{A}^{i}\right)-I_{v}^{i}\mu_{A}^{i} \end{array}$$ (10)

$$\begin{array}{c} EIR^{i}=\sum_{j}\frac{I_{v}^{j}a^{j}m_{ij}}{\sum_{k}N_{h}^{k}m_{kj}} \end{array}$$ (11)

$$\begin{array}{c} \lambda_{V}^{i}=a^{i}\sum_{j}\frac{\left(I_{h_{j}}\beta_{V}+A_{h_{j}}\beta_{V_{A}}+P_{h_{j}}\beta_{V_{P}}\right)m_{ji}}{\sum_{k}N_{h_{k}}m_{kj}} \end{array}$$ (12)

Host individuals began susceptible, *S*_*h*,_ and transitioned to the exposed, *E*_*h*_, compartment as a function of the entomological inoculation rate (EIR) and an age-specific probability of transmission, β_h_^*^ (Eq. 1). The EIR was the average sum of the number of infectious mosquito bites received while resident and moving across all patches for an individual in patch i (Eq. 11). Exposed individuals became either symptomatic, *I*_*h*_, or subclincial, *A*_*h*_, according to the symptomatic rate, s^*^, and the latent period of malaria, *1/α* (Eq. 2–4). Symptomatic individuals developed severe malaria, *G*_*h*_*,* with probability g^*^, were treated with probability *τ*_s_* and entered *P*_*h*_*,* or remained untreated and eventually developed a subclinical infection at a rate of *ɣ*_*s*_ (Eq. 3). The treatment rates of symptomatic individuals were age-specific and based on reported treatment rates from the district of Ifanadiana [37] (Table A in S1 Appendix). Subclinical infections were either treated with probability *τ*_A_^*^, experienced a concurrent infection at a rate relative to a primary infection defined by the parameter *c,* or experienced waning immunity and became susceptible again following the patent period, 1/*ɣ*_*A*_ (Eg. 4). Subclinical individuals were treated at much lower rates than symptomatic individuals, assuming asymptomatic cases were identified during consultations for other febrile illnesses (Table A in S1 Appendix). Individuals with severe malaria (G_h_) entered the treated compartment after a severe clinical period, 1/*ɣ*_*G*_ (Eq. 5). Treated individuals became susceptible again at a rate of *ɣ*_*P*_ (Eq. 6). Population sizes were held constant throughout the simulations.

Mosquito larvae (L_V_) were born as a function of the total adult population *N*_*V*_ and temperature-dependent fecundity rates, *b(T),* assuming blood meal availability was not a limiting factor. They developed into susceptible adults (S_V_) via a temperature-dependent larval development rate, *ε(T),* and experienced temperature, rainfall, and density-dependent mortality, *μ*_*L*_ (Eq. 7, 13–15). Susceptible adults became exposed (E_V_) via the force of infection parameter *λ*_*V*_*,* which described the number of infectious bites that vectors were exposed to via both resident and moving hosts, accounting for hosts’ individual infectiousness as a function of age and infection status, β_v_^*^, and the patch-specific bite rate, *a*^*i*^ (Eq. 8). The patch-specific bite rate included a temperature-dependent bite rate and a protective effect of LLINs, which blocked a proportion of bites (Eq. 16). Exposed adults became infected (I_V_) following a temperature-dependent parasite development rate, *Δ(T),* (Eq. 9), and infected adults remained infected until death (Eq. 10). All adult mosquitoes experienced patch-specific mortality due to temperature, LLIN insecticide exposure, and IRS insecticide exposure (Eq. 17).

Both the *Anopheles* vector and *P. falciparum* pathogen exhibit temperature-dependent life history traits [36]. We therefore estimated daily, patch-level, temperature-dependent parameters for the vector bite rate, the parasite development rate, the vector development rate, the vector fecundity rate, the adult vector survival rate, and the larval vector survival rate. Temperature-dependent traits were estimated following either a Brier or quadratic function of daily temperature following Mordecai et al. [37]. The use of thermal performance curves allowed us to model temperature-dependent dynamics in multiple mosquito life stages, rather than a single reproductive number value, but did introduce further uncertainty and assumptions into the model. Specifically, we only included a mean daily temperature, ignoring diurnal fluctuations in temperature, which can be important towards thermal minima and maxima [38], and these functional curves were not made specifically for *Anopheles* species sourced from Madagascar, who may have different thermal adaptations than those used to estimate the thermal performance curves.

In addition to temperature-dependent larval mortality, larval mortality was caused by rainfall-induced flushing of larval habitat and density-dependence:


$$\begin{array}{c} \mu_{L}\left(T^{i},R^{i},W^{i}\right)=\text{1}-\left(\left(\text{1}-\mu_{L}\left(T^{i}\right)\right)*\left(\text{1}-\mu_{L}\left(R^{i}\right)\right)*\left(\text{1}-\mu_{L}\left(W^{i}\right)\right)\right) \end{array}$$ (13)

Larval mortality due to rainfall-caused flushing (*μ*_*L*_*(R)*) was an exponential function of the daily rainfall rate for that patch, *R*^*i*^ (Eq. 14) [38]:


$$\begin{array}{c} \mu_{L}\left(R^{i}\right)=\text{1}-\left(L_{f}+\left(\text{1}-L_{f}\mathrm{\backslash rightleft}(\left(\text{1}-K_{flush}\right)e^{-R^{i}/\tau_{flush}}+K_{flush}\right)\right) \end{array}$$ (14)

where *L*_*f*_ corresponded to the fractional larval growth state, *K*_*flush*_ was the maximum mortality rate due to flushing, R was the daily rainfall in mm, and 𝜏_flush_ was a scaling parameter to define the shape of the relationship between rainfall and larval mortality. All parameters were fixed following Tompkins and Ermert ([40], Table A in S1 Appendix).

Density-dependent mortality was a function of the available habitat, *W*, and a carrying capacity, K:


$$\begin{array}{c} \mu_{L}\left(W^{i}\right)=L_{v}^{i}/(W^{i}*K) \end{array}$$ (15)

The available habitat *W* was equal to the amount of flooded rice field (m^2^) in each patch because rice fields are a major larval habitat for *Anopheles* in the district [39]. The carrying capacity of mosquito larvae per m^2^, *K*, was estimated via model fitting.

Baseline coverage rates of LLIN and IRS were used to account for their impact on vector bite rates and adult mortality rates in each patch. The patch-specific bite rate included a temperature-dependent bite rate and a protective effect of LLINs that reduced the bite rate as a function of the time since LLIN distribution (t_D_) [39] and the LLIN coverage rate for that patch (C^*i*^_LLIN_), scaled by a mosquito endophagy rate of 0.3 [40]:


$$\begin{array}{c} a^{i}=a\left(T^{i}\right)*\left(\text{1}-\left({\text{0}.\text{9}\text{9}\text{9}\text{2}}^{t_{D}}*C_{LLIN}^{i}*\text{0}.\text{3}\right)\right) \end{array}$$ (16)

In addition to temperature-dependent mortality, adult mosquitoes experienced mortality due to LLIN insecticide exposure and IRS insecticide exposure:


$$\begin{array}{c} \mu_{A}^{i}=\text{1}-(\left(\text{1}-\mu_{A}\left(T^{i}\right)\right)*\left(\text{1}-\mu_{A}\left(LLIN^{i}\right)\right)*\left(\text{1}-\mu_{A}\left(IRS^{i}\right)\right) \end{array}$$ (17)

LLIN-induced mortality was a function of LLIN coverage described above in Eq. 16, mosquito bite rates and the bioefficiacy decay rate of permethrin estimated from field-data from Madagascar [39]:


$$\begin{array}{c} \mu_{A}\left(LLIN^{i}\right)=a\left(T^{i}\right)*{\text{0}.\text{9}\text{9}\text{9}\text{2}}^{t_{D}}*C_{LLIN}^{i}*\text{0}.\text{3}*\frac{\text{9}\text{0}.\text{6}\text{5}\text{0}*e^{-\text{0}.\text{0}\text{0}\text{6}\text{4}\text{0}\text{9}\text{3}\text{3}\text{3}{t_{D}}^{i}}}{\text{1}\text{0}\text{0}} \end{array}$$ (18)

IRS-induced mortality was a function of IRS coverage (C_IRS_) and the waning efficacy of bendiocarbs following Sherrard-Smith et al. [41]:


$$\begin{array}{c} \mu_{A}\left(IRS^{i}\right)=\frac{\text{1}}{\text{1}+e^{\text{1}.\text{0}\text{9}\text{5}-\text{0}.\text{0}\text{2}\text{5}{t_{D}}^{i}}}*C_{IRS}^{i} \end{array}$$ (19)

#### Spatial structure.

Spatial structure was included via modeled daily movements between patches estimated from field-based distance networks. At each timestep, hosts and vectors could infect each other in the patches hosts traveled to, defined via the mobility matrix *m* (Fig 1C and 1D). However, they remained “residents” of their origin patch and did not contribute to host compartments in other patches. The mobility matrix was used to estimate the EIR of human hosts and the force of infection on mosquito vectors, *λ*_*V,*_ taking into account host movement patterns (Eq. 7 and 12). These values represented the sum of the product of infectious contacts and transmission possibilities across all destination patches *j* for each source patch *i*.

Mobility matrices *m* were created from two distance datasets that described the pairwise distance between each patch: a naive dataset representing the euclidean distance and a transport-network based distance dataset. We applied three mobility models to each dataset of pairwise distances: an exponential decay model, a gravity model that took into account the patch populations, and a radiation model that included patterns in population values of neighboring patches. For the exponential decay model, the probability of movement between patches *i* and *j* decreased with increasing distance between the patches, *d*_*ij*_:


$$\begin{array}{c} m_{ij,exp}=\frac{\text{1}}{\left(\text{1}+e^{\left(\beta_{\text{0}}+\beta_{\text{1}}d_{ij}\right)}\right)} \end{array}$$ (20)

For the gravity model, the probability of movement also decreased with increasing distance, but this effect was mediated by population size, with patches with larger populations attracting more individuals from other patches:


$$\begin{array}{c} m_{ij,GRAV}=\frac{\text{1}}{\left(\text{1}+e^{\beta_{\text{0}}+\beta_{\text{1}}\left(d_{ij}/{\left(p_{i}p_{j}\right)}^{\beta_{\text{2}}}\right)}\right)} \end{array}$$ (21)

For the radiation model, the probability of moving to patch *j* was dependent on the distance between patches *d*_*ij*_ and the population of patches *p* via the radiation model rad’. Population values of neighboring patches were included via the parameter s_ij_, which represents the total population within a distance d_ij_ of patch i, excluding patches i and j [42,43]:


$$\begin{array}{c} m_{ij,RAD}=\frac{\text{1}}{\left(\text{1}+e^{\beta_{\text{0}}+\beta_{\text{1}}d_{ij}+\beta_{\text{2}}rad^{\prime }}\right)} \end{array}$$ (22)

$$\begin{array}{c} ad^{\prime }=\frac{p_{i}p_{j}}{\left(p_{i}+s_{ij}\mathrm{\backslash rightleft}(p_{i}+p_{j}+s_{ij}\right)} \end{array}$$ (23)

These models were applied to each distance dataset separately. For all models, the probability of remaining in patch i (m_i,j=i_) was a fit parameter *p*_*m*_, replacing the diagonal of the mobility model matrix. Each mobility model was then scaled so that the total sum of movement probabilities for each patch i ($\sum_{j}m_{ij}$) equaled 1. The value m_ij_ was then used as the daily probability of moving from patch i to j for each individual in patch i. Combined with a model of no movement between patches and a “well-mixed” model where there was no limit on inter-patch movement, this resulted in eight total mobility models that were assessed. All model parameters 𝛽 and *p*_*m*_ were estimated via model fitting.

### Data collection

#### Malaria case notification and prevalence data.

The number of monthly malaria cases per patch were collected from January 2016 to June 2021 from consultation registries of all primary health centers in the district (Fig 2). Handwritten registries were digitized and each case was geolocated to the precision of a fokontany (i.e., patch). The number of cases for children under 5 years old, children aged 5–14, and adults aged 15 years and above were summed, including both simple and severe cases. Following Evans et al. [44], these raw data were adjusted for underascertainment often found in passive surveillance data due to spatial biases in healthcare access using the ZERO-G method [37]. The method was applied separately to each of the three age classes above to estimate a monthly case rate. Case rates were transformed into case counts by scaling the population of each age class and were ultimately aggregated into the two age classes used in the model: children under 5 years old and persons aged 5 or older, referred to as adults. The ZERO-G adjustment was unique to each month and fokontany and resulted in case rates on average 5.72 times higher than the raw case rates (IQR: 2 – 6.6), dependent on the level of health care access (Fig B in S1 Appendix).

**Fig 2:**
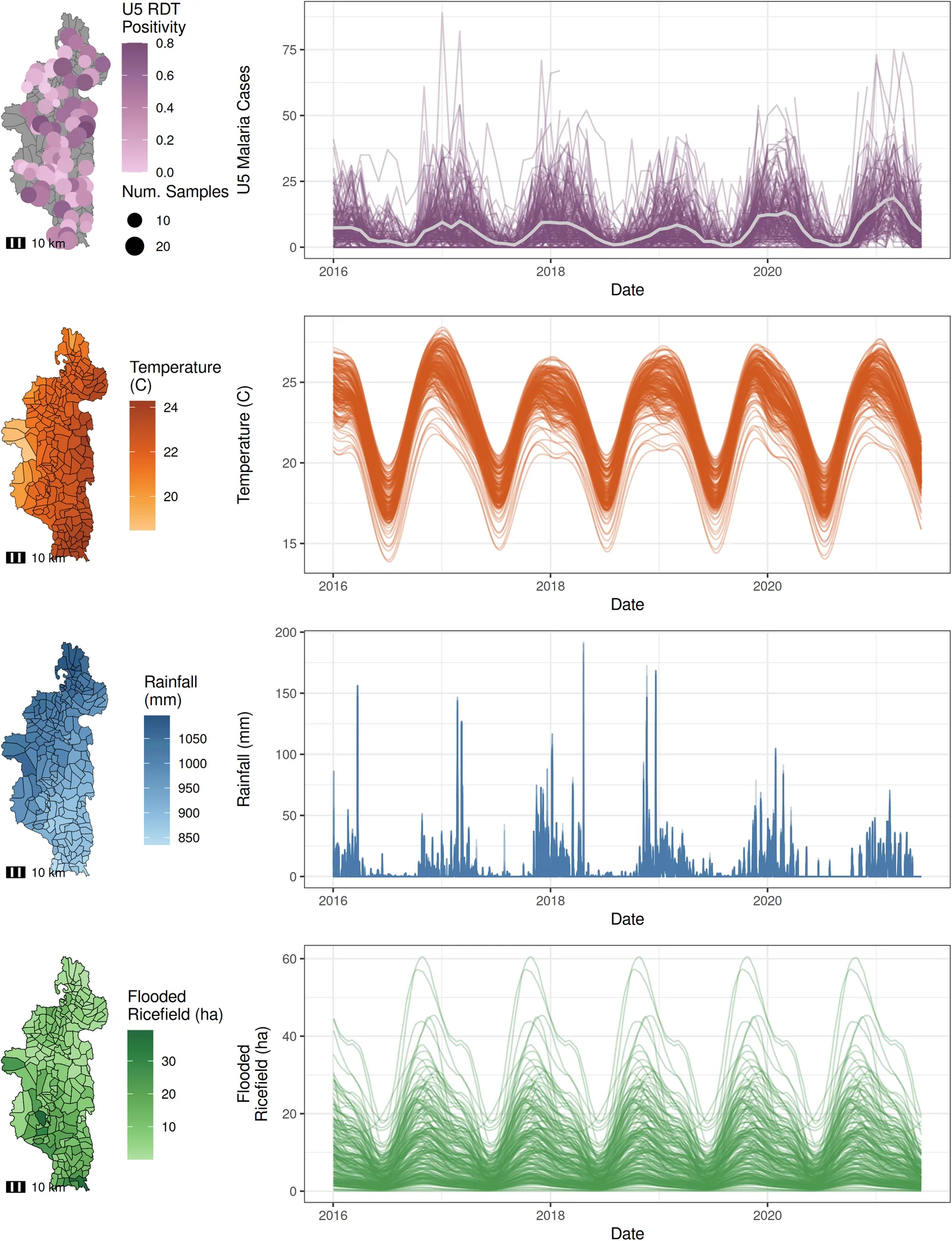
Seasonal and spatial patterns exist in malaria positivity and case rates and climatic variables. Row 1: Map of malaria positivity rates and time series of cases in children under five years old. Each individual fokontany is plotted in the time series, with the district-level mean in gray. Row 2: Average annual temperature by fokontany from 2016-2021 and daily temperature in the time series. Row 3: Average cumulative rainfall (mm) by fokontany from 2016-2021 and daily total rainfall amounts. Row 4: Average annual area of flooded rice fields (ha) by fokontany from 2016-2021 and daily flooding of rice fields. Boundaries in maps represent fokontany boundaries (Source: UN OCHA, https://data.humdata.org/dataset/cod-ab-mdg).

Malaria prevalence for children under 5 years old was collected via the IHOPE survey conducted in April-June 2021 in 1600 households in 109 fokontany throughout the district (Fig 2). Briefly, children under 15 years old who consented to the study were tested for active malaria infection using SD One Step Malaria HRP-II(P.f) and pLDH(Pan) Antigen Rapid Tests. Those who tested positive were provided with a standard treatment of artesunate amodiaquine and paracetamol, with duration and dosage in accordance with national guidelines. For this study, we limited our prevalence estimates to children under 5 years old, so that it paired with the child age class in the dynamic model, resulting in 969 samples. Patch-level prevalence was defined as the mean prevalence of all individuals in clusters within that patch.

#### Distance network data.

Mobility models were run on two distance networks: a naive network and a transport network. The naive network represented the pairwise euclidean distance between the centroid of each fokontany. The distance based on a transport network was estimated using a comprehensive dataset of over 25,000 km of tracks and footpaths, 5,000 residential zones, and 100,000 buildings available in OpenStreetMap [33]. The shortest pairwise distance between the largest village in each fokontany on the network was estimated using the Open-Source Routing Machine service [45]. Both approaches resulted in a matrix of pairwise distances between all patches *i* and *j* (*d*_*ij*_).

#### Environmental data.

As a mosquito-borne disease, malaria dynamics are highly dependent on the surrounding environment, particularly climate and the availability of larval habitat [46–48]. Data on temperature, rainfall, and ricefield flooding dynamics were used as environmental forcing to model vector dynamics, as detailed above (Fig 2). Temperature data was sourced from the MODIS Aqua Daily Land Surface Temperature dataset following [44]. This method uses a gap-filling algorithm to create daily estimates of land temperature at a 1 km resolution [49], which was then extracted to the patch-level. This resulted in a daily time series of temperature for each patch from January 1 2006 through December 31 2021. Daily rainfall data was derived from the Africa Rainfall Climatology 2 Model [50] and the spatial mean extracted at the patch-level, resulting in a daily time series of rainfall for each patch from January 1 2006 through December 31 2021. Rice field flooding dynamics were described by estimating the daily value of the amount of flooded rice fields in m^2^ per patch using a field-tested, radar-based classification algorithm developed for this region of Madagascar [51]. This data was collected from the Sentinel-1 satellite constellation for January 1 2016 through Dec 21 2021. Because Sentinel-1 timeseries data was not available for dates before 2016, the average for that day of the year was used for January 1 2006 - December 31 2015.

### Parameter estimation

Transition rates and mobility model parameters were estimated for each mobility model through Approximate Bayesian Computing based on Sequential Monte Carlo (ABC – SMC, [52]). ABC-SMC allows the estimation of parameters where the log-likelihood is intractable or expensive to compute, or when multiple datasets or customized error functions are to be considered [53,54]. In our case, it enabled us to fit the model to malaria prevalence and incidence data simultaneously and include true environmental forcing data which prohibits exact estimation of the log-likelihood. Applying ABC-SMC, we fit the dynamic models to monthly case notification data for each age group from Jan 2016 - June 2021 and malaria prevalence rates in children under-5 from May 2021, for a total of five summary statistics: the root mean square error between simulated and observed monthly adult cases by patch; the root mean square error between simulated and observed monthly child cases by patch; the proportion of months within 20% of the observed case numbers occurring during the malaria peak (November - May); the mean absolute error in simulated and observed prevalence in children; and the correlation of the average annual simulated and observed case count for each patch. The district-level mean of each statistic was calculated via a weighted mean, where the weight of each patch was proportional to the number of months of complete, non-zero observations in the case notification data.

The ABC-SMC process was run for ten generations or until the particle acceptance rate dropped below 1% for each mobility model. The initial generation began with manually selected thresholds for the summary statistics, with sequential generations adopting the median of the prior generation’s accepted particles’ performance as the threshold. Potential particles were generated from a covariance matrix of the previous generation of accepted particles containing the 25 nearest neighbors, following Minter and Retkute [54]. Each generation was simulated until 1000 particles were accepted. Posteriors were then generated from the last generation’s distribution of 1000 particles, weighted by a multivariate prior drawn based on the covariance matrix of the 25 nearest neighbors for that particle.

### Simulating spatially targeted interventions

#### Spatial targeting scenarios.

We explored different scenarios of spatial targeting of each intervention, whereby coverage rates of an intervention were increased from baseline levels in certain fokontany (i.e., patches) based on their spatial characteristics or historical disease incidence. These interventions represented supplemental interventions on top of currently existing coverage rates in the district and did not reduce an intervention’s coverage in fokontany relative to baseline. In addition to a non-targeted scenario, four scenarios of spatial targeting were considered in this study. All targeted scenarios targeted 50% of the fokontany with approximately twice the coverage rate of a non-targeted intervention, so that the overall district-wide intervention effort remained similar between non-targeted and targeted interventions. The first scenario targeted those fokontany nearest to health clinics on the road network, representing the least isolated and generally more populated fokontany (*Nearest to Clinic*). The second scenario targeted those fokontany further from health clinics on the road network, representing more isolated fokontany with lower access to health care (*Furthest from Clinic*). The third scenario targeted the fokontany with the highest malaria incidence rates in 2016, representing fokontany with potentially higher malaria exposure (*Highest Historical Incidence*). The fourth scenario targeted the most central fokontany on the radiation mobility network, representing the most “connected” fokontany (*Spatial Centrality)*. This was defined as the average probability of movement to each patch from other patches. The fifth scenario was non-targeted, and simply applied the intervention to all fokontany equally at the lower coverage rate (*Non-Targeted)*.

#### Intervention scenarios.

The best fit mobility model was then used to simulate the impact of spatially targeted and non-targeted interventions in the district. Three interventions were considered: active case detection (ACD), supplemental long-lasting insecticide net (LLIN) distributions, and indoor residual spraying (IRS) campaigns. All three interventions are part of the Strategic Plan of the Madagascar National Malaria Control Programme (*Programme Nationale de Luttre Contre le Paludisme, PNLP)* for the period of 2023–2027. Interventions were simulated beginning on January 1 2016.

ACD interventions were simulated to increase treatment rates for symptomatic individuals across both age groups, τ_s_^*^. Base treatment rates were estimated for children under-5 years old and adults from the 2021 IHOPE survey, with rates of 0.5658 and 0.3319, respectively. The non-targeted intervention increased these rates by 50% to 0.8486 and 0.4979 for children and adults, respectively, across all patches in the district. The targeted interventions increased both treatment rates to 1 in the targeted patches, corresponding to the PNLP’s malaria treatment rate goal for 2026. To account for gradual increases in treatment rates, all interventions increased the treatment rate linearly from the baseline treatment rate to the goal treatment rate over the six month period prior to the start of the intervention.

Supplemental LLIN interventions increased the existing baseline coverage rate of 50% used in the initial model based on IHOPE 2021 data. Supplemental LLIN distributions were set to occur on August 1st on a three year cycle beginning in 2003, following PNLP policy. The non-targeted strategy increased existing coverage rates by 50% to equal 75% coverage in all patches and spatially-targeted interventions increased coverage rates by 100% to equal 100% coverage in targeted patches. These values were chosen to keep the district-wide effort similar between targeted and non-targeted interventions. LLIN distributions impacted adult mosquito bite rates and mortality rates in the patch benefiting from that intervention following Equations 16 and 18. Hosts moving out of their residential patch were therefore exposed to infectious bite rates based on the LLIN coverage in the patch they were visiting.

IRS campaigns were set to occur on November 1 of each year on an annual basis beginning in 2016. Baseline coverage rates were 0, as no IRS campaigns are currently done in Ifanadiana. The non-targeted strategy increased IRS coverage to 0.30 in all patches, following PNLP targets. Spatially-targeted interventions increased IRS coverage rates to 0.60 in targeted patches. This difference in coverage rates was done to impose similar district-wide efforts for both targeted and non-targeted interventions. IRS campaigns impacted adult mosquito mortality rates following Eq. 19.

#### Sensitivity analyses.

Spatially-targeted malaria interventions can have indirect effects on disease in neighboring fokontany via “community-level” effects [55]. The direction of these effects are dependent on the relative difference in disease burden between patches and their connectivity [56]. We therefore conducted a sensitivity analysis to explore the relative effectiveness of different targeting strategies under different population mobility rates. Using the best fit mobility model, we fixed the probability of remaining in the source patch (*p*_*m*_) while using all other mobility parameters as estimated. This resulted in three mobility levels: low (25% move), medium (50% move) and high (75% move). We then simulated the same combination of interventions and targeting strategies on each of these three scenarios and compared the average number of annually averted cases across 19 iterations.

Because it benefits from on-going health system strengthening initiative, Ifanadiana’s baseline malaria testing and treatment rates may not be representative of national trends. Indeed, in the neighboring district of Farafangana, malaria treatment rates range between 19.6 – 23.4% [57], compared to rates of 33.2 – 56.6% in Ifanadiana. We therefore conducted a sensitivity analysis to explore the impact of ACD interventions at a lower baseline treatment rate, while keeping all other parameters as estimated. Following Sayre et al. [57], we used a 20% baseline treatment rate for both children and adults and explored both low and high coverage rates of 40% and 80% respectively. For targeted interventions, ACD coverage responded to 80% and 100% for low and high coverage, respectively. We then simulated these interventions for all targeting strategies and both coverage rates and compared the average number of annually averted cases across 19 iterations.

#### Assessing and visualizing intervention scenarios for decision-making.

Each combination of intervention and spatial targeting scenarios (15 total) was simulated 49 times using the best fit parameters from the model fitting exercise. We then estimated the median number of simple and severe malaria cases averted per age-class relative to the baseline scenario with no additional interventions to assess the impact of each intervention scenario. We also assessed combining two or all three interventions for each of the five targeting scenarios at low and high levels of coverage. Low coverage was equal to 1.5x base levels, with a maximum of 100% coverage. High coverage was equal to 2x base levels, with a maximum of 100% coverage. Coverage was increased in targeted fokontany, with other fokontany retaining base coverage levels.

Coverage rates were set lower for non-targeted scenarios to approximate equal intervention efforts at a district-wide level for targeted and non-targeted interventions based on the number of patches targeted. However, the total resources required by an intervention can also depend on the distance needed to travel to implement the intervention and the number of households impacted. We therefore estimated the total distance from targeted fokontany to the nearest health center, the total distance from targeted fokontany to the district hospital, the total number of households targeted by IRS and supplemental LLIN, and the total number of individuals benefiting from ACD for each targeting scenario to better estimate the overall resources required for each scenario.

As part of this model health district’s program to conduct actionable research, we developed an accompanying web application to allow local health actors to explore a wider range of intervention scenarios. This includes the ability to choose single or multiple interventions, the type of spatial targeting, and the coverage rate of the intervention. Users can then explore time series and maps of the number of simple or severe malaria cases resulting from the simulated intervention scenario at fokontany and district-levels. This application was developed using R Shiny and is available at https://smaller.pivot-dashboard.org/ on the ‘Interventions’ tab.

## Results

### Spatial variation in parameters due to environmental forcing

There was a range of spatial heterogeneity in the environmental forcing parameters. When considering daily variation (e.g., spatial differences at the same time point) across the district, temperature varied by 5.15 - 6.95 C, rainfall by 0 - 281.3 mm, and flooded ricefields by 18.6 - 60.5 ha, depending on the season (Fig 2). There was large daily variation in temperature-dependent vector parameters throughout the study period (Table B and Fig C in S1 Appendix). Pathogen and larval development rates were the most varied temperature-dependent rates across space with average coefficients of variation across the district of 0.156 (0.0813 *sd)* and 0.155 (0.0817 *sd)*, respectively. This was equivalent to certain patches having development rates over twice as high as the average development rate during the same timestep. In contrast, adult survival was least sensitive to spatial heterogeneity in temperature, with rates never differing by more than 0.06 across the district (Table B in S1 Appendix). Larval mortality due to rice-field carrying capacity also exhibited distinct spatial heterogeneity, with an average coefficient of variation above 0.83 (Table B in S1 Appendix). The median range in maximum larval carrying capacity across the district was 9.6 million larvae per fokontany, with certain fokontany experiencing carrying capacities over five times higher than average during the same time step. Rainfall induced larval mortality rates, which were based on coarser resolution rainfall data and included a non-linear functional response, were notably less varied across space (Table B and Fig C in S1 Appendix). On average, there was no difference in rainfall-induced mortality rates across the district, with only 5.0% of timesteps with differences greater than 0.1.

### Model performance

The majority of mobility models reached convergence with an acceptance rate lower than 1% within 9 generations of the adaptive threshold, with the exception of the Full Mobility model which reached low acceptance rate values after 7 generations (Table 1). The No Mobility model had an extremely low effective sample size (ESS), representing the effective number of independent samples in the final particle set. Higher ESS is associated with higher precision and convergence among the SMC sampling algorithm for a model. Among the more complex mobility models, most had ESS over 100, with the exception of the exponential decay model on the euclidean distance network, and both the gravity and exponential decay models had over 200 ESS on the transport network (Table 1). We evaluated performance on 499 simulations drawn from the posteriors for each mobility model. The No Mobility and Full Mobility models performed the poorest across all five summary statistics, with the exception of RMSE - Adult Cases, where the Full Mobility model performed similarly to the distance-based models (Fig 3). Among the six distance-based models, there were no consistent trends in performance across the five summary statistics and all six performed similarly (Fig 3). Spatial patterns in mobility were similar across the Exponential Decay models and the Gravity model on the Euclidean distance network, quickly decaying with distance to the source patch, while the remaining mobility models exhibited more spatial clustering in mobility patterns (Fig D in S1 Appendix). To identify the best fit model, we ranked the models within each summary statistic according to their median summary score and estimated the mean ranking by model. The radiation model using network distance from transport data (Radiation - Transport model) was identified as the best fit and used to compare interventions.

**Table 1:** Model performance indicators from ABC-SMC fitting on all eight mobility models. A well-fit model has run for more generations, has a lower acceptance rate, and a higher effective sample size.

Mobility Model	Generation	Acceptance Rate	Effective Sample Size
No Mobility	10	0.0015	13.05
Full Mobility	7	0.184	91.59
Exp. Decay - Euclidean	9	0.176	26.91
Gravity - Euclidean	9	0.011	100.87
Radiation - Euclidean	9	0.016	173.33
Exp. Decay - Transport	9	0.017	223.27
Gravity - Transport	9	0.016	201.02
Radiation - Transport	9	0.015	120.68

**Fig 3:**
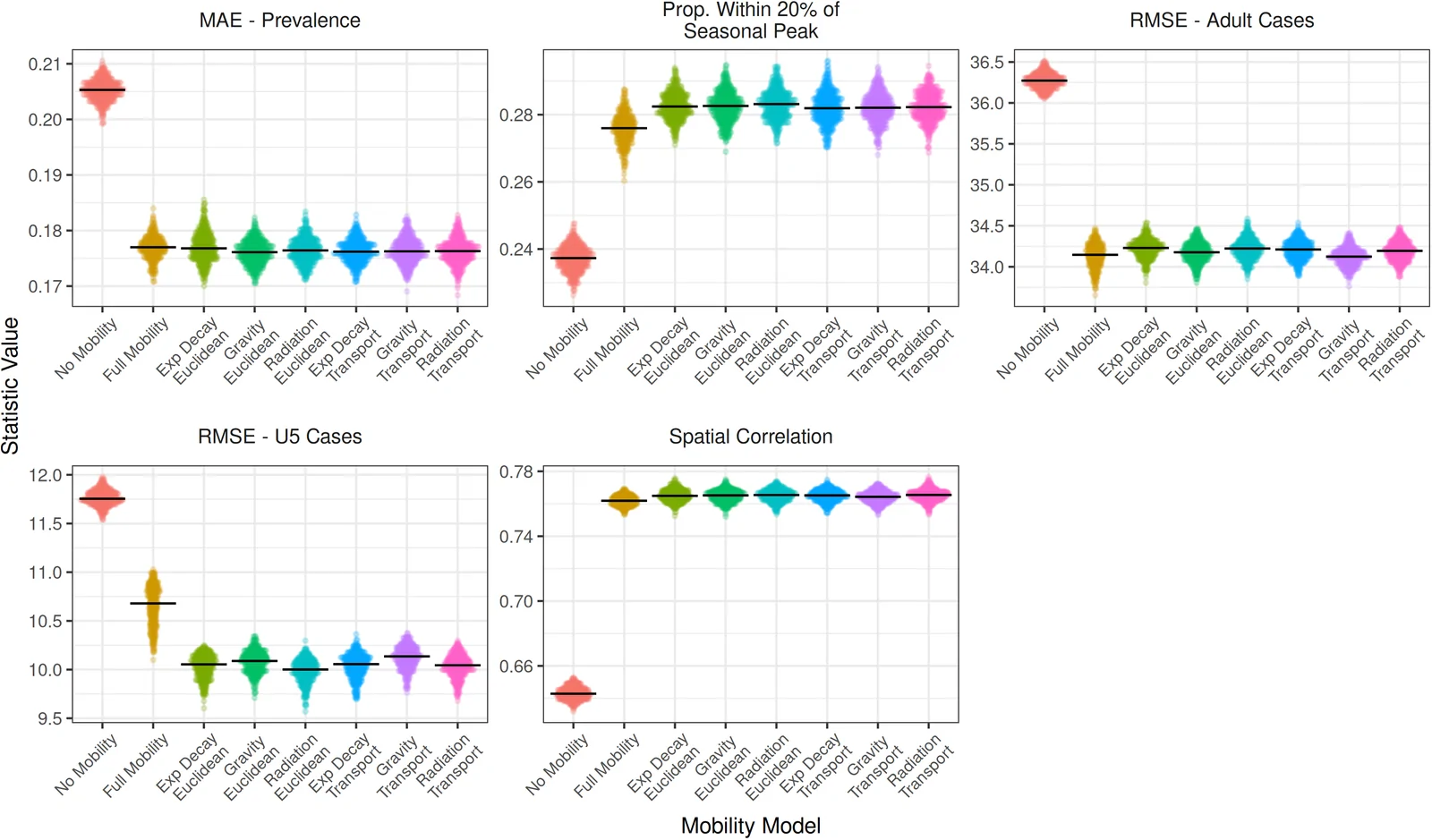
Mobility models that included patch-specific movement performed similarly on summary statistics. Results of 499 individual simulations from the posterior for each mobility model (points) with the median value represented by the bold line. Simulations with performance scores outside of the initial threshold values were removed to aid in visualization.

### Intervention scenarios

When interventions were non-targeted, IRS interventions were the most effective against simple malaria cases, averting on average 38.1% of cases in adults and 35.1% of cases in children (Table 2). LLIN distributions and ACD were considerably less effective, reducing cases between 7.37 – 8.17% and 0.04 – 0.008%, respectively (Table 2). However, when considering severe malaria cases, ACD was the most effective intervention, averting on average 42.9% of adult cases and 74.5% of childhood cases (Table 2). The other interventions performed similarly for simple and severe cases, although there was more uncertainty around severe cases averted given the lower overall numbers (Table 2). The number of severe childhood malaria cases was relatively low at the patch level, with on average one case per year. Because of this, the stochastic binomial transition rates introduced high amounts of demographic stochasticity across iterations.

**Table 2:** Equally distributed scenarios generally avert the most cases for all three types of interventions, particularly for children under 5. Average annual cases averted by targeting scenario and intervention for simple and severe malaria cases for children under 5 years old and adults and children 5 years and older. The range of annual averted cases between 2016-2021 are shown in parentheses. ACD: active case detection; IRS: indoor residual spraying; LLIN: long-lasting insecticidal nets.

		Simple Malaria Cases						Severe Malaria Cases					
		IRS		LLIN		ACD		IRS		LLIN		ACD	
	Target Scenario	Cases Averted	% Averted	Cases Averted	% Averted	Cases Averted	% Averted	Cases Averted	% Averted	Cases Averted	% Averted	Cases Averted	% Averted
**Children Under 5**	*Non-Targeted*	8080 (7881-8411)	35.13% (33.89 – 36.43%)	1686 (1242-2420)	7.37% (5.17 – 10.69%)	193 (151-214)	0.84% (0.63 – 0.96%)	68 (62-74)	36.23% (33.68 – 38.74%)	15 (11-19)	7.89% (5.56 – 10.92%)	141 (130-147)	74.48% (73.06 – 75.92%)
	*Nearest to Clinic*	4458 (4341-4550)	19.38% (18.50 – 19.86%)	1599 (1255-2232)	6.98% (5.23 – 9.86%)	148 (125-189)	0.64% (0.52 – 0.83%)	40 (37-44)	21.03% (19.69 – 22.22%)	12 (7-19)	6.59% (3.66 – 10.92%)	103 (92-113)	54.57% (52.87 – 57.07%)
	*Furthest From Clinic*	4367 (4249-4452)	18.99% (18.31 – 19.31%)	1546 (1168-2185)	6.75% (5.06 – 9.65%)	133 (109-187)	0.58% (0.47 – 0.84%)	34 (32-36)	18.27% (17.17 – 18.85%)	10 (8-13)	5.61% (4.04 – 7.47%)	87 (79-93)	46.15% (44.04 – 48.17%)
	*High Historical Incidence*	4434 (4285-4538)	19.28% (18.37 – 19.88%)	1582 (1246-2202)	6.91% (5.20 – 9.73%)	137 (109-153)	0.60% (0.45 – 0.68%)	38 (34-44)	20.11% (18.65 – 23.04%)	14 (11-17)	7.35% (5.56 – 9.77%)	97 (88-101)	51.31% (50.57 – 52.88%)
	*Spatial Centrality*	4693 (4589-4795)	20.40% (19.62 – 20.77%)	1679 (1294-2351)	7.33% (5.44 – 10.39%)	154 (82-210)	0.67% (0.36 – 0.93%)	40 (38-43)	21.32% (20.21 – 22.51%)	17 (10-22)	8.96% (5.24 – 12.64%)	112 (103-117)	59.27% (57.07 – 60.62%)

**Adults and Children 5+**	*Non-Targeted*	18979 (17898-20073)	38.08% (35.93 – 39.89%)	4038 (3310-5818)	8.17% (6.34 – 12.30%)	229 (221-234)	0.46% (0.44 – 0.49%)	34 (33-35)	40.34% (37.93 – 43.42%)	6 (2-11)	7.88% (2.30 – 12.50%)	36 (32-41)	42.88% (41.38 – 46.59%)
	*Nearest to Clinic*	10876 (10529-11365)	21.83% (20.58 – 22.58%)	3883 (3266-5454)	7.85% (6.38 – 11.53%)	302 (256-346)	0.61% (0.52 – 0.68%)	20 (17-23)	23.50% (19.54 – 28.05%)	6 (1-10)	7.33% (1.15 – 13.16%)	46 (41-50)	54.90% (53.66 – 56.82%)
	*Furthest From Clinic*	10619 (10263-11092)	21.31% (20.19 – 22.04%)	3794 (3165-5332)	7.67% (6.27 – 11.27%)	241 (205-305)	0.48% (0.39 – 0.61%)	20 (17-23)	23.97% (22.37 – 26.14%)	6 (4-11)	7.14% (4.60 – 12.50%)	39 (35-44)	46.84% (42.68 – 50.00%)
	*High Historical Incidence*	10800 (10393-11293)	21.67% (20.61 – 22.44%)	3832 (3256-5371)	7.75% (6.32 – 11.35%)	266 (230-345)	0.54% (0.46 – 0.70%)	18 (17-19)	21.37% (19.32 – 22.37%)	6 (1-10)	6.93% (1.22 – 11.36%)	42 (36-46)	50.34% (47.37 – 52.87%)
	*Spatial Centrality*	11412 (11006-11879)	22.90% (21.67 – 23.60%)	4066 (3488-5673)	8.22% (6.68 – 11.99%)	338 (300-362)	0.68% (0.63 – 0.72%)	20 (17-22)	23.51% (20.69 – 27.63%)	8 (5-13)	9.92% (5.75 – 14.77%)	50 (44-56)	60.26% (57.89 – 63.64%)

In general, targeting scenarios based on the distance to the health clinic or historical incidence performed worse than the Non-Targeted scenario for all interventions and case types (Fig 4 and Table 2). However, the Spatial Centrality targeting scenario performed similar to the Non-Targeted scenario for the LLIN intervention, and was the second best targeting scenario for IRS (Fig 4 and Table 2). This effect was diminished for severe cases, where the Non-Targeted scenario consistently performed best, except for adult cases using an ACD intervention, where the Non-Targeted scenario averted the fewest number of cases (Table 2). When considering differing mobility rates, we found that intervention effectiveness increased with increasing mobility rates for all targeting scenarios (Fig F in S1 Appendix). The sensitivity analysis exploring the impact of an ACD intervention at lower baseline treatment rates found similar results as the main analysis. ACD averted between 0.949-1.64% of simple cases, with little difference across low and high coverage. The number of severe cases averted was higher with higher coverage, ranging between 46.6-47.7% at low coverage and 60.1-60.7% at high coverage levels (Table C in S1 Appendix).

**Fig 4:**
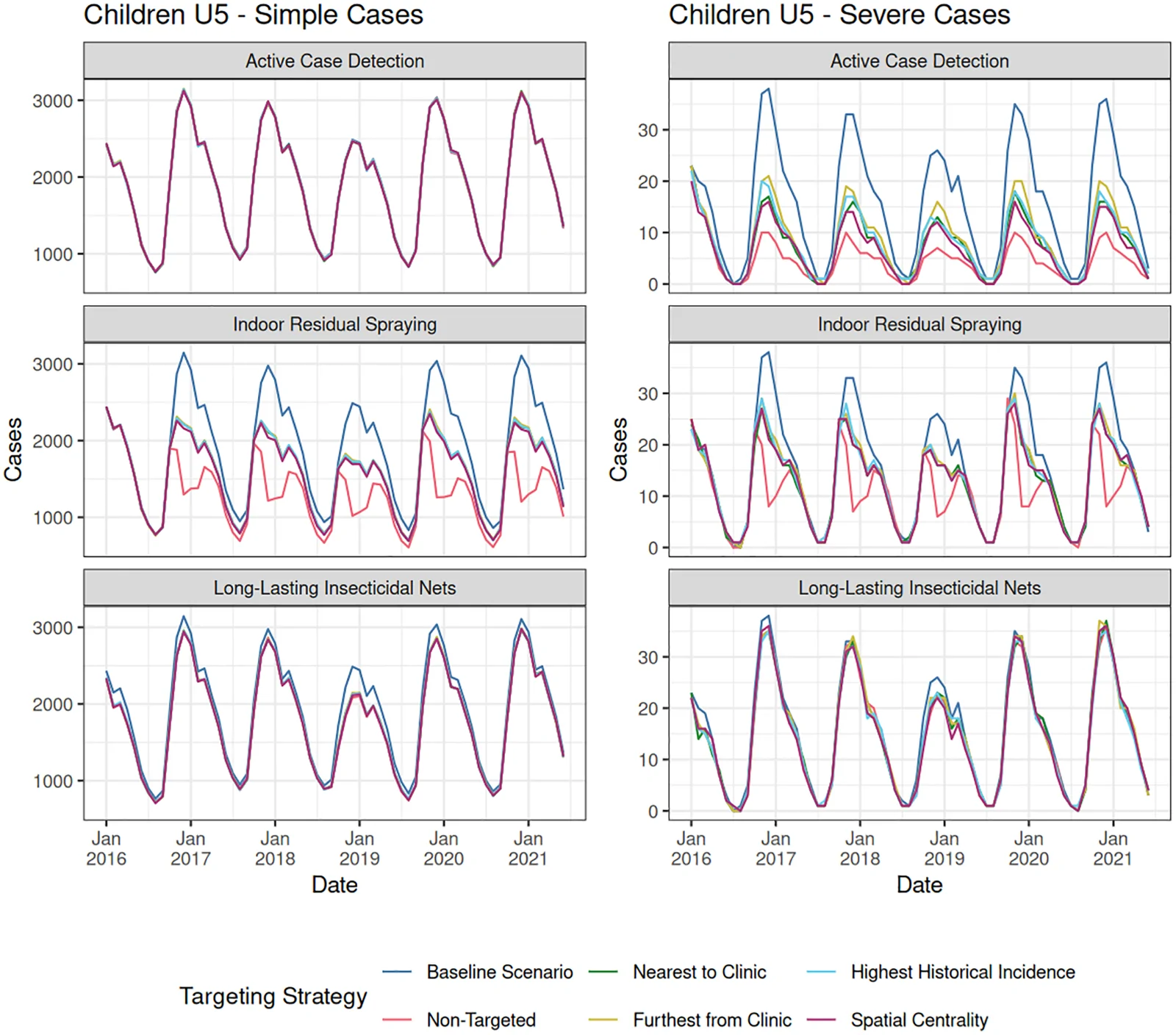
Non-targeted and Spatial Centrality targeting strategies tended to perform the best for all three interventions. Each line represents the median of 499 simulations of the number of cases for each combination of targeting strategy (line color) and intervention (row) for simple (left) and severe (right) malaria cases in children under 5 years old. Simulations are based on draws from the posterior of the best fit model. Adults are shown in Fig E in S1 Appendix.

When considering the resources required for each intervention, a similar number of houses were targeted for all IRS scenarios and similar sized populations were reached by all ACD campaigns (Table 3). However, the Non-Targeted scenario had the highest resource use with regards to transport distance to the nearest health clinic or district hospital. It included distances to clinics over three times that of the Nearest to Clinic scenario, which had the smallest total distance (Table 3). The Spatial Centrality scenario resulted in the largest population covered by ACD, covering over 25,000 more individuals than the Non-Targeted scenario, while maintaining the lowest overall distance to the district hospital (Table 3). There was little difference in the number of houses included in IRS across all five scenarios (Table 3), while the number of houses included in LLIN campaigns ranged from 17504 to 28807 depending on the scenario (Table 3).

**Table 3:** The equally distributed targeting scenario had the highest transport needs, while covering similar numbers of households and populations as other interventions. An estimation of resources required for each spatial targeting scenario based on the travel distances needed to implement the intervention, the number of households covered by the intervention, and the total population covered by the intervention.

	Total Distance from Clinic (km)	Total Distance from District Hospital (km)	Houses Targeted for IRS	Houses Targeted for LLIN	Population Covered by ACD
Equally Distributed	1375.22	12514.99	11523	28807	126848
Nearest to Clinic	368.56	6545.26	12508	20847	125881
Furthest from Clinic	1006.66	5969.72	10537	17562	106004
Historical Incidence	615.15	6468.6	11333	18889	115562
Spatial Centrality	585.61	4812.27	13698	22830	155260

We evaluated different coverage levels and combinations of interventions to identify scenarios that could be used by stakeholders to eliminate malaria in the district. The most effective scenario at averting simple cases was a combination of all three interventions at high coverage using a Non-Targeted strategy, which averted 56.88% of adult cases and 57.24% of childhood cases, on average (Table D in S1 Appendix). The Non-Targeted scenario continued to perform best, outperforming other targeting scenarios at all levels of coverage and intervention combinations (Table D and Fig G and H in S1 Appendix). With regards to severe cases, any scenario that included ACD at high coverage levels and was Non-Targeted was able to avert 100% of severe cases for both age classes (Fig G and H in S1 Appendix)Those scenarios using Spatial Centrality targeting performed second best, averting between 60.14 – 73.27% of cases when including ACD at high coverage levels, while there was little difference between the remaining three targeting scenarios (Table D in S1 Appendix).

An accompanying web application displays the results of intervention scenarios at fokontany and district levels (Fig 5). Users can choose which combination of interventions, spatial targeting scenario, and coverage level to consider and evaluate the resulting impact on simple and severe cases compared to a base scenario. It also displays spatial patterns in the number of cases averted. These functionalities were part of a larger effort to inform local decision making in malaria control by combining an early warning system [44] with scenarios of interventions into the same application.

**Fig 5:**
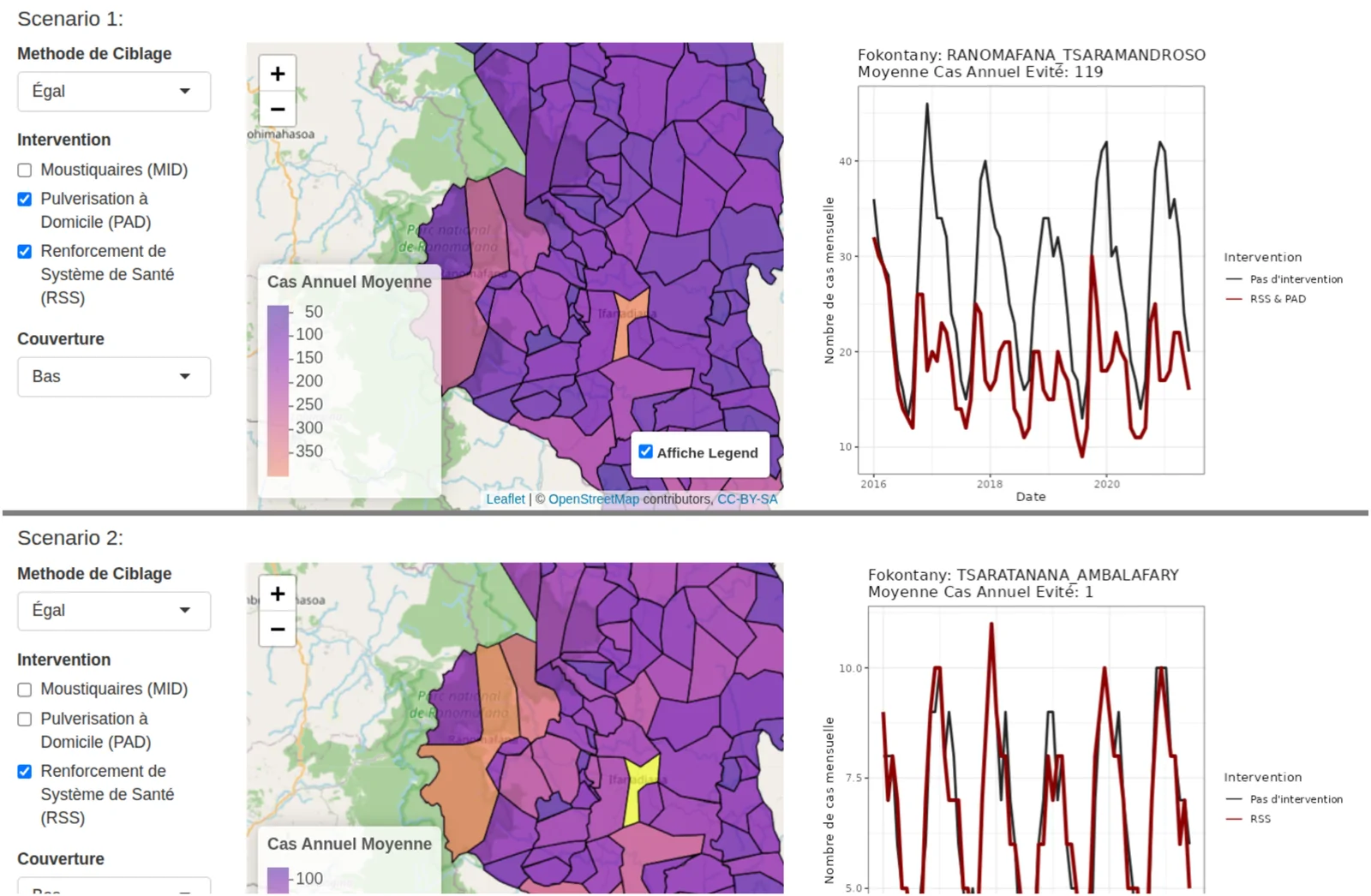
Screenshot of web application displaying the results of different scenarios of combinations of interventions and spatial targeting. The application is accessible at https://smaller.pivot-dashboard.org/ on the Interventions tab. Basemap Source: OpenStreetMap (https://www.openstreetmap.org). Boundary Source: UN OCHA (https://data.humdata.org/dataset/cod-ab-mdg).

## Discussion

As malaria control progress slows and funding decreases, spatially-targeted interventions offer a potential solution to distribute limited resources more efficiently. However, evidence regarding the effectiveness of spatially-targeted interventions is mixed, and dependent on the spatial scale of the targeting. Designing spatially-targeted control programs within a health district could allow for local tailoring of national-level interventions and result in more effective interventions. We developed a spatially-structured dynamic model of malaria for a rural district in southeastern Madagascar to compare different spatial targeting strategies of three common malaria interventions conducted at the community-scale. Notably, the model was based on high-resolution, spatio-temporal environmental data and used transport networks to inform spatial structure. The model was fit to field-derived prevalence and incidence data at the community-scale. Our modeling exercise illustrates the importance of including spatial heterogeneity in the modeling of malaria at the community-scale, particularly with regards to environmental forcing. We find that non-targeted interventions tend to perform better than or equally as well as spatially-targeted interventions across all combinations of interventions in this high transmission setting. However, spatial targeting that takes into account the spatial distribution of community catchments and their populations can be as effective as non-targeted interventions in certain cases while mobilizing over 60% fewer resources. This provides evidence for the utility of using spatial structure to guide tailored intervention campaigns, rather than the conventional approach of hotspot identification.

Our results demonstrate the importance of including local spatial structure in the environmental forcing variables of a dynamic malaria model. This was particularly true for vector and pathogen traits that were temperature dependent, given the wide-range of temperatures seen across the district and the large number of traits that were temperature-sensitive. This agrees with prior work identifying temperature as a strong driver of spatial heterogeneity in malaria transmission through both mechanistic [58,59] and statistical models [60–62]. In addition, the temperature range seen across the district (15–27 C) corresponds with the range with the highest amount of variation and uncertainty in thermal response curves for *Anopheles* mosquitoes and malaria [63]. Rainfall and flooding data were available at coarser resolutions and the traits dependent on these variables were less sensitive to spatial variation across the range seen in the district (Fig C in S1 Appendix). Although commonly incorporated into statistical models (e.g., [36,64,65]), few mechanistic models include multiple environmental forcing variables at once ([66], but see [40,67]). This has been applied even more rarely at local, community scales, where climate data may be limited [68], although satellite-based climate data and models, such as ERA5 or MODIS, are becoming more easily accessible. Given the increasing availability of this data, future work modeling malaria transmission via dynamical or mathematical models should include spatially-explicit environmental forcing dynamics when possible.

We found that including realistic spatial structure via a distance matrix increased model performance, regardless of whether this structure was based on a naive model of Euclidean distance or a more complex model that included true road networks. This is in contrast to prior work that found that including true road networks resulted in better estimates of geographic barriers to care than euclidean distances [69–71]. However, distance in our model was scaled to range from 0 - 1, and not a direct calculation of travel time based on the distance. As suggested by established theory [72], the mobility matrices based on both datasets were therefore highly correlated and resulted in similar mobility and malaria dynamics. This suggests that the simpler euclidean distance network can be used when a true road network is not available and absolute distances or travel times are not necessary for the mobility model. However, our networks did not include information on road condition or type, which could greatly impact travel time. Including such information would likely reduce the correlation between the two datasets and provide more accurate estimates of perceived distance. Data on inter-fokontany movement is not available for Ifanadiana district, and we were therefore unable to validate whether this term accurately represented true human movement or whether its inclusion in the model served more as a latent variable of spatial variation that represented spatial covariance in EIR and FOI not explained by the environmental forcing terms. Our finding that different models performed similarly suggests that it is the inclusion of any spatial structure, and associated unmeasured covariance, that improved model performance, rather than a specific model formulation, provided the parameters are flexible enough to fit the underlying spatial covariance. Overall, the general conclusion is the same: spatial structure in host and vector dynamics should be considered when modeling malaria at the community scale.

In spite of the importance of spatial structure to the dynamical model, non-targeted interventions tended to be the most effective. While much effort has been put into the identification of malaria “hotspots” to guide targeting, evidence for the effectiveness of spatially targeted interventions is mixed [14,73]. Spatial targeting has proven useful in malaria elimination zones, where individual cases or clusters of cases may act as a source for an outbreak (e.g., the Zanzibar Malaria Elimination Programme, [74]), but may be less useful in areas with heterogeneous, but overall high, rates of malaria transmission, where these hotspots may not lead to increased malaria transmission in surrounding areas [75]. Given the high case rates of malaria seen in Ifanadiana (over 100 cases per 1000 population annually, [76]), it is likely that overall transmission rates are too high for hotspot targeting to be effective, even at the community-scale. However, it is notable that the best performing spatially targeted intervention was not based on case incidence or prevalence, as is often used in hotspot targeting, but on the underlying spatial structure of the transport network by targeting the most central patches. This is in agreement with our finding in the sensitivity analysis that interventions are more successful for more mobile populations. Tun et al. [56] found that above-optimal MDA coverage in highly connected patches can counteract sub-optimal coverage in other patches using mathematical models, leading to a net success. In addition, source-sink dynamics of malaria have been observed at the national scale in Madagascar [77], and the increased effectiveness of interventions at higher mobility rates may be related to similar dynamics at a fine scale, allowing interventions to impact neighboring regions when mobility is high. Our results agree with this and suggest that incorporating spatial patterns in population density and movement when allocating resources could be more effective than guiding interventions using historical disease case rates, if spatial targeting is to be used.

Concerning the ideal community-scale intervention scenario for this region of Madagascar, our results suggest that interventions that target the vector itself, particularly IRS, are most effective at controlling simple malaria cases. The performance of IRS relative to LLIN campaigns is likely due to some assumptions of our model regarding LLIN effectiveness in Madagascar and the difficulty of modeling indirect and direct effects of LLINs on malaria prevalence (e.g., [78]). Following field data in Madagascar, we assumed mosquitoes were 30% endophilic [79], with the majority of biting occurring outside the home where LLINs provide little to no protection [80]. We also included a waning pesticide efficacy and maximum rate of correct use and pesticide-induced mortality that limited the effectiveness of LLINs. Finally, because LLIN coverage was already at 50% in the district, it could not be increased in simulations as much as IRS, which is currently not practiced in the district. Our results should not be interpreted to mean that LLIN distribution campaigns should be decreased or that they should no longer strive for universal coverage, as these campaigns have averted over 400 million cases [81]. Rather, LLIN-related interventions should focus on specific strategies to increase their effectiveness. For example, in Ifanadiana district, the IHOPE survey has found household coverage rates of over 90%, however fewer than 50% of households meet the WHO recommendation of one net per two people. Increased returns on LLIN-based interventions may therefore be more likely by focusing on increasing use rates themselves through sensibilizations or ensuring net quality, rather than simply increasing the household-level coverage of distribution campaigns, which can have limited returns at very high coverage values [82]. A highly-mobile population further complicates a LLIN intervention, as individuals are not likely to travel with their own LLIN, resulting in individuals losing LLIN protection or households exceeding the WHO recommendation. In instances where travel is repeated, such as to a farm or rice field, additional nets could be installed in the temporary residence and LLIN distribution campaigns could take this into consideration during the census stage. With regards to resource use, the Non-Targeted scenarios required the most travel distance from clinics and the district hospital, a major source of costs in IRS and LLIN campaigns. The Spatial Centrality scenario required about a third of the travel distance of the Non-Targeted scenario, and was able to cover a similar amount of households. Decision makers should therefore take into account the trade-off between the effectiveness of district-wide interventions and their potential costs, depending on their budget and requirements. None of the interventions were able to fully eliminate malaria cases, although ACD was able to avert all severe malaria cases when it was at 100% coverage. Madagascar’s current strategy aims to increase case detection and treatment rates by expanding malaria community case management to all ages and allowing proactive case detection by CHWs and other health teams. Our simulation results support this as an effective intervention given the high rates of endemicity, agreeing with pilot field studies that tested these interventions in neighboring regions of Madagascar [83,84].

While our dynamic model was complex, it necessarily simplified several aspects of malaria transmission dynamics. We did not include individual-level immunity nor model the parasite within human hosts, both of which have been shown to be important for malaria transmission models [85,86]. In particular, immunity can interact with environmental forcing to drive malaria seasonality in endemic regions such as Ifanadiana [87,88]. We instead included these dynamics using a simplified two-class age model with differing rates for adults and children and allowing for an age-specific waning immunity rate, given the computational limitations of explicitly modeling 192 patches at a daily timestep, which currently requires 22,500 CPU-hours per model. Similarly, our ability to account for multiple co-occuring malaria genotypes, as has been seen elsewhere in Madagascar [89], was limited by computational resource constraints. Omitting this co-circulation may underestimate the malaria burden in areas with high forces of infection, potentially lowering the effectiveness of Incidence-based Targeting in our model. Our inclusion of a pathway for sub-clinical cases to become re-infected partly accounts for this, but does not allow for the tracking of superinfections and their impact on malaria dynamics [90]. The model also did not include spatial structure in existing interventions, particularly access to care and LLIN use, which exhibit strong spatial heterogeneity across the district [91]. These behaviors may change over time in unpredictable ways in response to perceived risk [15]. We therefore chose to use a district-level mean for the existing ACD and LLIN coverage rates, based on field-collected data. This could potentially influence our findings regarding the spatially-targeted interventions if, for example, some patches already have very high or low levels of coverage. However, given the clear superiority of Non-Targeted interventions, we expect this difference would primarily be qualitative and patch-specific. In addition, focusing on a district benefiting from a long-term intervention may limit the generalizability of our results to other districts, where existing treatment rates are much lower than in Ifanadiana. However, our sensitivity analysis found similar qualitative results for simulated districts with lower baseline treatment rates. Our field data was further altered via the ZERO-G adjustment to reduce the known geographical and financial barriers to care in the district [33]. The ZERO-G method is itself a statistical model that minimizes differences in incidence rates across financial interventions and levels of geographic isolation. Although it aims to reduce known biases, it necessarily introduces other sources of uncertainty in the final incidence estimates, resulting in more realistic, but not perfect, data. This uncertainty propagates to the absolute numbers of cases averted; however, given the large qualitative differences between interventions, we do not believe this alters the primary conclusions of this study. Applying this method to data from other malaria endemic contexts could further test the robustness of these conclusions.

Our study agrees with prior work that finds that non-targeted interventions are the most effective at averting malaria cases in endemic regions. However, we also find that they are the most costly, particularly with regards to travel and transport. Spatially targeted interventions, particularly those based on population spatial structure and not historical incidence, were nearly as effective as non-targeted interventions at over half the travel costs. Incorporating the underlying spatial structure of the population could be a more efficient use of resources when they are limited, and should be explored in other contexts. We explored three interventions currently being used in Madagascar, but future work could apply a similar framework to more recent interventions, particularly pharmaceutical interventions such as vaccination (e.g., [92]), seasonal chemoprevention (e.g., [93]), or treatments with longer lasting prophylactic protection periods, to guide targeting strategies. Our study also highlights the potential of CHWs to implement community-based interventions. However, past work in Madagascar has found the success of community malaria interventions, particularly ACD, is highly dependent on a strengthened logistics chain to prevent stock disruptions and financial support of CHWs [94,95]. Implementing community-based, spatially-targeted interventions will therefore require investment in community health programs to ensure CHWs have the additional resources needed to successfully to serve in these roles.

## Supporting information

S1 Appendix Appendix containing containing supplemental Text A, supplemental Tables A-D, and supplemental Figures A–H. (PDF)
