# Supplementary material for: Leveraging spatial structure to design spatially-targeted malaria interventions at the community-scale
Source: PLoS Comput Biol. 2026 Oct 15;22(10):e1014857. doi: 10.1371/journal.pcbi.1014857 (PMC13690273; doi:10.1371/journal.pcbi.1014857)
Supplement: Supplementary file 1 [file pcbi.1014857.s001.pdf]

S1 APPENDIX

Leveraging spatial structure to design spatially-targeted malaria interventions at the community-scale

Michelle V Evans, Benjamin Roche, Vincent Herbreteau, Christophe Révillion, Thibault Catry, Matthew H. Bonds, Karen E. Finnegan, Ezra Mitsinjoniala, Felana A. Ihantamalala, Mauricianot Randriamihaja, Oméga Raobela, Andres Garchitorena

Table of Contents

Supplemental Text A. French Language Abstract/ Resumé Français

Supplemental Table A. Model parameters and primary sources for values.
Supplemental Table B. Variation in transition rates due to environmental forcing
Supplemental Table C. Averted cases with low baseline ACD at high and low ACD intervention coverage.
Supplemental Table D. Averted cases for combined interventions at high and low coverage levels.

Supplemental Figure A. Map of study area of Ifanadiana District, Vatovavy, Madagascar
Supplemental Figure B. Comparison of ZERO-G adjustment
Supplemental Figure C. Variation in environmental forcing by malaria season.
Supplemental Figure D. Probability of movement in mobility models.
Supplemental Figure E. Adult cases averted when applying one intervention by targeting strategy.
Supplemental Figure F. Comparison of targeting strategy effectiveness across different mobility levels.
Supplemental Figure G. Cases averted for children under 5 years old across differing combinations of interventions, targeting strategies, and coverage levels
Supplemental Figure H. Cases averted for adults across differing combinations of interventions, targeting strategies, and coverage levels

TEXT A. FRENCH LANGUAGE ABSTRACT/RESUME FRANCAIS

Exploiter la structure spatiale pour concevoir des interventions ciblées contre le paludisme à l'échelle communautaire

Les progrès dans la lutte contre le paludisme stagnent depuis le début du XXIe siècle dans de nombreux pays, ce qui nécessite de nouvelles approches telles que le recours à des interventions ciblées spatialement. Cependant, les données concernant l'efficacité de ces interventions sont mitigées, et dépendent à la fois du contexte et du type d'intervention. De plus, le rôle potentiel des programmes de santé communautaire dans la mise en œuvre du ciblage spatial reste sous-exploré, les modèles existants se concentrant sur des échelles spatiales plus grossières que celles nécessaires pour orienter les activités programmatiques. Nous avons développé un modèle de paludisme à deux classes d'âge, spatialement explicite, à l'échelle communautaire pour un district du sud-est de Madagascar, tenant compte de l'hétérogénéité environnementale et la structures spatiale, afin d'étudier les interventions de santé communautaire ciblées spatialement. Le modèle a été paramétrisé avec des notifications de cas et la sero-prévalence du paludisme, puis utilisé pour simuler trois interventions : la pulvérisation intradomiciliaire à effet rémanent (PID), la couverture supplémentaire des moustiquaires imprégnées d'insecticide de longue durée (MID) et la détection active des cas (DAC): Nous avons comparé cinq scénarios de ciblage spatial pour chaque intervention simulée : l'un sans ciblage spatial et les autres ciblant les communautés (i) les plus proches des cliniques, (ii) les plus éloignées des cliniques, (iii) présentant l'incidence la plus élevée et (iv) occupent une position centrale sur le plan géographique. L'intervention non ciblée s'est généralement révélée la plus efficace, mais la moins économe en ressources. La deuxième intervention la plus efficace était fondée sur la centralité spatiale ; elle a permis de toucher une population plus large tout en mobilisant moins de ressources de transport que l'intervention non ciblée. Aucune combinaison d'interventions n'a permis d'éradiquer le paludisme dans le district, bien qu'une intervention qui détecte 100 % des cas puisse prévenir 100 % des cas de paludisme grave. Ces résultats soulignent le potentiel des interventions ciblées contre le paludisme, en particulier dans les milieux à faibles revenus, qui tiennent compte de la structure spatiale de la population humaine et le réseau routier afin de réduire la charge de morbidité liée au paludisme en utilisant moins de ressources que les interventions conventionnelles menées à l'échelle du district.

Table A. Model parameters and primary sources for values.

Symbol	Description	Value/Priors	Source
HOST EQUATION PARAMETERS			
γ_A	Movement out of subclinical compartment. Inverse of patent period of malaria	Prior: 0.0001-0.1	Estimated
γ_P	Movement from treated to susceptible. Inverse of protected period provided by prophylaxis	1/25	Griffin et al. 2010
α	Movement from exposed to infected. Inverse of incubation period	1/12	Griffin et al. 2010
s^*	Age-dependent symptomatic rate	Children: 0.29<br>Adult: 0.089	IHOPE 2021
τ_s^*	Age-dependent treatment rate of symptomatic cases	Children: 0.57<br>Adult: 0.33	IHOPE 2021
g^*	Age-dependent severe case rate	Children: 0.029<br>Adult: 0.006	Kamau et al. 2020
γ_S	Movement from symptomatic to asymptomatic hosts. Inverse of clinical period.	1/5	Griffin et al. 2010
τ_A^*	Age-dependent treatment rate of asymptomatic cases	Children: 0.0030<br>Adult: 0.0015	Assuming 1 consultation per year for adults and 2 for children, with RDT sensitivity of 0.56.
γ_G	Movement from severe to treated. Inverse of severe clinical period.	1/14	Bretscher et al. 2020
β_H^*	Age-dependent transmission rate from vector to host	Children: 0.59<br>Adult: 0.3	Griffin et al. 2010
c	Scaling parameter for concurrent infection	Prior: 0.0001 – 0.9999	Estimated
p_m	Probability of remaining in source patch	Prior: 0.0001 – 0.9999	Estimated

VECTOR EQUATION PARAMETERS			
$b(T)$	Eggs per day	f(Temperature)	Mordecai et al. 2013
$\varepsilon(T)$	Larval development rate	f(Temperature)	Mordecai et al. 2013
$\mu_A(T)$	Adult mortality rate	f(Temperature)	Mordecai et al. 2013
$\Delta(T)$	Parasite development rate	f(Temperature)	Mordecai et al. 2013
$a(T)$	Bite rate	f(Temperature)	Mordecai et al. 2013
β_V	Class-dependent transmission rate from host to vector.	Prior:<br>Symptomatic: 0.001 – 0.3	Estimated symptomatic rate.<br>Asymptomatic: Held at 30% of symptomatic<br>Treated: Held at 5% of symptomatic
L_f	Fractional larval growth state	0.5	Approximates average larval life stage
K_{flush}	Maximum mortality rate due to flushing from rainfall	0.4	Tompkins and Ermert 2013
T_{flush}	Scaling parameter to define the shape of the relationship between rainfall and larval mortality	0.5	Tompkins and Ermert 2013
K	Carrying capacity of flooded rice field (individuals/m^2)	Prior: 10 - 35	Estimated

Table A References

Bretscher, M. T., P. Dahal, J. Griffin, K. Stepniewska, Q. Bassat, E. Baudin, U. D'Alessandro, A. A. Djimde, G. Dorsey, E. Espié, B. Fofana, R. González, E. Juma, C. Karema, E. Lasry, B. Lell, N. Lima, C. Menéndez, G. Mombo-Ngoma, C. Moreira, F. Nikiema, J. B. Ouédraogo, S. G. Staedke, H. Tinto, I. Valea, A. Yeka, A. C. Ghani, P. J. Guerin, and L. C. Okell. 2020. The duration of chemoprophylaxis against malaria after treatment with artesunate-amodiaquine and artemether-lumefantrine and the effects of pfmdr1 86Y and pfcrt 76T: a meta-analysis of individual patient data. BMC Medicine 18:47.

Griffin, J. T., T. D. Hollingsworth, L. C. Okell, T. S. Churcher, M. White, W. Hinsley, T. Bousema, C. J. Drakeley, N. M. Ferguson, M.-G. Basáñez, and A. C. Ghani. 2010. Reducing Plasmodium falciparum Malaria Transmission in Africa: A Model-Based Evaluation of Intervention Strategies. PLOS Medicine 7:e1000324.

Kamau, A., G. Mtanje, C. Mataza, G. Mwambingu, N. Mturi, S. Mohammed, G. Ong'ayo, G. Nyutu, A. Nyaguara, P. Bejon, and R. W. Snow. 2020. Malaria infection, disease and mortality among children and adults on the coast of Kenya. Malaria Journal 19.

Mordecai, E. A., K. P. Paaijmans, L. R. Johnson, C. Balzer, T. Ben-Horin, E. de Moor, A. McNally, S. Pawar, S. J. Ryan, T. C. Smith, and K. D. Lafferty. 2013. Optimal temperature for malaria transmission is dramatically lower than previously predicted. Ecology Letters 16:22–30.

Tompkins, A. M., and V. Ermert. 2013. A regional-scale, high resolution dynamical malaria model that accounts for population density, climate and surface hydrology. Malaria Journal 12:65.

Table B. Variation in Transition Rates due to Environmental Forcing

Environmental Variable	Parameter	Minimum Daily Variation	Median Daily Variation	Maximum Daily Variation	Mean Daily Coefficient of Variation
Flooding	Mosquito Carrying Capacity (millions), $W*K$	4.5998	9.6621	14.9341	0.8328
Temperature	Parasite Development Rate , $\Delta(T)$	0.0382	0.0485	0.0548	0.1559
Temperature	Larval Development Rate, $\varepsilon(T)$	0.0364	0.0472	0.0537	0.1545
Temperature	Eggs per Female per Day, $b(T)$	2.7743	5.2403	8.6745	0.1431
Temperature	Bite rate, $a(T)$	0.1145	0.1345	0.1656	0.1198
Temperature	Larval Mosquito Survival, $\mu_L(T)$	0.0618	0.2592	0.6927	0.0993
Rainfall	Flushing-Induced Larval Mortality, $\mu_L(R)$	0	0	0.2989	0.0063
Temperature	Adult Mosquito Survival, $\mu_A(T)$	0.0056	0.0174	0.0528	0.0041

Table C. Averted cases with low baseline ACD at high and low ACD intervention coverage.

	Case Type	ACD Coverage	Non-Targeted	Nearest to Clinic	Furthest from Clinic	Historical Incidence	Spatial Centrality
		low	278 (216 - 328)	316 (257 - 361)	234 (164 - 308)	264 (207 - 303)	346 (322 - 362)
	Simple	high	605 (565 - 672)	344 (301 - 390)	282 (243 - 302)	312 (264 - 362)	382 (314 - 471)
Children		low	282 (257 - 303)	294 (265 - 312)	244 (212 - 274)	274 (253 - 291)	320 (291 - 340)
Under 5	Severe	high	540 (494 - 579)	334 (300 - 366)	281 (253 - 302)	306 (280 - 331)	364 (327 - 398)
		low	485 (285 - 627)	503 (411 - 587)	354 (238 - 434)	447 (308 - 505)	600 (469 - 701)
	Simple	high	892 (792 - 958)	500 (283 - 623)	487 (400 - 570)	565 (409 - 770)	620 (513 - 687)
		low	64 (57 - 69)	66 (56 - 70)	55 (45 - 63)	60 (52 - 64)	72 (63 - 77)
Adults	Severe	high	121 (105 - 130)	73 (65 - 78)	62 (56 - 67)	67 (61 - 72)	79 (69 - 85)

Table D. Averted cases for combined interventions at high and low coverage levels.

		Interventions				Targeting Scenario				
		IRS	LLIN	ACD	Coverage Level	Non-Targeted	Nearest to Clinic	Furthest from Clinic	Historical Incidence	Spatial Centrality
Children Under 5	Simple Cases	X			low	7916 (7811 - 8202)	3214 (3109 - 3305)	3239 (3164 - 3341)	3288 (3262 - 3314)	3476 (3320 - 3609)
		X			high	11075 (10867 - 11480)	4231 (4094 - 4397)	4307 (4205 - 4378)	4309 (4206 - 4411)	4555 (4481 - 4621)
			X		low	1651 (1227 - 2341)	749 (531 - 1080)	802 (573 - 1105)	858 (614 - 1250)	881 (638 - 1225)
			X		high	3305 (2533 - 4754)	1458 (1113 - 2058)	1528 (1171 - 2131)	1664 (1301 - 2331)	1619 (1236 - 2337)
				X	low	165 (27 - 244)	79 (3 - 164)	92 (13 - 174)	216 (115 - 286)	131 (65 - 202)

				X	high	361 (284 - 424)	261 (213 - 314)	212 (83 - 316)	257 (216 - 304)	191 (86 - 249)
		X	X		low	9502 (9103 - 9876)	3754 (3545 - 4003)	3739 (3565 - 4000)	3751 (3590 - 3967)	4002 (3827 - 4248)
		X	X		high	13596 (13098 - 14090)	4953 (4707 - 5242)	5008 (4854 - 5306)	5122 (4850 - 5447)	5349 (5109 - 5737)
			X	X	low	1806 (1365 - 2569)	1008 (741 - 1370)	953 (746 - 1226)	945 (722 - 1314)	976 (745 - 1332)
			X	X	high	3566 (2833 - 5035)	1592 (1216 - 2212)	1681 (1331 - 2333)	1635 (1236 - 2311)	1789 (1365 - 2472)
		X		X	low	8137 (7994 - 8472)	3301 (3139 - 3383)	3303 (3213 - 3403)	3512 (3428 - 3624)	3607 (3523 - 3695)
		X		X	high	11294 (11107 - 11713)	4308 (4179 - 4395)	4470 (4375 - 4583)	4514 (4364 - 4621)	4716 (4614 - 4793)
		X	X	X	low	9599 (9247 - 9926)	3800 (3652 - 4086)	3919 (3725 - 4130)	3856 (3576 - 4151)	4084 (3938 - 4252)
		X	X	X	high	13758 (13247 - 14245)	5130 (4826 - 5434)	5158 (4910 - 5448)	5306 (5044 - 5630)	5550 (5337 - 5899)
	Severe Cases	X			low	52 (45 - 56)	25 (14 - 32)	22 (14 - 33)	26 (23 - 32)	24 (17 - 28)
		X			high	74 (67 - 79)	26 (16 - 30)	31 (26 - 39)	26 (24 - 29)	33 (25 - 39)
			X		low	8 (4 - 10)	3 (0 - 5)	1 (0 - 8)	8 (3 - 13)	7 (3 - 17)
			X		high	25 (20 - 32)	4 (1 - 9)	10 (6 - 19)	15 (11 - 20)	9 (6 - 14)
				X	low	121 (107 - 130)	54 (50 - 58)	58 (52 - 65)	69 (58 - 77)	72 (66 - 74)
				X	high	164 (148 - 175)	80 (68 - 86)	86 (77 - 91)	94 (81 - 101)	99 (89 - 102)
		X	X		low	72 (70 - 74)	30 (25 - 34)	24 (19 - 30)	25 (21 - 31)	26 (23 - 29)
		X	X		high	99 (95 - 101)	32 (29 - 39)	30 (24 - 34)	34 (30 - 37)	34 (25 - 41)

			X	X	low	125 (113 - 134)	65 (63 - 68)	68 (60 - 74)	70 (63 - 75)	77 (65 - 83)
			X	X	high	164 (148 - 175)	79 (70 - 85)	91 (85 - 97)	94 (83 - 99)	103 (95 - 111)
		X		X	low	137 (122 - 147)	72 (65 - 76)	73 (67 - 79)	89 (82 - 94)	89 (78 - 96)
		X		X	high	164 (148 - 175)	91 (83 - 96)	102 (93 - 108)	106 (96 - 117)	113 (104 - 120)
		X	X	X	low	142 (127 - 149)	72 (64 - 82)	80 (71 - 85)	80 (71 - 89)	85 (77 - 92)
		X	X	X	high	164 (148 - 175)	96 (89 - 101)	98 (90 - 106)	107 (101 - 115)	110 (98 - 117)
Adults	Simple Cases	X			low	19743 (18530 - 21210)	8398 (8181 - 8730)	8547 (8293 - 8984)	8586 (8264 - 9061)	9097 (8764 - 9675)
		X			high	26808 (24653 - 28862)	10879 (10542 - 11383)	11023 (10593 - 11618)	11048 (10632 - 11631)	11689 (11165 - 12357)
			X		low	4209 (3446 - 5899)	1930 (1611 - 2731)	2036 (1740 - 2816)	2139 (1827 - 2900)	2190 (1848 - 3014)
			X		high	8474 (7007 - 11620)	3820 (3165 - 5226)	3926 (3266 - 5475)	4086 (3358 - 5503)	4208 (3575 - 5712)
				X	low	270 (124 - 364)	73 (0 - 307)	106 (48 - 169)	282 (139 - 376)	146 (92 - 222)
				X	high	725 (615 - 795)	434 (389 - 458)	454 (329 - 560)	416 (353 - 520)	364 (314 - 418)
		X	X		low	23384 (21630 - 24804)	9642 (9165 - 10040)	9787 (9355 - 10162)	9834 (9408 - 10228)	10349 (9803 - 10826)
		X	X		high	32560 (29702 - 34576)	12653 (11897 - 13143)	12928 (12372 - 13391)	13106 (12415 - 13566)	13775 (13110 - 14246)
			X	X	low	4492 (3728 - 6199)	2292 (1986 - 3133)	2234 (1943 - 2911)	2129 (1771 - 2912)	2290 (1915 - 3152)
			X	X	high	8968 (7492 - 12123)	4122 (3424 - 5608)	4291 (3649 - 5638)	4280 (3674 - 5676)	4484 (3741 - 5867)

		X		X	low	20032 (18738 - 21528)	8532 (8265 - 9000)	8565 (8271 - 9103)	8960 (8583 - 9355)	9216 (8830 - 9771)
		X		X	high	27212 (25073 - 29274)	11112 (10635 - 11686)	11366 (10881 - 12005)	11449 (10922 - 11985)	12105 (11622 - 12790)
		X	X	X	low	23430 (21820 - 24861)	9738 (9258 - 10132)	9970 (9604 - 10255)	9949 (9601 - 10318)	10387 (9845 - 10758)
		X	X	X	high	32864 (29939 - 34956)	13043 (12520 - 13529)	13188 (12522 - 13674)	13417 (12854 - 13871)	14098 (13349 - 14626)
	Severe Cases	X			low	24 (19 - 29)	12 (5 - 15)	13 (7 - 19)	13 (10 - 15)	16 (14 - 17)
		X			high	38 (34 - 44)	17 (14 - 23)	16 (12 - 20)	14 (10 - 19)	18 (13 - 22)
			X		low	6 (4 - 11)	2 (0 - 4)	3 (0 - 8)	2 (0 - 7)	7 (2 - 13)
			X		high	13 (9 - 19)	3 (0 - 10)	6 (3 - 10)	10 (4 - 14)	3 (0 - 11)
				X	low	29 (25 - 32)	13 (9 - 15)	14 (9 - 17)	17 (10 - 22)	19 (15 - 22)
				X	high	70 (64 - 75)	36 (33 - 42)	36 (33 - 39)	41 (36 - 48)	42 (36 - 48)
		X	X		low	37 (32 - 39)	15 (10 - 20)	14 (11 - 18)	14 (10 - 16)	15 (8 - 21)
		X	X		high	48 (43 - 51)	18 (17 - 19)	16 (12 - 21)	18 (10 - 22)	15 (12 - 19)
			X	X	low	33 (29 - 36)	21 (15 - 24)	21 (15 - 24)	19 (12 - 24)	21 (15 - 25)
			X	X	high	70 (64 - 75)	35 (30 - 40)	39 (36 - 42)	41 (37 - 44)	46 (41 - 49)
		X		X	low	45 (41 - 50)	25 (21 - 28)	24 (21 - 28)	33 (30 - 36)	29 (23 - 33)
		X		X	high	70 (64 - 75)	42 (36 - 47)	46 (41 - 50)	46 (44 - 50)	51 (48 - 54)
		X	X	X	low	50 (44 - 54)	26 (22 - 28)	26 (24 - 30)	27 (26 - 29)	28 (25 - 30)
		X	X	X	high	70 (64 - 75)	43 (38 - 51)	44 (39 - 49)	48 (45 - 54)	50 (47 - 55)

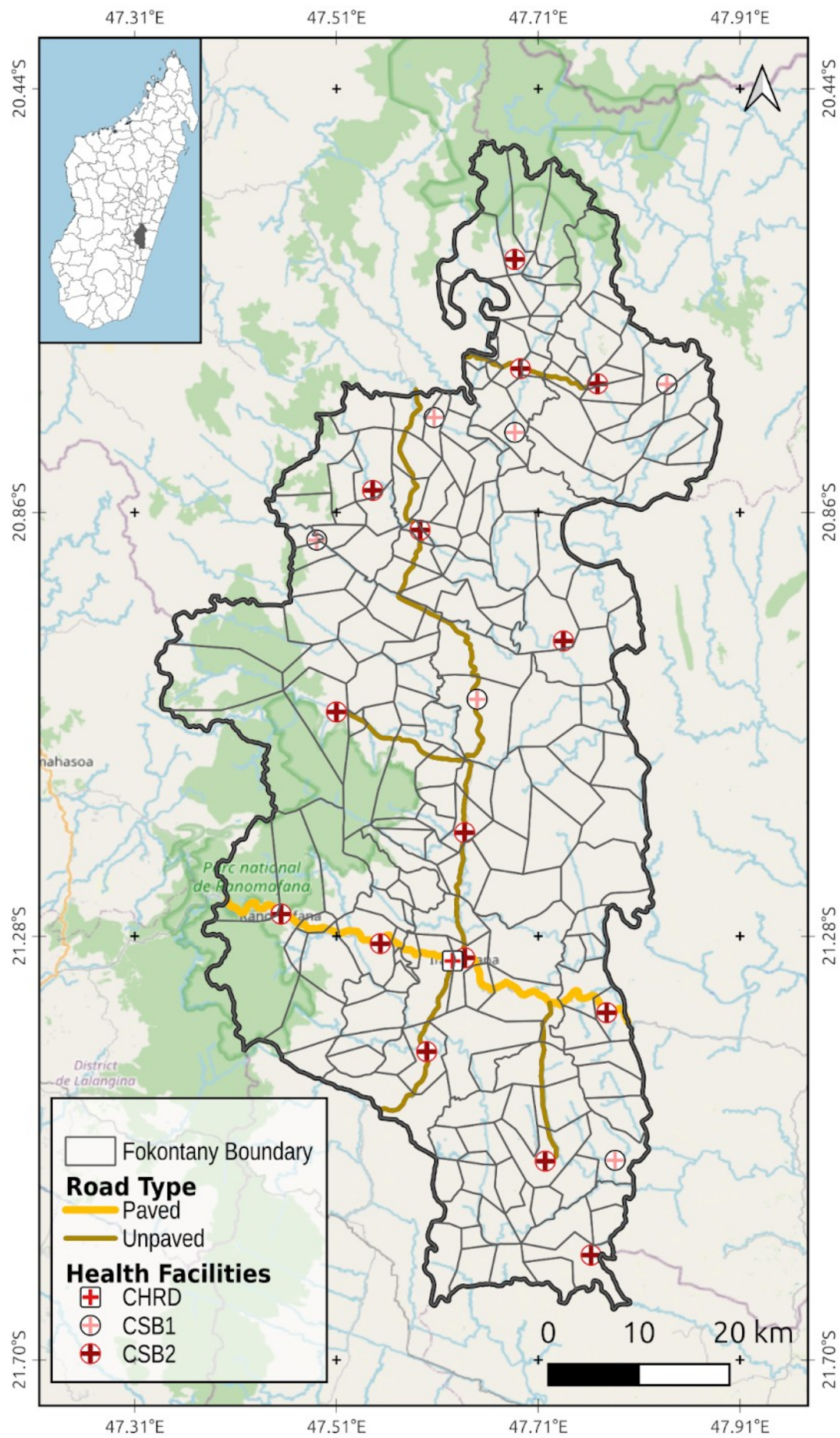

Fig A. Map of study area of Ifanadiana District, Vatovavy, Madagascar with primary transport network and health clinics. Inset map shows district's location within Madagascar. Basemap Source; OpenStreetMap (https://www.openstreetmap.org).

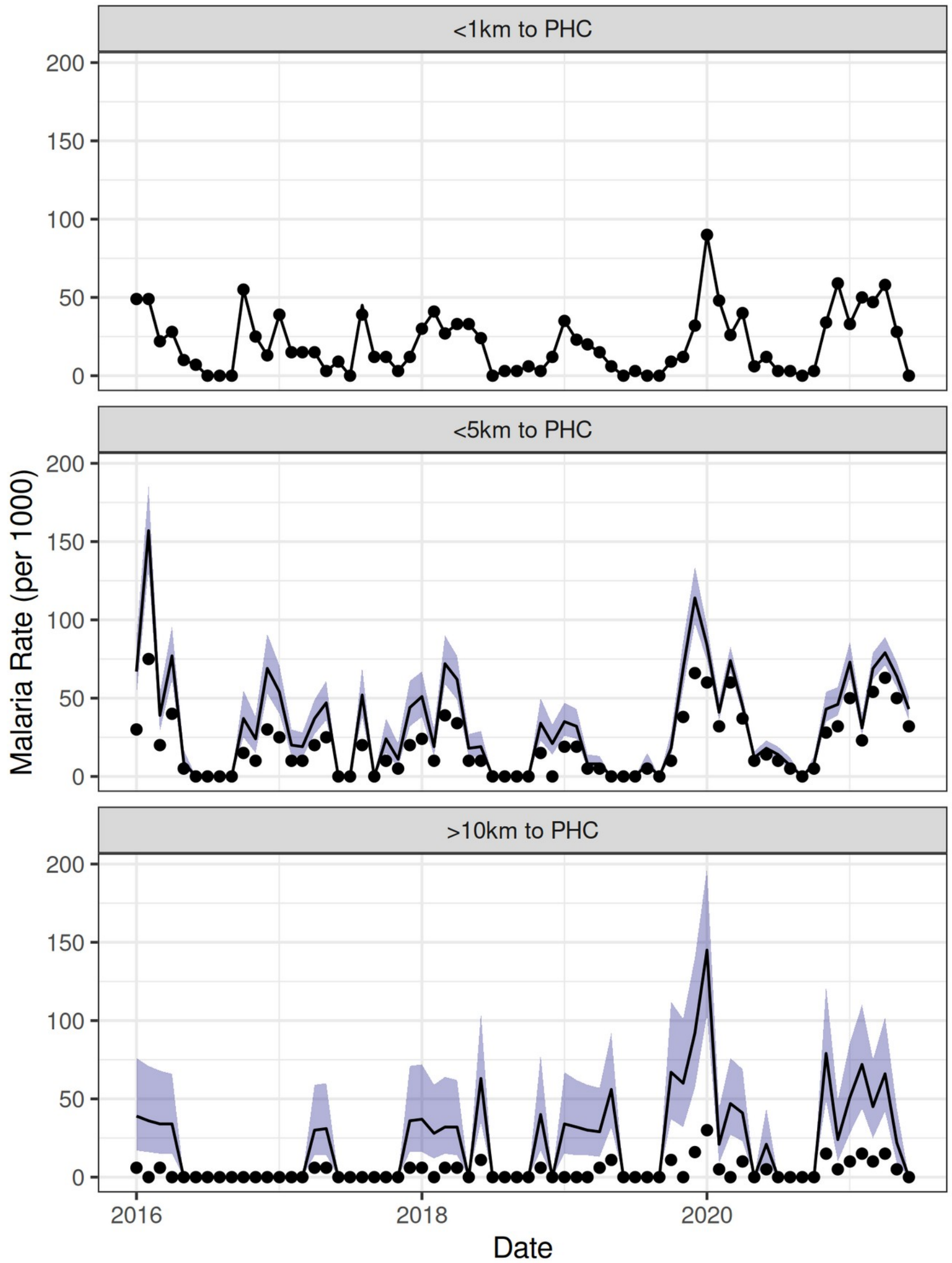

Fig B. Comparison of ZERO-G adjustment for a fokontany within 1 km of a primary health center (PHC), within 5 km of a PHC, and greater than 10km from a PHC within one commune. Points represent the raw malaria incidence rate, lines represent the adjusted rate, and shaded error bars represent 95% confidence intervals.

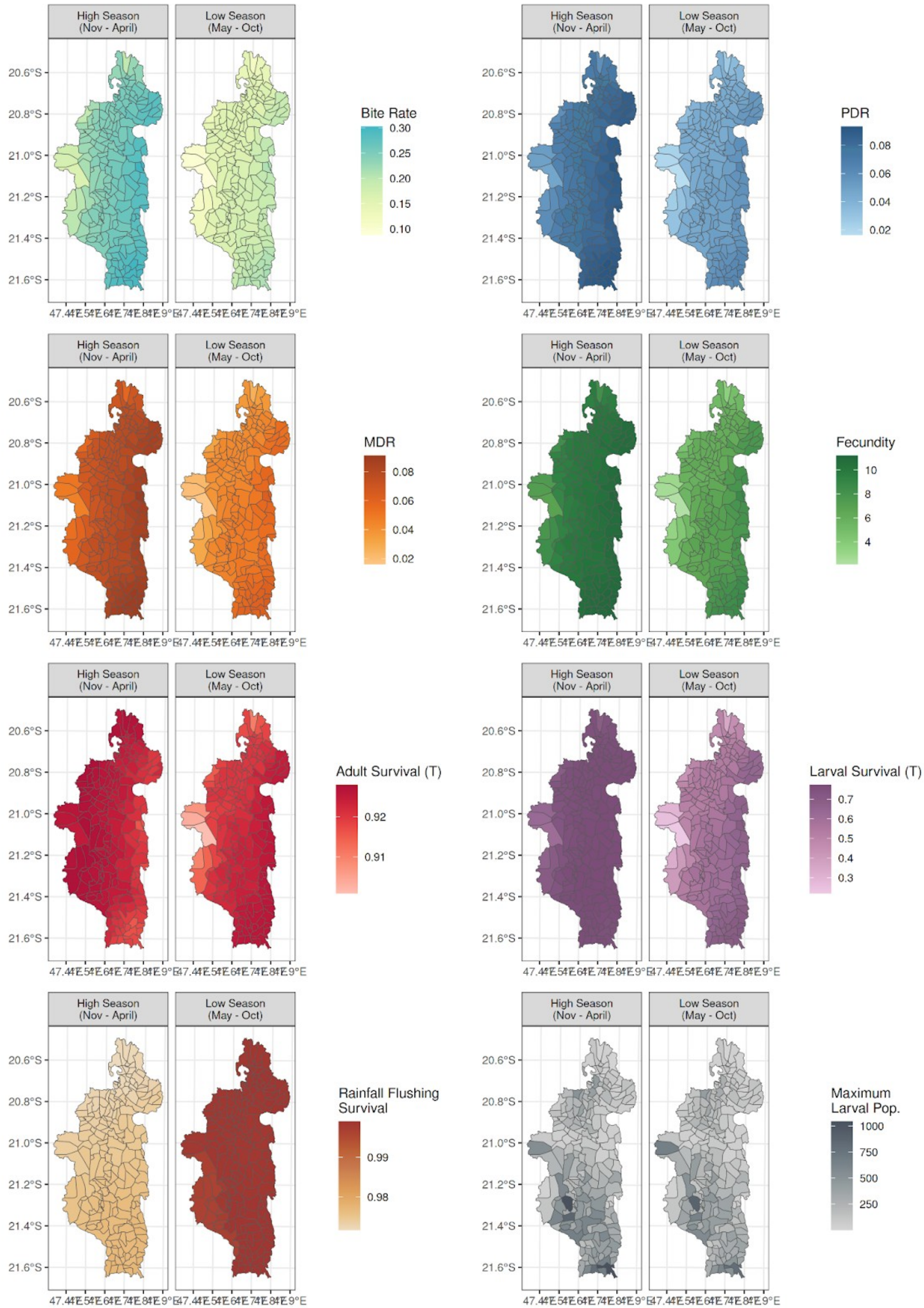

Fig C. Variation in environmental forcing by malaria season. Administrative boundaries represent fokontany limits (Source: OCHA).

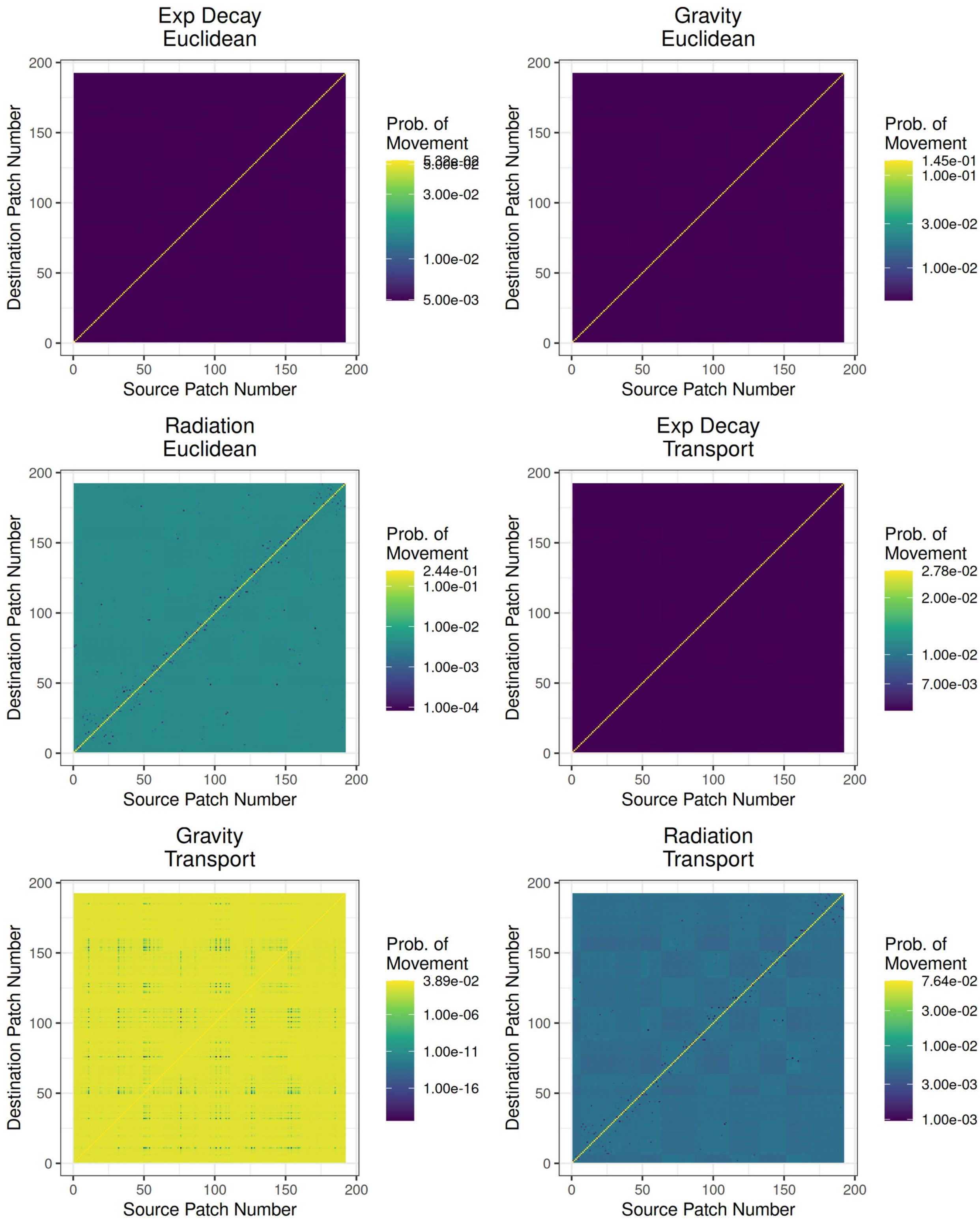

Fig D. Probability of movement from source patch i to destination patch j for the best fit parameter set of each mobility model. Color bars are represented on the log scale to aid with visibility.

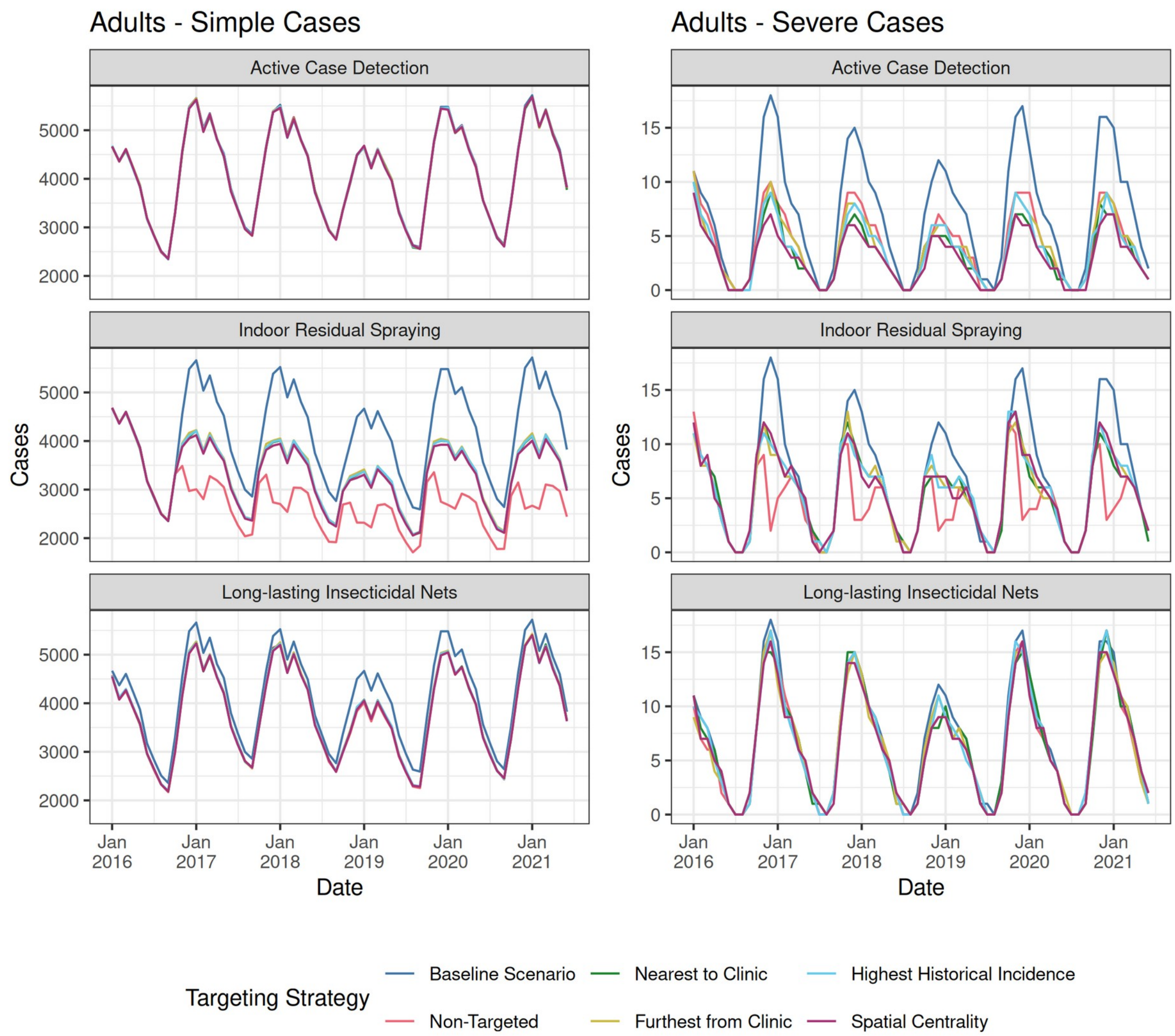

Fig E. Adult cases averted when applying one intervention by targeting strategy.

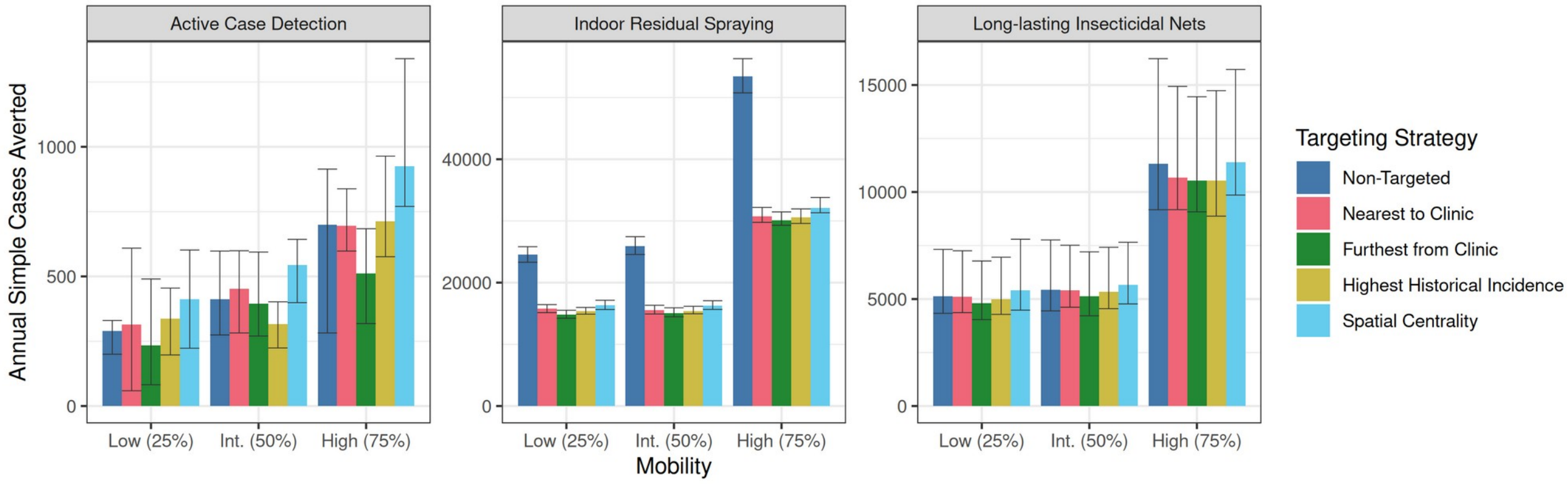

Fig F. Comparison of targeting strategy effectiveness across different mobility levels.

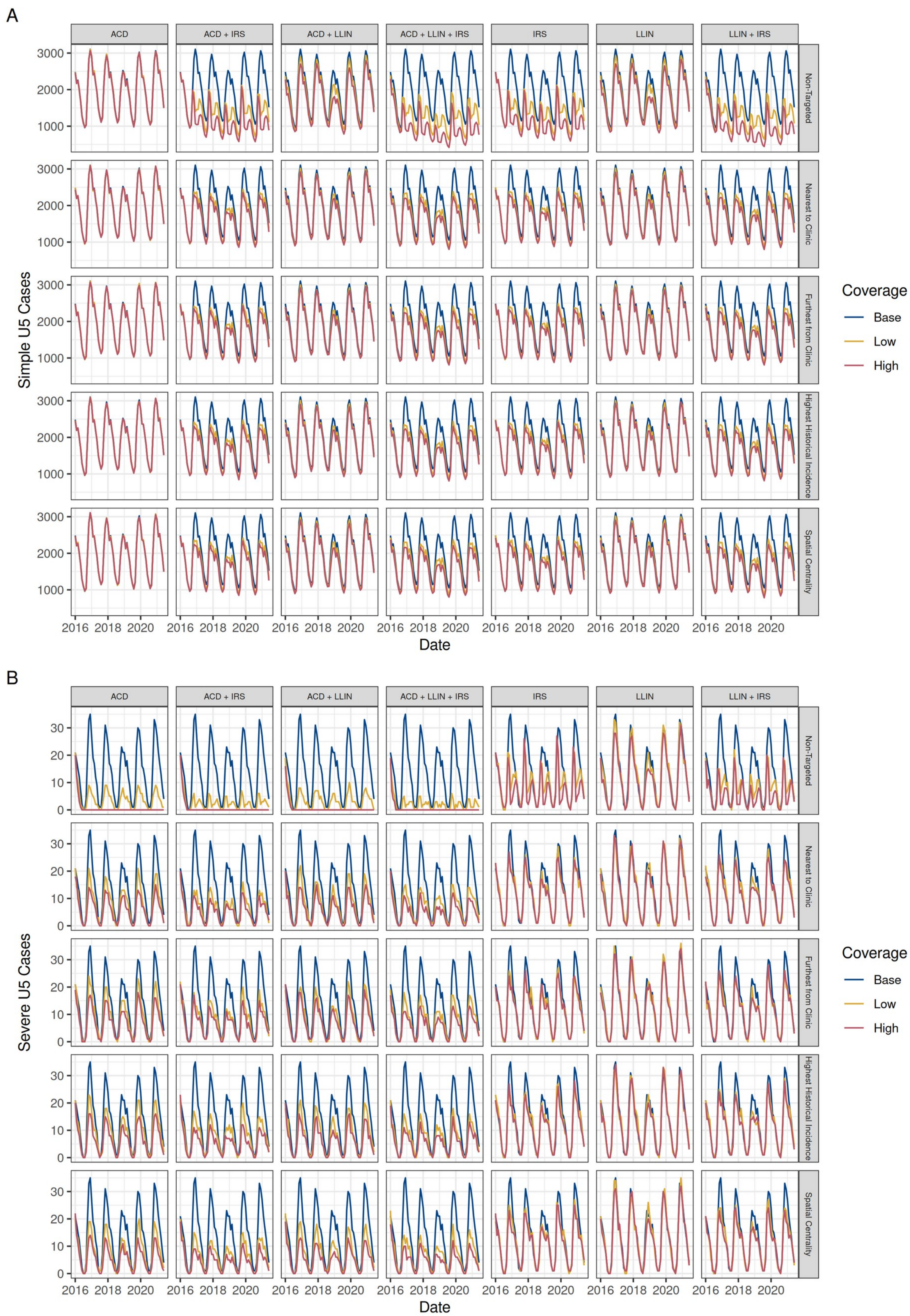

Fig G. Cases averted for children under 5 years old across differing combinations of interventions, targeting strategies, and coverage levels. Base coverage is the current coverage levels of interventions, as defined in the main

manuscript. Low coverage is equal to 1.5x base levels or 100% coverage, whichever is lower. High coverage is equal to 2x base levels or 100% coverage, whichever is lower. Coverage is only increased in targeted fokontany, with other fokontany retaining base coverage levels.

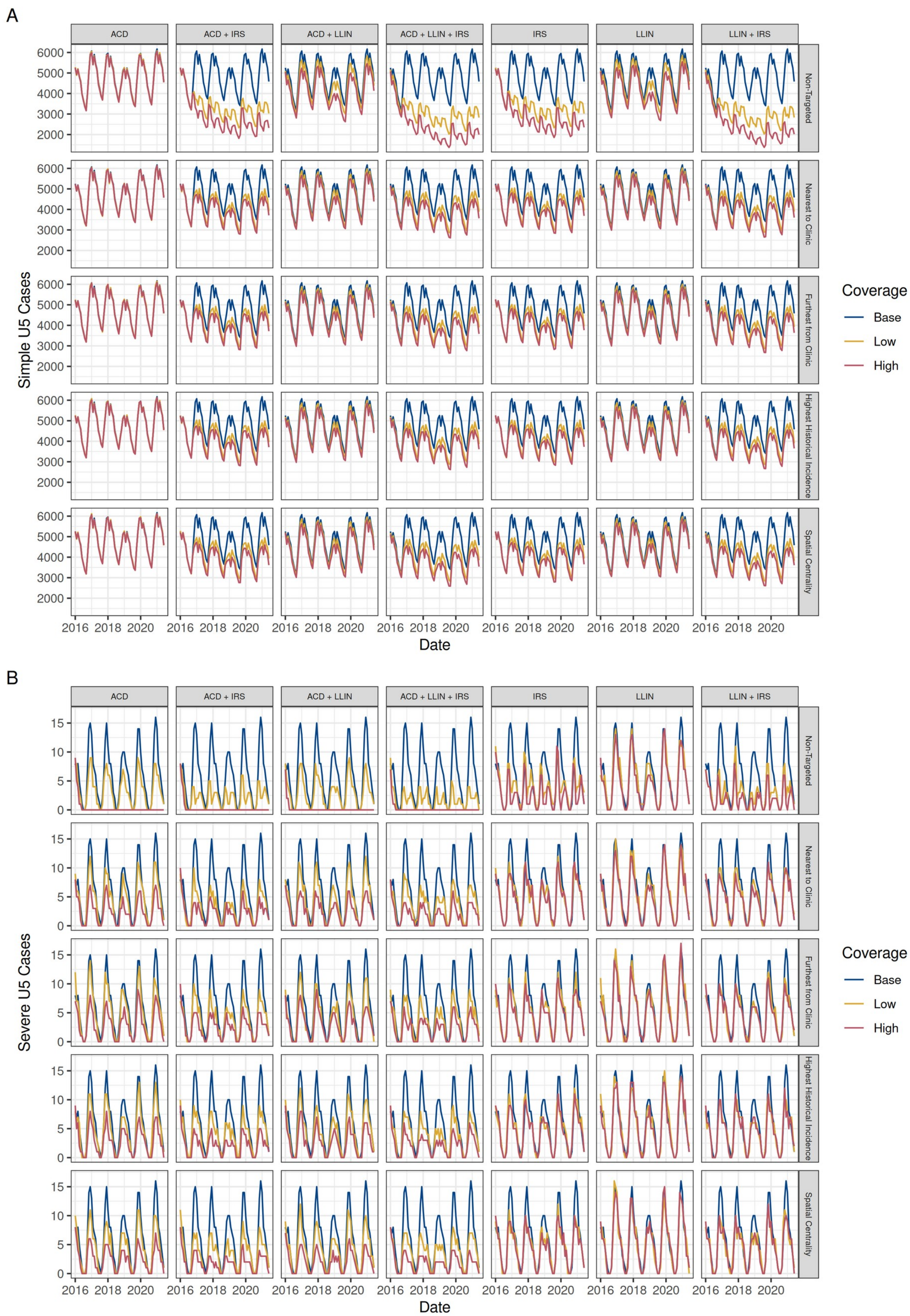

Fig H. Cases averted for adults across differing combinations of interventions, targeting strategies, and coverage levels. Base coverage is the current coverage levels of interventions, as defined in the main manuscript. Low

coverage is equal to 1.5x base levels or 100% coverage, whichever is lower. High coverage is equal to 2x base levels or 100% coverage, whichever is lower. Coverage is only increased in targeted fokontany, with other fokontany retaining base coverage levels.
