# Supplementary material for: Leveraging spatial structure to design spatially-targeted malaria interventions at the community-scale
Source: PLoS Comput Biol. 2026 Oct 15;22(10):e1014857. doi: 10.1371/journal.pcbi.1014857 (PMC13690273; doi:10.1371/journal.pcbi.1014857)
Supplement: Supplementary file 2 [file pcbi.1014857.s004.pdf]

RÉPUBLIQUE FRANÇAISE
Liberté
Égalité
Fraternité

Institut de Recherche pour le Développement
FRANCE

July 3, 2026

Re: Submission ID PCOMPBIOL-D-26-00387

Dear Editor:

Please find enclosed with this letter our revised manuscript "Leveraging spatial structure to design spatially-targeted malaria interventions at the community-scale" which we resubmit for publication as a Research Article in *PLoS Computational Biology*.

Your comments and those of the reviewers were insightful and informative, and greatly enhanced the quality of our manuscript. In the following pages are our responses to each of the comments.

Reviewers' comments are written in **bold** serif font, with our replies directly following. Revisions in the text are shown using Track Changes and line numbers correspond to line numbers in that document. We hope that the revisions and our accompanying responses will be sufficient to make our manuscript suitable for publication in *PLoS Computational Biology*.

Thank you for your consideration.

Sincerely,
Michelle Evans, PhD

We confirm that this manuscript is original and has not been published, is not in press, and is not under review elsewhere. All `R` code needed to reproduce the analysis will be deposited on Figshare upon acceptance.

Reviewer #1: Review of: Leveraging spatial structure to design spatially-targeted malaria interventions at the community-scale by Evans et al
Manuscript Number: PCOMPBIOL-D-26-00387
Journal: PLOS Computational Biology
2026 March 06

Summary:
Evans et al present a model-based exploration of targeting malaria interventions, parameterizing their model with data from a district in Madagascar. Three intervention types are investigated for a suite of spatial targeting strategies, and are compared to a non-targeted scenario. Such work is timely, as stalled progress in controlling malaria and funding shortages are necessitating conversation about new approaches to the deployment of interventions. The reviewer commends the authors on a well-written manuscript and thorough development of a model incorporating much of the complexity of the system. If the authors are able to address a few major and some minor comments, it is the opinion of this reviewer that this work could be an important contribution to our understanding of malaria epidemiology in the present moment. See below for the comments organized by section, with major comments focusing on (i) the mobility analyses, where improvements are needed to clarify for the reader and aid in reproducibility, (ii) potential circularity/bias in estimating the effectiveness of bednet distributions, and (iii) accounting for immunity (or the lack thereof) in malaria transmission models

Major comments:

(1) Mobility - This section of the study would benefit from greater clarity.

First, the methods section on mobility (starting at line 261) was difficult to follow. Are data on observed rates of movement by human hosts in this district included in the study? As it currently reads I think movement is assumed, and just straight line distances or distances along paths is considered but could the authors clarify? If just using distance, "distance dataset" is more appropriate I believe than "mobility data" (e.g., Lines 270, 292 etc), which implies some measurement of actual movement. Likewise "daily commuting" was confusing to this reviewer, and may be so to a general audience. I wouldn't expect much daily travel in a rural, subsistence agriculture area and 'daily' commuting implies people are sleeping at their residence (and given malaria is transmitted at night, daytime commuting would not be relevant to transmission).

In response to this comment and others regarding the methods section, we have restructured the methods section and adjusted our language so that it is clearer to the reader. It is difficult to follow via the Track Changes, but we now introduce the host and vector equations together, describe how they are linked via the mobility matrices, introduce specific aspects of environmental forcing for certain parameters, and then provide equations for the impact of IRS and LLIN on vector rates. This ensures parameters are not introduced before they are defined.

We had used the word “commuting” to signify that hosts remained residents of their patches and that that is where a positive case would be recorded, mirroring how cases are recorded by health facilities. We no longer use the term “commuting” to reduce confusion.

We do continue to define the matrix of movement probability as a mobility matrix, but clarify that it is based on distance datasets (line 325):

Mobility matrices *m* were created from two distance datasets that described the pairwise distance between each patch: a naive dataset representing the euclidean distance and a transport-network based distance dataset.

Additionally, could the authors report and assess the scale of travel they are seeing in their models? In other words, is the amount of travel needed to get the increased performance as seen in the summary statistics realistic? Is it consistent with data on actual observations of overnight intra-district travel? Or is inter-patch travel as included in the model capturing some other source of variation in malaria infection risk across the district (e.g., travel times correlate with bednet deployment success historically or something along those lines)? I ask because I struggle to see the biological mechanism underpinning the explanatory power of a distance-matrix here - would this not require a significant proportion of hosts to be spending nights in patches with different infection probabilities?

We unfortunately do not have quantitative data on inter-fokontany travel rates within the district. However, given the small size of fokontany, we know that anecdotally people do travel and sleep outside of their household, particularly during rice growing season where many sleep nearer their farms. The proportion of individuals moving ranges from 75 – 97%, depending on the mobility model, with a proportion of 92.57% for the highest performing mobility model. These values are quite high because they represent the sum of the probabilities of moving to 191 patches. When the probability of staying in a source patch is compared to the probability of moving to the most “attractive” destination patch, individuals are on average 20.4 times more likely to stay in their patch for the highest performing mobility model. We now provide supplemental figures of heatmaps of the mobility matrices (Supp. Fig. 4) to help viewers better interpret these models.

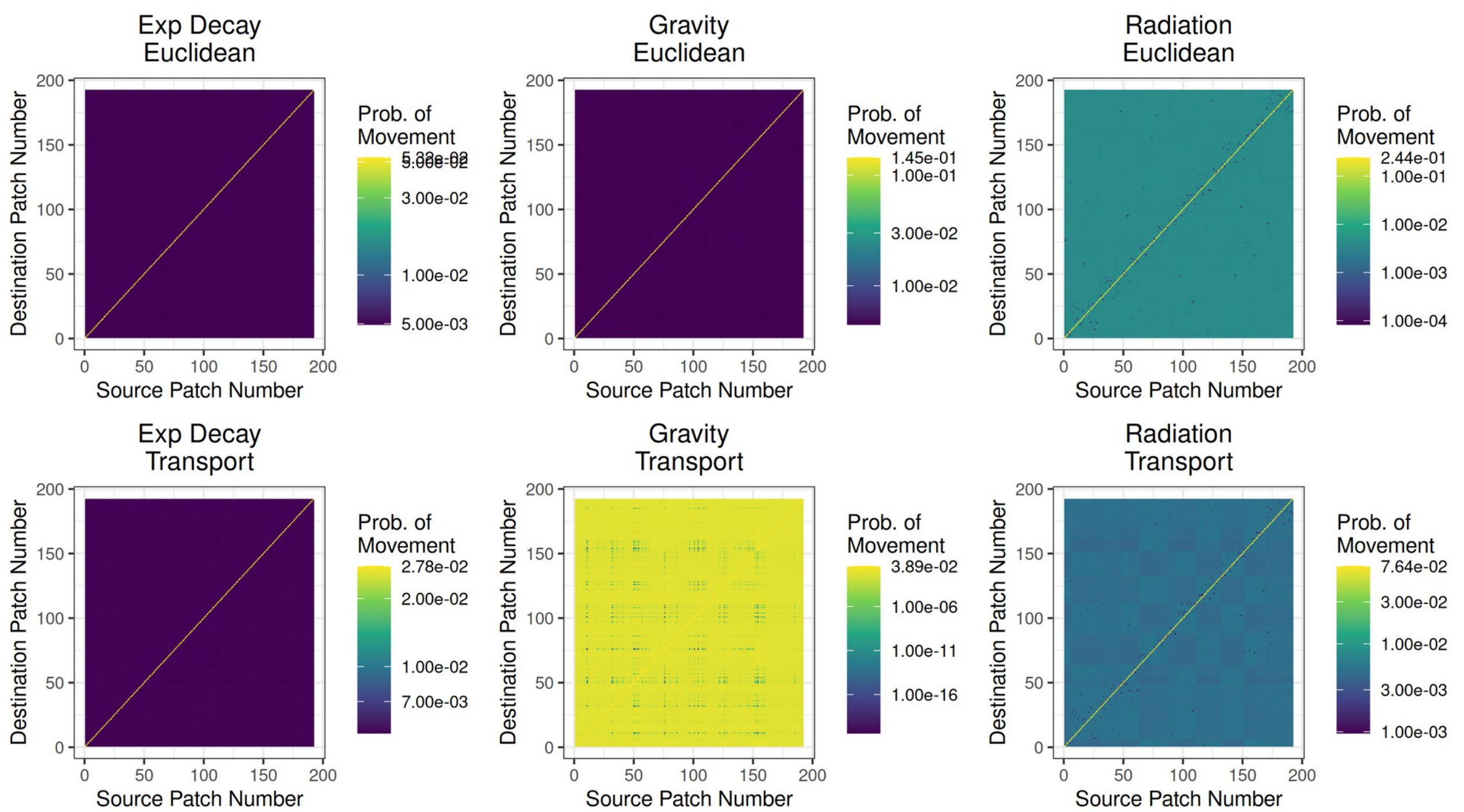

Supp. Fig. 4. Probability of movement from source patch *i* to destination patch *j* for the best fit parameter set of each mobility model. Color bars are represented on the log scale to aid with visibility.

In addition, in response to this and the comment of the other reviewer, we have conducted a sensitivity analysis to examine the impact of interventions at different levels of mobility. To do this, we took the best fit mobility model and fixed the proportion moving at three different

levels: 25%, 50%, and 75% We then simulated the three interventions 19 times at each level of mobility for each targeting strategy:

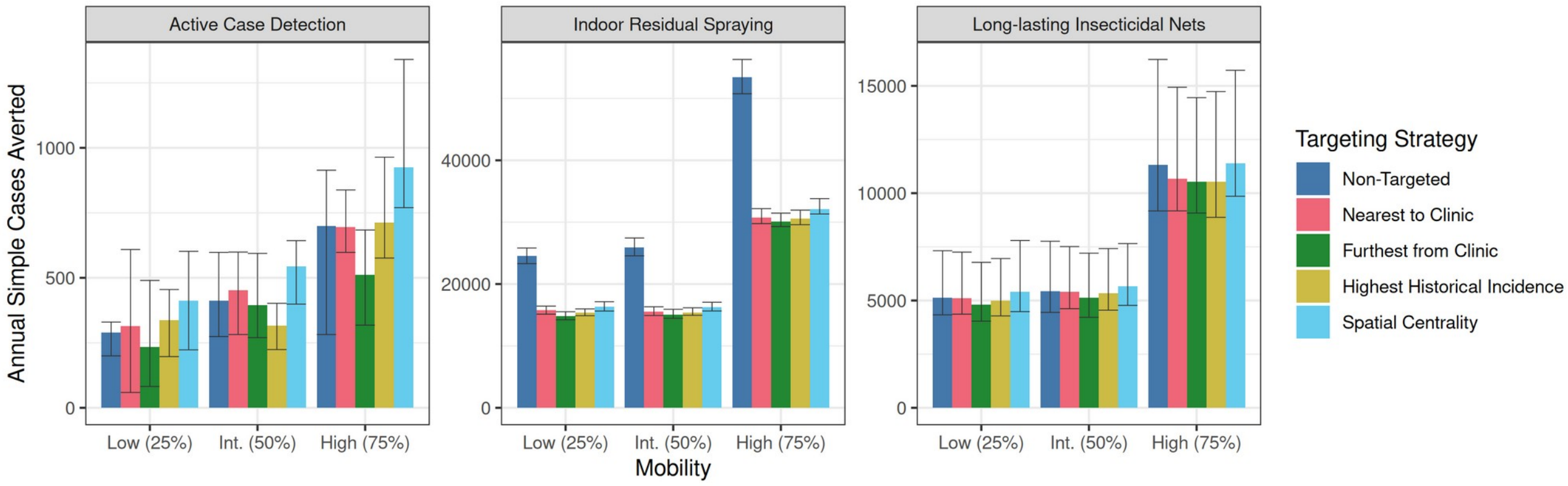

Fig. S6. Interventions were more effective at high mobility rates across all targeting scenarios.

Qualitatively, the results are similar, with the non-targeted scenario performing better or as well as the targeted scenarios. However, an interesting result from this sensitivity analysis if that all interventions perform best at high mobility levels, likely because the indirect effects of above-optimal intervention coverage levels can counteract sub-optimal coverage in nearby patches, resulting in a net positive effect at the district-level. This is discussed in the following places in the text:

Methods (line 534):

> Spatially-targeted malaria interventions can have indirect effects on disease in neighboring fokontany via “community-level” effects[55]. The direction of these effects are dependent on the relative difference in disease burden between patches and their connectivity[56]. We therefore conducted a sensitivity analysis to explore the relative effectiveness of different targeting strategies under different population mobility rates. Using the best fit mobility model, we fixed the probability of remaining in the source patch (p_m) while using all other mobility parameters as estimated. This resulted in three mobility levels: low (25% move), medium (50% move) and high (75% move). We then simulated the same combination of interventions and targeting strategies on each of these three scenarios and compared the average number of annually averted cases across 19 iterations.

Results (line 676):

> When considering differing mobility rates, we found that intervention effectiveness increased with increasing mobility rates for all targeting scenarios (Supp. Fig. 6).

Discussion (line 799):

> However, it is notable that the best performing spatially targeted intervention was not based on case incidence or prevalence, as is often used in hotspot targeting, but on the underlying spatial structure of the transport network by targeting the most central patches. This is in agreement with our finding that interventions are more successful for more mobile populations. Tun et al. [56] found that above-optimal MDA coverage in highly connected patches can counteract sub-optimal coverage in other patches using mathematical models, leading to a net success. Our results agree with this and suggest that incorporating spatial patterns in population density and movement when allocating resources could be more effective than guiding interventions using historical disease case rates, if spatial targeting is to be used.

Finally, we do agree that the inter-patch mobility could also represent an unmeasured latent factor that is characterized by similar spatial variation. This agrees with our more general conclusion that spatial structure results in more accurate models, whether it is a latent spatial term or explicitly defined via a mobility term that accounts for spatial structure. We have added more nuanced language to further address this (lines 781):

> Data on inter-fokontany movement is not available for Ifanadiana district, and we were therefore unable to validate whether this term accurately represented true human movement or whether its inclusion in the model served more as a latent variable of spatial variation that represented true spatial covariance in EIR and FOI not explained by the environmental forcing terms. However, the general conclusion is the same: spatial structure in host and vector dynamics should be considered at the community scale.

(2) Bednets as an intervention of focus

The observed case rate and malaria prevalence data are presumably measuring malaria infections that occurred on a background of variable but high bednet ownership and usage in the district historically. It is not clear how one can cleanly account for this. For example, it seems necessary to parse out the contribution of 'bednet-preventable cases' (the cases from those without a bednet but that would use a bednet if more were deployed) vs 'residual cases' (cases from those already with access to a bednet but that bednets alone failed to prevent, due to many other factors beyond outdoor biting) to total infection burden. I suspect that, unfortunately, much of the malaria burden that can be averted by bednets is already being averted and is therefore baked into the estimates of infection and biting rates that inform FOI in the model. One would need a 'clean' estimate of what FOI and biting rates would be if 'untainted' by existing bednet usage in the area to fully explore the benefits of bednets. The IRS and ACD interventions avoid this concern as they have not yet been implemented in the district, so I suggest focusing further on those

Yes, we agree with this comment. In fact our hypothesis is that this is why bednets are relatively ineffective in our intervention simulations, as bednets are already in use at relatively high levels. We do already include the current bednet coverage (i.e. proportion of households reporting sleeping under a bednet the prior night and owning at least one bednet per two household residents, the WHO definition) in the base model without interventions, using bednet coverage values derived from the longitudinal cohort survey as described in Eqs. 16 & 28 and lines 278-295. The current coverage rate is 50%, which necessarily limits the amount that it can be increased. In addition, it is further scaled by a time-dependent decay in net condition and proper use condition following a distribution campaign and a relatively high outdoor biting rate of 70%.

Given that we do include the current bednet coverage rate in the model, it would be possible to run a counterfactual simulation of no bednet use, to examine the amount of cases averted due to the current bednet use. However, in our opinion, this is a different question than that posed by our study, which is about which supplemental interventions could help reduce malaria burdens in a local context where efforts are already underway, as is the case of most rural districts in SSA. Given that no districts in Madagascar, and elsewhere, have 0% coverage, this would complicate the interpretation of our results. That said, we have added additional language to ensure that the interpretation of our results with regards to bednet campaigns is not used to lower campaign universal coverage goals (see text directly below next comment).

From an epidemiological perspective - universal coverage by bednets from mass distribution

campaigns has been the core of malaria control for a while. The scenarios explored here seem to be a retreat from that. Rather than a new "targeted" approach, the authors seem to suggest restricting bednet distribution below the current coverage, for the first time foregoing areas that would typically be included in distribution efforts. There seem to be both ethical considerations (removing public health goods from communities that have become reliant on them) and a broader problem: if the malaria control program is no longer able to even try to distribute bednets across districts where it normally does so this would undoubtedly be associated with a broader system collapse and a fundamentally different malaria control regime than was in place over the time series considered in this analysis

We have clarified in the text that we are not advocating for a retreat from universal coverage of bednets. Indeed, this is the goal of current distribution campaigns in Madagascar. However, our data has found that over multiple years of sampling, this has not been achieved, with on average 44.77% (range: 9.3 – 77.18%) of households reporting sleeping under a bednet the prior night and owning at least one bednet per two household residents, per the WHO definition. We are therefore advocating for a stronger investment, not only in bednet distribution, but in education campaigns and behavioral changes, so that actual use rates meet the claimed goals of universal coverage. We have added clarifying language to line 819 to ensure our results are not misinterpreted or could be used to reduce the frequency or intensity of existing campaigns:

> Our results should not be interpreted to mean that LLIN distribution campaigns should be decreased or that they should no longer strive for universal coverage, as these campaigns have averted 400 million cases [74]. Indeed, in Ifanadiana district, the IHOPE survey has found household coverage rates of over 90%, however fewer than 50% of households meet the WHO recommendation of one net per two people. Increased returns on LLIN-based interventions may therefore be more likely by focusing on increasing proper use rates themselves, rather than simply increasing the household-level coverage of distribution campaigns, which can have limited returns at very high coverage values [75].

Finally, 'subnational tailoring' of bednet distributions has been explored before. For example see work from Thomas Churcher's group in eLife (https://elifesciences.org/), where they use a statistical approach to estimate the impact of changing bednet distribution strategy, avoiding some of the pitfalls outlined above and below. There are probably limitations to that approach as well, but it speaks to the general difficulty of focusing the present study on bednets given the approach and data available

Thank you for sharing this work, Churcher's group has greatly contributed to research exploring malaria interventions and creating counterfactual scenarios to help guide policy. We now cite this work in our discussion of the interpretation of our bednet results, and the difficulty in modeling them (line 811):

> The performance of IRS relative to LLIN campaigns is likely due to some assumptions of our model regarding LLIN effectiveness in Madagascar and the difficulty of modeling indirect and direct effects of LLINs on malaria prevalence (e.g. [72]).

In spite of these limitations, we have chosen to simulate bednet interventions because it is the primary malaria intervention used in the district and was highlighted by both district and central officials as an important question. We hope our additional language allow the reader to better understand the limitations of modeling bednet interventions and interpret the results conservatively and following the precautionary principle.

(3) Accounting for biological realities/complexities of malaria transmission

As the authors note in the discussion, a full accounting of the complex transmission biology of malaria is impossible. But a few items are worth consideration. Mainly, as is well known, using SEIR type models for malaria is difficult due to concurrent infections (ie the infected class is susceptible to being multiply infected). A high proportion of falciparum infections contain multiple parasite genotypes indicating superinfection (and this has been shown to be the case in Madagascar too https://pubmed.ncbi.nlm.nih. https://pubmed.ncbi.nlm.nih.). As a result, tying gamma_A to the patent period violates our understanding of malaria transmission.

My guess is collapsing all malaria multiplicities of infection to dichotomous malaria present/absent would result in substantially underestimating FOI with downstream consequences for estimating the effect of interventions. In the literature, FOI for malaria is thus usually estimated from laborious genotyping methods (see Ivo Mueller's mFOI work from New Guinea https://pubmed.ncbi.nlm.nih.) or from cohorts with e.g., follow-up of infections cleared by treatment. My intuition is that ignoring multiple infections blunts the effectiveness of spatial targeting: Individuals with a high risk of infection get repeatedly infected, but these additional infection events are uncounted as they are unobserved during the patent period. As a result FOI is even higher than estimated in high FOI areas, whereas this undercounting is less strong in low FOI areas where the probability of multiple infection is lower. One could consider various approaches to account for this, but it seems to be a limitation for the current manuscript that warrants attention.

This is an excellent point and certainly a limitation of our current model. We have attempted to account for this in our current modeling framework by allowing for the re-infection of subclinical cases, whereby they join the Exposed compartment again (Eqs. 2,4). Given potential existing immunity from a current infection, the rate at which this occurs is scaled by the parameter *c*. This partly resolves the issue of defining a single γ_A based on the patent period, by introducing a mechanism for co-transmission of malaria genotypes. We also no longer include FOI as a parameter in the model to reduce confusion, but rather link vector and host populations via the epidemiological inoculation rate (EIR). Differing susceptibilities and compartment transition options are then accounted for in the host equations of the model, including the new potential for re-infection of sub-clinical cases.

In addition, we have added this as a limitation to the model (line 850):

> Similarly, our ability to account for multiple co-occuring malaria genotypes, as has been seen elsewhere in Madagascar [86], was limited by computational resource constraints. Omitting this co-circulation may underestimate the malaria burden in ares with high forces of infection, potentially lowering the effectiveness of Incidence-based Targeting in our model. Our inclusion of a pathway for sub-clinical cases to become re-infected partly accounts for this, but does not allow for the tracking of superinfections and its impact on malaria dynamics[87].

Minor comments by section:

Abstract and author summary:

- The third sentence of the abstract reads as a long list of limitations for targeted interventions. This may not be necessary here as not all limitations listed are relevant to the

context considered in the manuscript (e.g., non-endemic settings) and it takes away space from building the narrative

We have removed this sentence and replaced it with another to support the need for this study (line 24-30):

> However, evidence on the effectiveness of spatially-targeted interventions is mixed, depending on both the setting and intervention. In addition, the potential role of community health programs in implementing spatial targeting is underexplored, with models focusing on coarser spatial scales than those needed to guide programmatic activities

- By definition a complete ACD program would catch all cases?

Yes, it would eventually catch all cases, but not until the end of the first time step following transition to the infected compartment. This means that an infected individual could infect a mosquito in the one day period before they are treated, so transmission would not be fully disrupted.

- Could the authors define the equally distributed scenario? Is this un-targeted? If so the reader may benefit from further clarity here (i.e., stating seomthing along the lines of 'We compared X scenarios, one with no targeting and then others with targeting by XYZ...'). Later in the manuscript (e.g., line 603) this is confusing as well. Is "equally distributed targeting" the absence of targeting? Perhaps "non-targeted" is clearer

This initial language was unclear, thank you for flagging it. We now use “non-targeted” to refer to this scenario and have updated language in the abstract (and throughout the manuscript) as suggested:

> We compared five scenarios, one with no spatial targeting and others targeting communities (i) nearest clinics, (ii) furthest from clinics, (iii) with highest incidence, and (iv) that are spatially central.

Introduction:

- Refs 6 and 7 don't support the sentence as written. The Carlson et al 2023 piece is a short opinion piece without any data and the Filho et al Malaria Journal article only shows some coarse trends in national incidence stats and mean annual temp for a handful of countries

Apologies, we had accidentally entered the incorrect Carlson et al. 2023 article via Zotero. The reference should be:
Carlson CJ, Bannon E, Mendenhall E, Newfield T, Bansal S. Rapid range shifts in African Anopheles mosquitoes over the last century. Biol Lett. 19: 20220365. doi:10.1098/rsbl.2022.0365

Thank you for reading so closely and flagging this. It would have been an embarrassing error to rectify post-publication.

We have replaced the Filho article with a more specific review article on Anopheles range shifts and updated the language following the most recent work that finds that the predicted decreases in malaria incidence based on thermal-dynamic models have not as yet come to fruition:

Lyberger K, Robinson AR, Couper L, Delwel I, Glidden C, Qian C, et al. A Scoping Review of Mosquito Vector Range Shifts: Widespread Expansions and Evidence Gaps in Climate Attribution. Global Change Biology. 2025;31: e70551. doi:10.1111/gcb.70551

- The discussion of CHWs is rather aspirational. While on paper CHWs are charged with the listed tasks, it is unclear if it actually often happens on the ground outside of a single, well supported district in Madagascar. The authors point to some success stories where CHWs were used for TB, maternal health, and pandemic response but the discussion in the intro would benefit from balance to better represent the state of the field. I suggest authors mention the numerous counter examples where a lack of funding for CHWs, infrastructure, stockouts of consummables etc result in major challenges. For example, previous work by the author group identified challenges even within controlled study supported areas (https://pmc.ncbi.nlm.nih.gov/). In the Madagascar context, stock-outs have received attention (e.g., see https://pmc.ncbi.nlm.nih.gov/). In other locales, similar challenges have been reported (e.g., https://link.springer.com/, https://link.springer.com/, https://pmc.ncbi.nlm.nih.gov/). While I agree CHWs have potential in this role, the intro should be framed accordingly, making it clear this is an aspiration rather than a current capacity for CHWs across Madagascar and similar settings.

Thank you for this comment and description of the more realistic state of community health and challenges it faces. We have added a more nuanced description of the current capacity of community health programs and taken the opportunity to advocate for more investment in them if they are to serve such a role in malaria programs.

Intro (line 101):

> Notably, the success of these interventions depends on the strength of the community health programs, particularly the existence of reliable pharmaceutical stocks, financial support, and appropriate training and supervision for CHWs undertaking additional responsibilities [29,30].

Discussion (line 885):

> Our study also highlights the potential of CHWs to implement community-based interventions. However, past work in Madagascar has found the success of community malaria interventions, particularly ACD, is highly dependent on a strengthened logistics chain to prevent stock disruptions and financial support of CHWs [91,92]. Implementing community-based, spatially-targeted interventions will therefore require investment in community health programs to ensure CHWs have the additional resources needed to successfully to serve in these roles.

Methods:

There are limitations of using the selected district (Ifanadiana) to guide general policy as it is likely not representative of the 113+ other districts in Madagascar. For example, the two decades of (commendable) health system strengthening activities likely make the passive case detection and treatment rates different in the district than in other districts (with an effect on parameter tau_s*). Base treatment rates are quite high in the district (30-55%) [Lines 397-

399]. In a nearby district, Mananjary, we saw treatment rates were much lower (less than 5%) (https://www.science.org/doi/)

Thank you for this comment. Indeed, simulating malaria interventions in a district where the interventions already in place are at uniquely high rates may not accurately represent the impact of these interventions were they to be applied in other districts. We have added language describing this as a current limitation of this study (line 864):

> In addition, focusing on a district benefiting from a long-term intervention may limit the generalizability of our results to other districts, where existing treatment rates are much lower than in Ifanadiana. Small increases in treatment as these low levels could have much larger impacts in these other districts.

- Is waning for symptomatic cases (gamma_P) tied to the known half-lives / clearance rates of the drugs used? I wasn't sure why this parameter is estimated from the data (if I am reading Table S1 correctly), when the time duration of clinical efficacy of the drugs is known

It had initially been a fit parameter as the model was having issues accurately reproducing the data when fixing it, however, the addition of co-infection has allowed us to fix this parameter to 1/25 (the inverse of the prophylactic protective period), following Griffin et al. (2010). This is now noted in Table S1.

- Others have explored adding a longer lasting antimalarial (such as SP or DP) to ACD. The framework here seems well suited to exploring the potential benefits of interventions that use drugs with different durations of protection and is perhaps worth exploring

This is a very interesting point, thank you for bringing it up. The current manuscript already investigates quite a few different combinations of interventions and targeting strategies and we are hesitant to add another, particularly given it has not been highlighted by actors in the field as a current strategy being considered in Madagascar. However, we have added it as future work (line 870) and will bring this up in discussions with the national malaria control program (PNLP) if this is something they would like us to potentially add to the web application.

> We explored three interventions currently being used in Madagascar, but future work could apply a similar framework to more recent interventions, particularly pharmaceutical interventions such as vaccination (e.g. [87]) or seasonal chemoprevention (e.g. [88]), to guide targeting strategies.

- The "m" parameter does not appear to be defined in the initial model definition section and as this is often used as the number of mosquitoes in malaria models it created momentary confusion, for me at least, until defined later as the mobility matrix. Could the authors flag this in the first model description (i.e., Eqns 7 and 12)?

We have restructured the model structure section of the Methods so that the mobility models and environmental forcing are introduced earlier to avoid this confusion,as noted above. We also now provide a longer model description (lines 155):

> We developed a dynamic model of host and vector dynamics that modeled patch-specific (I.e. fokontany-level) transition rates on a daily timestep from January 1 2006 - December 31 2021. The model included host movement among patches defined by a modeled mobility matrix *m* derived from distance networks that represented the individual probability of moving from each patch i to patch j (m_ij). Vector dynamics included environmental forcing through the inclusion of patch-specific daily

> temperature, rainfall, and rice field surface water dynamics. Finally, field-based baseline values of three interventions (treatment rates, long-lasting insecticide net (LLIN) coverage, and indoor residual spraying (IRS coverage) were included in the model. Hypothetical scenarios then explored increasing these interventions follow different targeting strategies.

- Important to note that the Mordecai 2013 thermal response curves should be used with caution. It remains unclear how data from lab reared mosquitoes held at constant temperatures translate to the real world where mosquitoes can navigate complex landscapes to buffer against high temperatures (e.g., they can hide in the shade or in wells on hot days). Additionally, the Mordecai 2013 study used mosquito species that are not present in Madagascar (e.g., biting rate as a function of time is inferred for Anopheles pseudopunctipennis, a species only found in the Americas) or used lineages of An. gambiae from Lagos, Nigeria that are unlikely to represent those in

This is an important limitation of the model and we have added it to the methods (line 249):

> Temperature-dependent traits were estimated following either a Brier or quadratic function of daily temperature following Mordecai et al. [37]. The use of thermal performance curves allowed us to model temperature-dependent dynamics in multiple mosquito life stages, rather than a single reproductive number value, but did introduce further uncertainty and assumptions into the model. Specifically, we only included a mean daily temperature, ignoring diurnal fluctuations in temperature, which can be important towards thermal minima and maxima [1], and these functional curves were not made specifically for Anopheles species sourced from Madagascar, who may have different thermal adaptations than those used to estimate the thermal performance curves.

Data Collection:

- The (large) scale of under-ascertainment and thus the scale of adjustment needed for case rates should be mentioned. Per the authors' 2023 Sci Rep paper, "case notifications are capturing on average 26.5% of symptomatic malaria cases in the district". Given this level of under-ascertainment, there is likely considerable uncertainty in true case rates even after corrections are applied and the reader should be made aware of this limitation - especially as these 'corrected' case rates are referred to as 'observed' cases later in the manuscript it seems

We agree with the reviewer and have added this as a note in the methods and limitation to the discussion (line 848):

> Our field data was further biased by our use of the ZERO-G adjustment to reduce the known geographical and financial barriers to care in the district [30]. The ZERO-G method is itself a statistical model that minimizes differences in incidence rates across financial interventions and levels of geographic isolation, with accompanying uncertainty in the final incidence estimates. This uncertainty propagates to the absolute numbers of cases averted in particularly, however, given the large qualitative differences between interventions, we do not believe this alters the primary conclusions of this study.

- For clarity: "Targeted" intervention brings up connotations of "supplemental" intervention (e.g., chemoprophylaxis, vaccination or something new and fancy) that is layered on top of broad coverage of base/routine interventions (e.g., bednets) for a specific targeted population

(e.g., individuals with an occupation that predicts higher exposure risk). Targeted seems to be used in a different, and perhaps unexpected way here, where routine interventions (e.g., bednets, spraying) are restricted in some scenarios. I'm not sure how this would work in practice - future mass bednet distribution campaigns which would have by default aimed at 100% coverage, instead forego distribution in some areas I guess? The manuscript would benefit from clarity here and given the point above about nets confounding the parameterization of the model it is likely more useful to focus on other intervention types

We have added additional language to clarify that these interventions are spatially targeted and would be implemented in addition to baseline conditions in the district (line 455):

> We explored different scenarios of spatial targeting of each intervention, whereby coverage rates of an intervention were increased from baseline levels in certain fokontany (e.g. patches) based on their spatial characteristics of historical disease incidence. These interventions represented supplemental interventions on top of currently existing coverage rates in the district and did not reduce an intervention's coverage in fokontany relative to baseline.

Author contributions:

- I have a few concerns re authorship as currently listed. PLOS Computational Biology states the requirements for authorship include: "Substantial contributions to one or more of the following: conception or design of the work; acquisition, analysis, or interpretation of data; creation of new software used in the work; drafting or substantially revising the article" (https://journals.plos.org/). Currently, authors KEF and OR are listed as only providing "project supervision" and author EM does not appear to be mentioned in the author contributions section. I understand it is sometimes convention to add senior organization leadership as authors regardless of their scientific contribution. I empathize with the pressure to do so - and the politcal complexities - but encourage the authors to consider carefully - not to simply tweak the author contribution statement. I also encourage the editor to clarify as needed

Thank you again for your close read of the text. Through our co-authorship attribution, we try to value the work of collaborators who have directly contributed to analyses and drafting of the manuscript, but also those who have provided field-based interpretations of results, guided conceptualization of the project so that it meets the operational needs of the district and strategic priorities of the country, and have supported data systems that made this project possible. The last point is particularly important to us, as the digitization of health data has only facilitated the extraction of local data to conduct statistical analyses outside of an organization or country. However, the implementation of malaria interventions and the provision of malaria care while maintaining these systems and ensuring data quality is what enables secondary-data analysis such as this to be done. We believe collaborators who implement and manage these systems and have contributed to this scientific research project in ways that correspond to the authorship criteria listed by the reviewer and should therefore have their contribution formally recognized.

Indeed, Mr. Mitsinjoniala being left out of the contributions section was an error. As the Infectious Disease Manager at Pivot, Mr. Mitsinjoniala collaborates with the District Infectious Disease Manager to supervise data collection and malaria-related activities at the facility-level. This supervision enabled the collection of high-quality malaria case notifications needed by this project and his expertise and input informed the study conceptualization around which interventions were most relevant to the district. In addition, he served as a resource throughout the data analysis and results interpretation phases.

Dr. Finnegan's role as Science Director is a supervisory one, but she also serves as a CHW expert on this paper and directly edited sections concerning CHWs, contributing new text to the manuscript. In addition, she attended frequent meetings and presentations throughout the project to discuss preliminary results and ultimately finalize the analysis and manuscript narrative.

The integration of a research team within a HSS NGO allows us to have frequent and continuous meetings between researchers, medical teams within the NGO, and government officials. Throughout this project, we held meetings with partners from the National Malaria Control Program (PNLP) multiple times a year to discuss ongoing work, present preliminary findings, and obtain feedback on how interventions tested, hypotheses made, and results obtained fitted with their operational and strategic priorities. This included meeting with several members of the PNLP, including the director Dr. Raobela, although the director was ultimately chosen by their office to serve as the co-author representing the institution, who read and approved the final version of the manuscript.

We have updated the author contribution to reflect these efforts by our co-authors (line 838).

<u>Reviewer 2</u>

Overview

This paper presents a compartmental modeling approach to evaluate different strategies for spatially-targeted malaria interventions in an endemic setting. The model is age-structured, includes spatial variation for the malaria force of infection, and environmental forcing for the Anopheles vector life cycle. The model is calibrated to real-world data from a district in Madagascar and evaluated three malaria control interventions under five spatial targeting scenarios. Overall, the authors find limited evidence for spatial targeting reducing disease more than a baseline strategy of equally distributing interventions, though implementing interventions to be spatially central was slightly less impactful in reducing cases but was more resource efficient.

The question addressed in this paper is valuable for advancing malaria control efforts. Strengths of this paper include the connections to real-world data and testing multiple different interventions to show that equally distributed interventions are consistently better than spatially localized ones.

However, in the current form, I have two main concerns and a series of minor issues about this paper that require addressing. In brief, my main concerns are rooted in the current form of
the Anopheles compartmental model and why the interventions differentially impact severe vs non-severe cases.

Major comments

1. I appreciate the effort to be environmentally-grounded and precise in the Anopheles compartmental model, but I feel that for being such an explicit model, the equations exclude potentially-important environmental forcings.

1. First, the authors describe how the district being modeled in this study is particularly rural. Yet, the Anopheles force of infection only includes vector transmission from a human host. Previous work has shown that the spatial patterns of malaria in Madagascar are impacted by non-human hosts. Moreover, increasing the spatial variability in the vector model may impact the conclusions of the paper that spatial targeting does not impact malaria incidence as much as equally-distributed interventions. As such, I feel that removal of non-human hosts is not a fair

simplification and should be reconsidered for this study. While an explicit model of non-human primates is likely unnecessary, adding an environmental forcing where the number of infected mosquitoes depends on an environmental variable that is a proxy for zoonotic spillovers would be helpful.

To our knowledge, there is very little risk of zoonotic spillover of malaria to human hosts in Ifanadiana district, where malaria is primarily the human-specific *P. falciparum*. While Ranomafana National Park is located within Ifanadiana district and serves as habitat to many lemur species, they are not known to be hosts of human malaria and the park itself is only in one small area of the district. We therefore expect the risk of zoonotic spillover to be very minimal and have chosen not to include it in the model.

Anopheles mosquitoes in Madagascar will feed on non-human hosts, particular bovine and porcine hosts (see Tedrow et al. 2019), however our model assumed that the availability of blood meals was not a limiting factor in mosquito population growth, but driven primarily by temperature, rainfall, and density-dependent mortality. In addition, these domestic animals' distributions across the district is fairly homogeneous and would not introduce additional spatial variance not already included via human population densities. We now note this explicitly in the methods (line 232):

> Mosquito larvae (L_V) were born as a function of the total adult population N_V and temperature-dependent fecundity rates, b(T), assuming blood meal availability was not a limiting factor.

2. The authors include an environmental forcing for rainfall-induced flushing of the Anopheles habitat. This function assumes that rainfall directly varies with Anopheles mortality. This neglects that small amounts of rainfall, in particular prior to the monsoon season, may provide the necessary water for Anopheles to survive. In other words, there is a non-monotonic relationship between rainfall and Anopheles presence. This effect has been well-documented previously where water hoarding during drought is associated with an increase in mosquito presence rather than a decrease as may be expected during drought. It seems that the parameters in Eqns. 15 and 16 are fit in the model's ABC calibration process. If this is the case, it would be valuable to report the posterior values of these parameters in the supplemental. In particular, I am curious what is the interplay between Eqn. 15 and Eqn. 16. It seems unlikely that the amount of larvae ever get to a number so high, especially with rainfall-dependent flushing from Eqn. 15, that there is any significant impact from a finite carrying capacity of flooded rice fields (Eqn. 16), so I wonder if these parameters can actually be calibrated.

Thank you for pointing this out. As you note, rainfall is only included directly in Eq. 14, where it impacts larval mortality via flushing. However, it is also included indirectly via the rice field flooding term in Equation 15, which closely follows rainfall dynamics (Fig. 2). The parameters in Eq. 14 are fixed following Tompkins and Ermert 2013, resulting in the following flushing-induced mortality function across rainfall values:

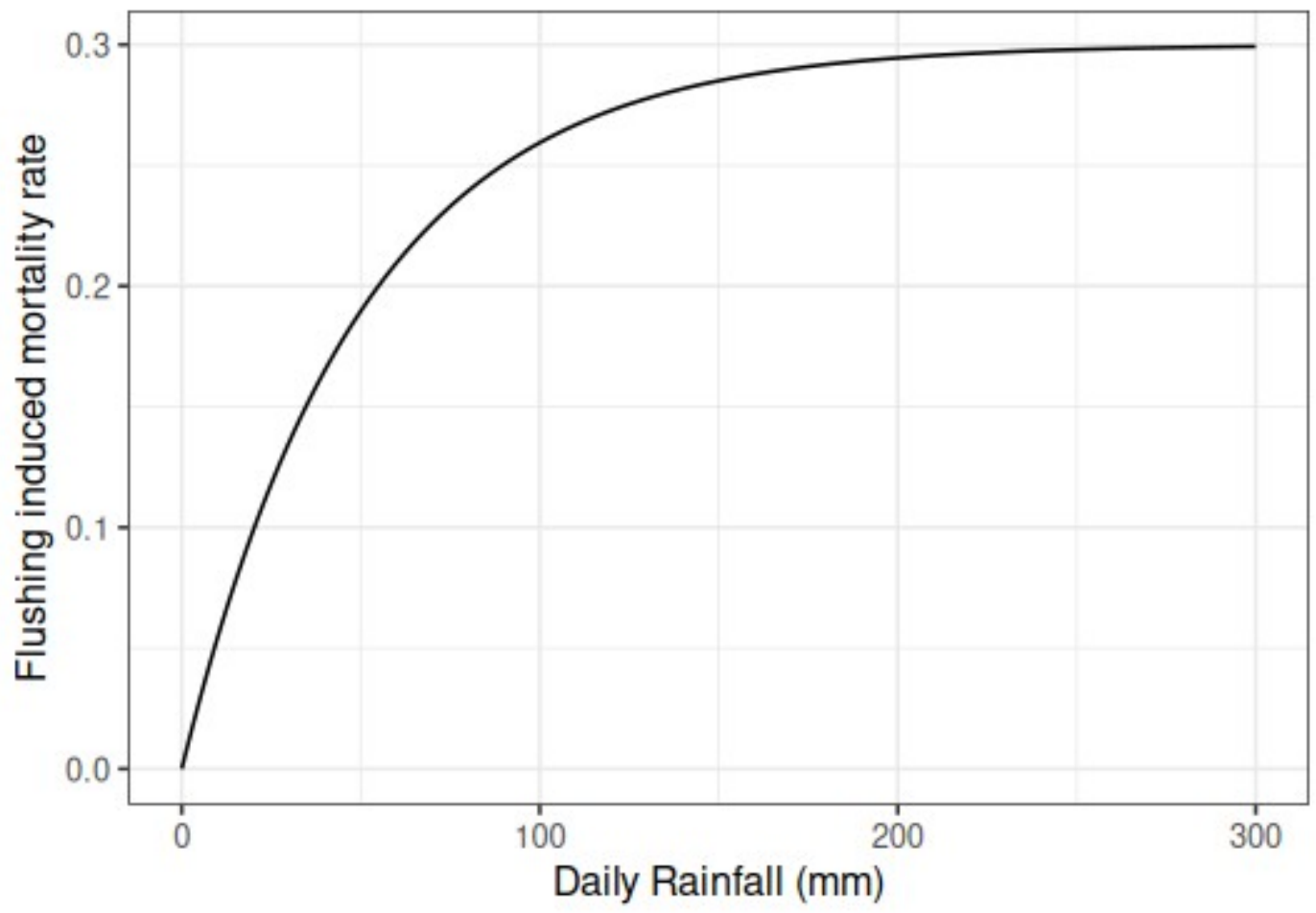

It is difficult to tease apart the influence of rainfall alone on mosquito dynamics because its seasonality is closely tied to temperature and it indirectly impacts flooding dynamics with a slight lag. However, as a counterfactual example, we explored the relationship between the abundance of larval mosquitoes, flushing-caused mortality, and density-dependent mortality with rainfall and flooding by simulating vector dynamics for several example fokontany while holding temperature constant at 22C and without any disease transmission or interventions. Fokontany were chosen to represent different climates within the district. This does reveal a unimodal relationship with rainfall caused by the combination of both mortality terms, although it is dependent on the fokontany and the relationship between rainfall and ricefield flooding within that fokontany:

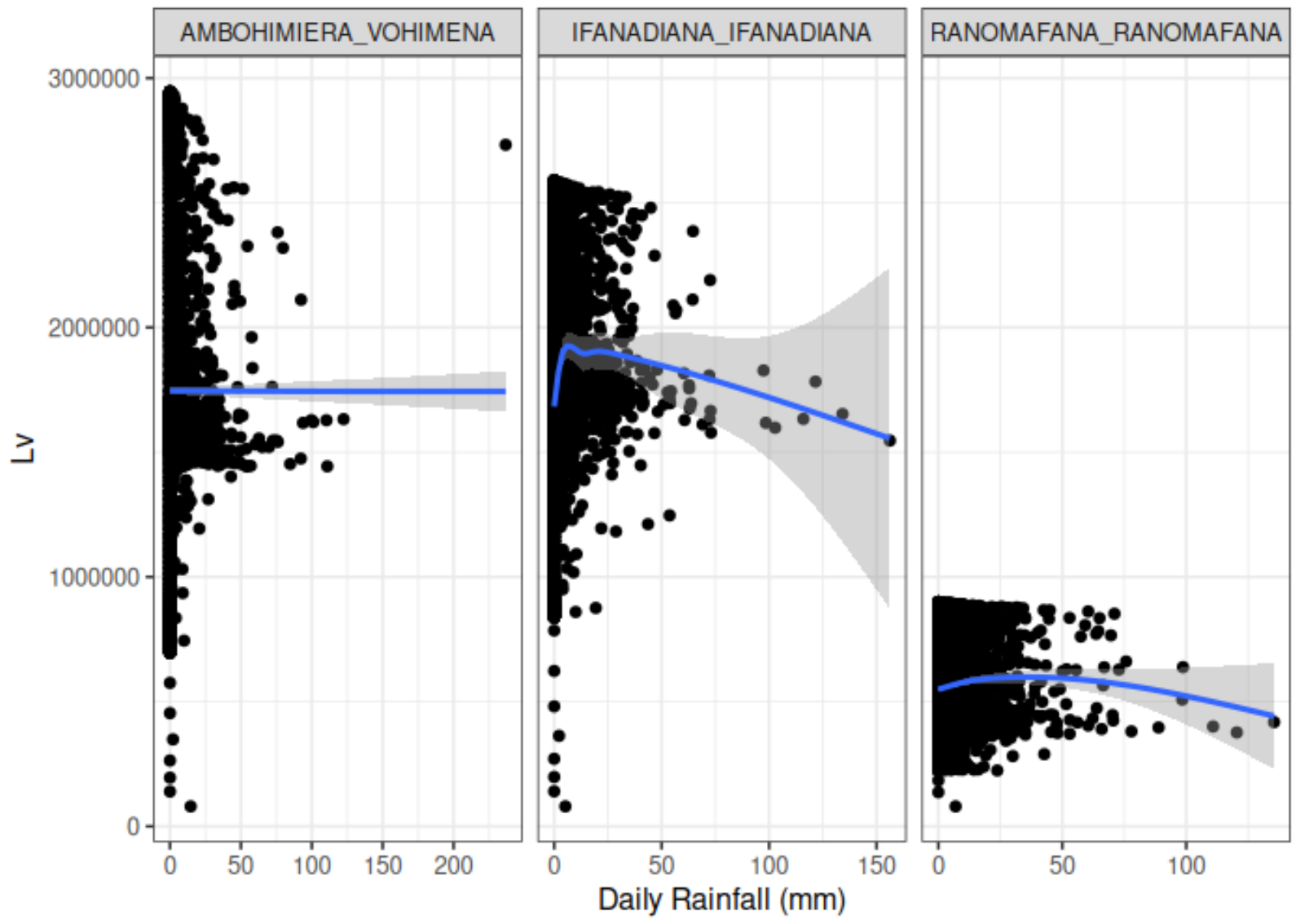

Of these equations, only one parameter is fit ,*K*, representing the carrying capacity of larval mosquitoes of 1 m^2 of flooded ricefield. The rest of the parameters are fixed based on the literature (Supp. Table 1), allowing us to estimate this parameter. We have currently chosen not to include the figures above as supplemental figures in the manuscript, to avoid the manuscript from getting too long and difficult to follow the many smaller supplemental analyses, but are happy to add this to the main text if the reviewers and editorial team think it important to do so.

2. Three interventions are considered in this study: LLIN, IRS, and ACD. LLIN and IRS impact only the mosquito biology, with LLIN impacting both the biting and mortality rates and IRS impacting only the mortality rate. The authors describe these impacts in Eqns. 20-24. However, when describing the results of these strategies, they show that

the strategies differentially avert simple vs severe malaria cases among children and adults. Moreover, the ratio of simple to severe averted differs by spatial targeting strategy. This finding is very surprising to me. In order for the interventions to differentially impact the number of simple vs severe cases, there must be some difference in how simple cases proceed to severe cases that changes based on the intervention. The intervention equations only describe impacting the Anopheles vectors, not introducing some parameters in the disease progression model. Similarly, the only parameter in the disease progression model that differs by patch is the treatment rate for asymptomatic individuals (see minor comment #2 for further thoughts on this parameter), so this effect cannot be driven by fewer mosquitoes in one area causing less progression to disease severity. No explanation for these findings are given. An explanation of these findings would be useful as well as an audit of whether they are right.

Yes, there is a very slight difference in the percentage of averted simple and severe malaria cases for the interventions that target only the mosquito vector, as shown in Table 2. You rightly point out that, given these are only acting on the vector, that should not be the case as simple cases are advancing into severe cases at the same rate under all levels of intervention. This difference is due to stochastic noise cause by the use of stochastic binomial probabilities for the transition rates of host individuals and the small number of severe cases overall. Under baseline intervention rates, each patch has on average 1 severe childhood malaria case a year (range: 0 -12), or 176 (85-230) at the district-level. Because transition rates are happening at the scale of the patch, this makes them very sensitive to the stochasticity of the transition probabilities. This is also demonstrated by the wide confidence intervals in Table 2, which show that although the mean may differ, the confidence intervals of the simple and severe cases still mostly overlap for the IRS and LLIN interventions.

This context has been added to the Results to aid the reader in their interpretation of the results (line 661):

> The other interventions performed similarly for simple and severe cases, although there was more uncertainty around severe cases averted given the lower overall numbers (Table 2). The number of severe childhood malaria cases was relatively low at the patch level, with on average one case per year. Because of this, the stochastic binomial transition rates introduced high amounts of demographic stochasticity across iterations.

Minor comments

1. In the model, symptomatic individuals transition to an asymptomatic infection. While this is correct for malaria biology, the wording of these two compartments may be confusing, especially for the infectious disease modeling community which is used to the convention that an asymptomatic phase either precedes a symptomatic phase or asymptomatic individuals never become symptomatic. The paper could benefit from either making the choice of this transition clear to the reader or relabeling the asymptomatic compartment as “subclinical” or a similar term. Additionally, this modeling choice has a critical assumption baked into it: individuals who are symptomatic and not treated/do not develop severe malaria have the total infectious period of symptomatic individuals plus asymptomatic individuals. While there is evidence to suggest that this is the case, the paper would benefit from making this assumption clear.

We appreciate this suggestion and have changed the language to refer to the previously "asymptomatic" compartment as "subclinical" throughout the manuscript.

2. The compartmental model has many quantities that are age-/patch-specific (or both). While these choices largely make sense or are justified, there is no motivation behind why the asymptomatic treatment rate differs by patch, especially when the symptomatic treatment rate does not. It would be helpful to see a justification for this assumption – either by showing that there is necessary data to parameterize this difference or via a supplemental analysis showing that the model fitting requires these to be different.

This was an error in the equation notation as these traits do vary under the targeted ACD interventions. This has been corrected (Eq. 4, Supp Table 1).

3. The force of infection (Eqn. 7) uses quantities from the vector compartmental model (the infected number of mosquitoes) prior to those quantities being introduced. Please restructure the section to not use quantities prior to their definition.

In response to this comment and others regarding the methods section, we have restructured the methods section and adjusted our language so that it is clearer to the reader. It is difficult to follow via the Track Changes, but we now introduce the host and vector equations together, describe how they are linked via the mobility matrices, introduce specific aspects of environmental forcing for certain parameters, and then provide equations for the impact of IRS and LLIN on vector rates. This ensures parameters are not introduced before they are formally defined.

4. The authors use their previously-developed approach of ZERO-G to correct the raw malaria case counts. It would be helpful to see a supplemental figure of what this procedure does to the data, and if the changes are dramatic, it would be helpful to see a supplemental analysis showing the model fits on the raw data, motivating the use of ZERO-G.

Applying the model fitting procedure to the uncorrected data would involve adjusting the ABC summary statistic to optimize on $I_h \tau_S$, rather than the change in I_h, to represent only those symptomatic cases who sought treatment and rerunning the model fitting procedure on the new data, which requires 22,500 computational hours per model for those including patch-based mobility. We have therefore opted not to re-run the model fitting procedure but do include a supplemental figure to visualize the difference between the raw and adjusted data as well as further discussion of the uncertainty this introduces (line 836):

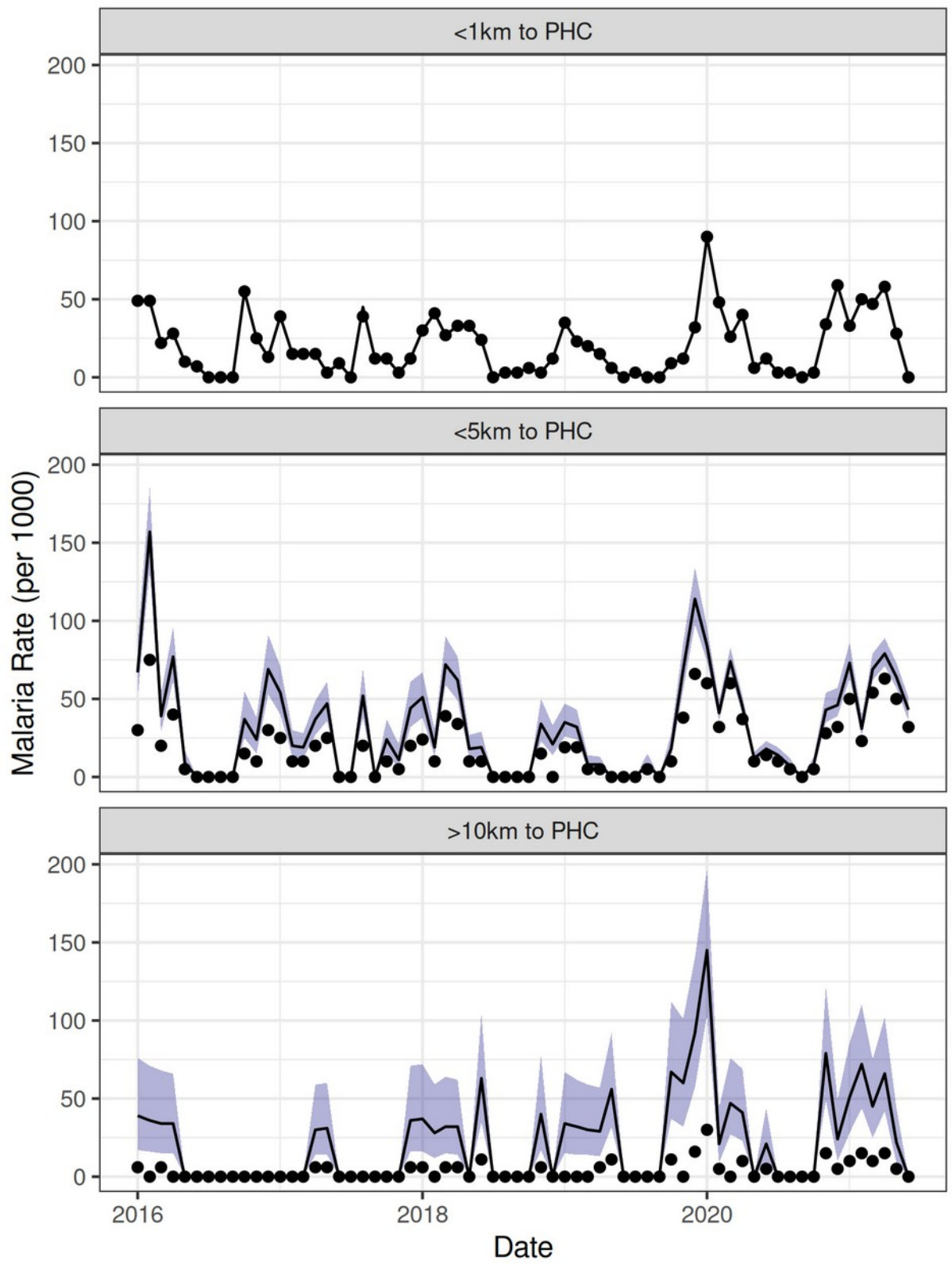

Supplemental Figure 2. Comparison of ZERO-G adjustment for a fokontany within 1 km of a primary health center (PHC), within 5 km of a PHC, and greater than 10km from a PHC within one commune. Points represent the raw malaria incidence rate, lines represent the adjusted rate, and shaded error bars represent 95% confidence intervals.

We have added the following text to the discussion (line 867):

> Our field data was further altered via the ZERO-G adjustment to reduce the known geographical and financial barriers to care in the district [32]. The ZERO-G method is itself a statistical model that minimizes differences in incidence rates across financial interventions and levels of geographic isolation. Although it aims to reduce known biases, it necessarily introduces other sources of uncertainty in the final incidence estimates, resulting in more realistic, but not perfect, data. This uncertainty propagates to the absolute numbers of cases averted in particularly, however, given the large qualitative differences between interventions, we do not believe this alters the primary conclusions of this study.

5. The radiation mobility model used in this manuscript has a degeneracy when s ij = 0. Either this quantity must also include the population of the patch i itself (in which case, this equation is double counting the probability that an individual does not move), or the authors must have had some other way of dealing with a divide by zero issue.

6. The radiation model used in this study differs from the original one presented by Simini – in this study, the population of the destination is squared. A justification of this change from the original formulation would be helpful.

We have updated how this is defined in the equations to more closely follow the original Simini study definition. However, because the probability of movement is used in multiple equations to define the movement of different sub-populations (e.g. infectious hosts in Eq. 16, susceptible hosts exposed to different EIRs while moving in Eq. 1), we include the probability of movement directly in the mobility model rather than as a separate term, as Simini does via

T_i, which represents the number of commuters. The mobility model can then be combined with any sub-population count at the source patch to estimate the number of individuals of that sub-population moving to each of the other patches j.

The mobility model is now described via equations 22 and 23 and integrated into the compartmental model via the FOI on mosquito populations (Eq. 12) and the EIR of the host population (Eq. 11). We have also tried to clarify how this model was calculated in the text (lines 341):

> For the radiation model, the probability of moving to patch j is dependent on the distance between patches dij and the population of patches p via the radiation model rad' [40,41] . Population values of neighboring patches are included via the parameter sij, which represents the total population within a distance dij of patch i, excluding patches i and j:
>
> These models were applied to each distance dataset separately. For all models, the probability of remaining in patch i ($m_{i,j=i}$) was a fit parameter, replacing the diagonal of the mobility model matrix. Each mobility model was then scaled so that the total sum of movement probabilities out of each patch i ($\sum_j m_{ij}$) equaled 1. The value m_{ij} was then used as the daily probability of moving from patch i to j for each individual in source patch i.

7. The ultimate finding of this paper is helpful, but it is surprising. The authors argue that high case rates of Ifanadiana district make hotspot targeting unlikely to be effective, but it is surprising that spatial targeting is so ineffective. Naively, I would have expected that the variance is much smaller with spatial targeting being potentially just as effective as equally-distributed strategies. The paper would benefit from a sensitivity analysis showing whether these strategies are effective if malaria rates are lower, validating the authors' hypothesis that the strategy is ineffective because rates are so high. On the other hand, one argument for spatially-targeting being ineffective is that mobility is very high: if mobility is high, people are more likely to be infected in areas where they do not live and then potentially bring the disease back to their home patch, so spatial targeting is ineffective. Likewise, the paper would benefit from a sensitivity analysis testing whether this hypothesis is plausible.

Thank you for this comment. We have chosen to conduct the mobility sensitivity analysis, as we have no data to confirm the model estimated mobility rates. However, because the study is focused on a malaria-endemic region of Madagascar where prevalence rates are high, we believe exploring the effectiveness of interventions under different malaria rates to be outside the scope of the current study. In addition, it would involve refitting the model to counterfactual data representing lower case and prevalence rates to estimate parameters in this instance, a non-trivial undertaking.

To explore differing mobility rates, we fixed the probability of remaining in the source patch while using all other mobility parameters as estimated. This resulted in three mobility levels: low (25% move), medium (50% move) and high (75%). We then replicated the comparison of interventions and targeting strategies on each of these three scenarios and compared the average number of annually averted cases:

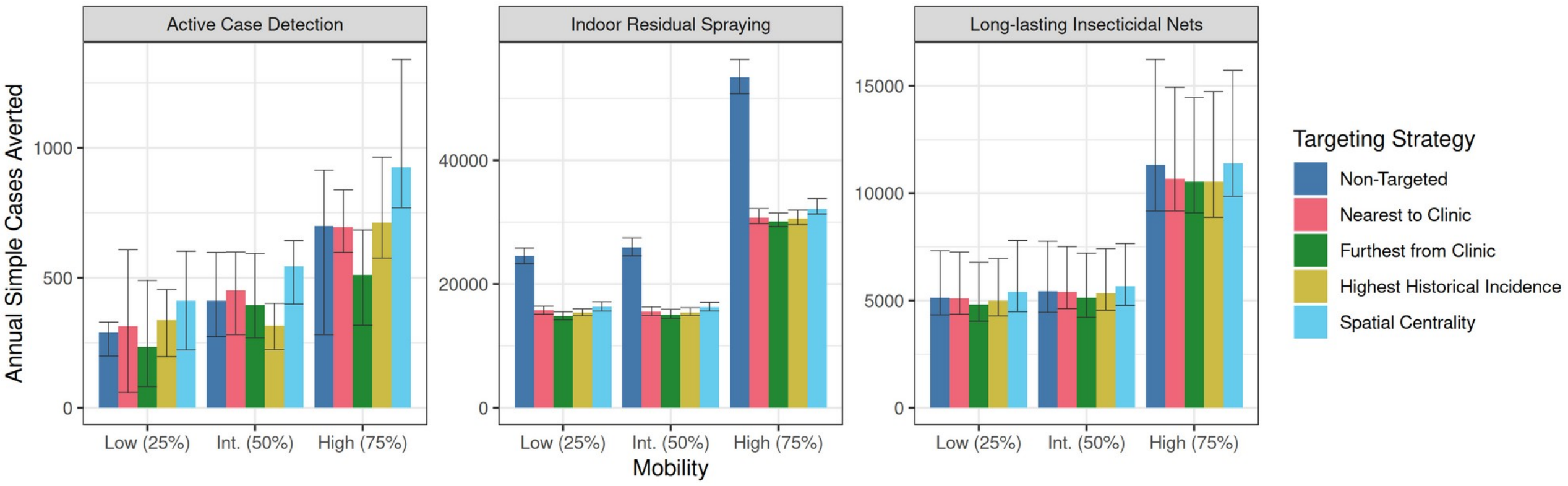

Fig. S6. Interventions were more effective at high mobility rates across all targeting scenarios.

Contrary to the proposed hypothesis, we found that increasing mobility rates increased the effectiveness of all three interventions. This agrees with one aspect of the "assembly effect", which is that interventions with sub-optimal coverage can be successful if they are highly-connected to communities with above-optimal coverage. Similarly, we found that targeting more central patches was the most effective targeting strategy, as it also increased coverage in the most highly-connected patches. Together, this provides further evidence for the benefit of using information on mobility and spatial structure in populations to guide targeted interventions.

We have added the following text to the manuscript regarding this sensitivity analysis.

Methods (line 534):

> Spatially-targeted malaria interventions can have indirect effects on disease in neighboring fokontany via "community-level" effects[55]. The direction of these effects are dependent on the relative difference in disease burden between patches and their connectivity[56]. We therefore conducted a sensitivity analysis to explore the relative effectiveness of different targeting strategies under different population mobility rates. Using the best fit mobility model, we fixed the probability of remaining in the source patch (p_m) while using all other mobility parameters as estimated. This resulted in three mobility levels: low (25% move), medium (50% move) and high (75% move). We then simulated the same combination of interventions and targeting strategies on each of these three scenarios and compared the average number of annually averted cases across 19 iterations.

Results (line 676):

> When considering differing mobility rates, we found that intervention effectiveness increased with increasing mobility rates for all targeting scenarios (Supp. Fig. 6).

Discussion (line 799):

> However, it is notable that the best performing spatially targeted intervention was not based on case incidence or prevalence, as is often used in hotspot targeting, but on the underlying spatial structure of the transport network by targeting the most central patches. This is in agreement with our finding that interventions are more successful for more mobile populations. Tun et al. [56] found that above-optimal MDA coverage in highly connected patches can counteract sub-optimal coverage in other patches using mathematical models, leading to a net success. Our results agree with this and suggest that incorporating spatial patterns in population density and movement when allocating resources could be more effective than guiding interventions using historical disease case rates, if spatial targeting is to be used.

8. Line 67: “arteminisinin” should be “artemisinin”.

Thank you for your close eye. We have corrected this.
