# Supplementary material for: Leveraging spatial structure to design spatially-targeted malaria interventions at the community-scale
Source: PLoS Comput Biol. 2026 Oct 15;22(10):e1014857. doi: 10.1371/journal.pcbi.1014857 (PMC13690273; doi:10.1371/journal.pcbi.1014857)
Supplement: Supplementary file 3 [file pcbi.1014857.s002.docx]

**Leveraging spatial structure to design spatially-targeted malaria interventions at the community-scale**

**Overview**

This paper presents a compartmental modeling approach to evaluate different strategies for spatially-targeted malaria interventions in an endemic setting. The model is age-structured, includes spatial variation for the malaria force of infection, and environmental forcing for the *Anopheles* vector life cycle. The model is calibrated to real-world data from a district in Madagascar and evaluated three malaria control interventions under five spatial targeting scenarios. Overall, the authors find limited evidence for spatial targeting reducing disease more than a baseline strategy of equally distributing interventions, though implementing interventions to be spatially central was slightly less impactful in reducing cases but was more resource efficient.

The question addressed in this paper is valuable for advancing malaria control efforts. Strengths of this paper include the connections to real-world data and testing multiple different interventions to show that equally distributed interventions are consistently better than spatially localized ones.

However, in the current form, I have two main concerns and a series of minor issues about this paper that require addressing. In brief, my main concerns are rooted in the current form of the *Anopheles* compartmental model and why the interventions differentially impact severe vs non-severe cases.

**Major comments**

1. I appreciate the effort to be environmentally-grounded and precise in the *Anopheles* compartmental model, but I feel that for being such an explicit model, the equations exclude potentially-important environmental forcings.
   1. First, the authors describe how the district being modeled in this study is particularly rural. Yet, the *Anopheles* force of infection only includes vector transmission from a human host. Previous work has shown that the spatial patterns of malaria in Madagascar are impacted by non-human hosts. Moreover, increasing the spatial variability in the vector model may impact the conclusions of the paper that spatial targeting does not impact malaria incidence as much as equally-distributed interventions. As such, I feel that removal of non-human hosts is not a fair simplification and should be reconsidered for this study. While an explicit model of non-human primates is likely unnecessary, adding an environmental forcing where the number of infected mosquitoes depends on an environmental variable that is a proxy for zoonotic spillovers would be helpful.
   2. The authors include an environmental forcing for rainfall-induced flushing of the *Anopheles* habitat. This function assumes that rainfall directly varies with *Anopheles* mortality. This neglects that small amounts of rainfall, in particular prior to the monsoon season, may provide the necessary water for *Anopheles* to survive. In other words, there is a non-monotonic relationship between rainfall and *Anopheles* presence. This effect has been well-documented previously where water hoarding during drought is associated with an increase in mosquito presence rather than a decrease as may be expected during drought. It seems that the parameters in Eqns. 15 and 16 are fit in the model’s ABC calibration process. If this is the case, it would be valuable to report the posterior values of these parameters in the supplemental. In particular, I am curious what is the interplay between Eqn. 15 and Eqn. 16. It seems unlikely that the amount of larvae ever get to a number so high, especially with rainfall-dependent flushing from Eqn. 15, that there is any significant impact from a finite carrying capacity of flooded rice fields (Eqn. 16), so I wonder if these parameters can actually be calibrated.
2. Three interventions are considered in this study: LLIN, IRS, and ACD. LLIN and IRS impact only the mosquito biology, with LLIN impacting both the biting and mortality rates and IRS impacting only the mortality rate. The authors describe these impacts in Eqns. 20-24. However, when describing the results of these strategies, they show that the strategies differentially avert simple vs severe malaria cases among children and adults. Moreover, the ratio of simple to severe averted differs by spatial targeting strategy. This finding is very surprising to me. In order for the interventions to differentially impact the number of simple vs severe cases, there must be some difference in how simple cases proceed to severe cases that changes based on the intervention. The intervention equations only describe impacting the *Anopheles* vectors, not introducing some parameters in the disease progression model. Similarly, the only parameter in the disease progression model that differs by patch is the treatment rate for asymptomatic individuals (see minor comment #2 for further thoughts on this parameter), so this effect cannot be driven by fewer mosquitoes in one area causing less progression to disease severity. No explanation for these findings are given. An explanation of these findings would be useful as well as an audit of whether they are right.

**Minor comments**

1. In the model, symptomatic individuals transition to an asymptomatic infection. While this is correct for malaria biology, the wording of these two compartments may be confusing, especially for the infectious disease modeling community which is used to the convention that an asymptomatic phase either precedes a symptomatic phase or asymptomatic individuals never become symptomatic. The paper could benefit from either making the choice of this transition clear to the reader or relabeling the asymptomatic compartment as “subclinical” or a similar term. Additionally, this modeling choice has a critical assumption baked into it: individuals who are symptomatic and not treated/do not develop severe malaria have the total infectious period of symptomatic individuals *plus* asymptomatic individuals. While there is evidence to suggest that this is the case, the paper would benefit from making this assumption clear.
2. The compartmental model has many quantities that are age-/patch-specific (or both). While these choices largely make sense or are justified, there is no motivation behind why the asymptomatic treatment rate differs by patch, especially when the symptomatic treatment rate does not. It would be helpful to see a justification for this assumption – either by showing that there is necessary data to parameterize this difference or via a supplemental analysis showing that the model fitting requires these to be different.
3. The force of infection (Eqn. 7) uses quantities from the vector compartmental model (the infected number of mosquitoes) prior to those quantities being introduced. Please restructure the section to not use quantities prior to their definition.
4. The authors use their previously-developed approach of ZERO-G to correct the raw malaria case counts. It would be helpful to see a supplemental figure of what this procedure does to the data, and if the changes are dramatic, it would be helpful to see a supplemental analysis showing the model fits on the raw data, motivating the use of ZERO-G.
5. The radiation mobility model used in this manuscript has a degeneracy when *s_ij_ =* 0. Either this quantity must also include the population of the patch *i* itself (in which case, this equation is double counting the probability that an individual does not move), or the authors must have had some other way of dealing with a divide by zero issue.
6. The radiation model used in this study differs from the original one presented by Simini – in this study, the population of the destination is squared. A justification of this change from the original formulation would be helpful.
7. The ultimate finding of this paper is helpful, but it is surprising. The authors argue that high case rates of Ifanadiana district make hotspot targeting unlikely to be effective, but it is surprising that spatial targeting is so ineffective. Naively, I would have expected that the variance is much smaller with spatial targeting being potentially just as effective as equally-distributed strategies. The paper would benefit from a sensitivity analysis showing whether these strategies are effective if malaria rates are lower, validating the authors’ hypothesis that the strategy is ineffective because rates are so high. On the other hand, one argument for spatially-targeting being ineffective is that mobility is very high: if mobility is high, people are more likely to be infected in areas where they do not live and then potentially bring the disease back to their home patch, so spatial targeting is ineffective. Likewise, the paper would benefit from a sensitivity analysis testing whether this hypothesis is plausible.
8. Line 67: “arteminisinin” should be “artemisinin”.
