# Supplementary material for: Leveraging spatial structure to design spatially-targeted malaria interventions at the community-scale
Source: PLoS Comput Biol. 2026 Oct 15;22(10):e1014857. doi: 10.1371/journal.pcbi.1014857 (PMC13690273; doi:10.1371/journal.pcbi.1014857)
Supplement: Supplementary file 4 [file pcbi.1014857.s005.docx]

**Reviewer 1:**

**Overall comments:**

**I thank the authors for the discussion around the previous comments regarding the mobility analyses and the current manuscript version is improved. I remain concerned however that mobility is assumed and that mobility parameters may be capturing latent variation from some other source, with implications for interpretation.**

We agree this is an appropriately conservative interpretation of our results and highlight this throughout the manuscript. We have changed our language to refer to spatial structure, rather than mobility, except when discussed in the modeling methods themselves, as it is implemented via the movement of individuals. We also include the following caveat about our results and their interpretation (line 715):

*Data on inter-fokontany movement is not available for Ifanadiana district, and we were therefore unable to validate whether this term accurately represented true human movement or whether its inclusion in the model served more as a latent variable of spatial variation that represented spatial covariance in EIR and FOI not explained by the environmental forcing terms. Our finding that different models performed similarly suggests that it is the inclusion of any fit spatial structure, and associated unmeasured covariance, that improved model performance, rather than a specific model formulation, provided the parameters are flexible enough to fit the underlying spatial covariance. Overall, the general conclusion is the same: spatial structure in host and vector dynamics should be considered when modeling malaria at the community scale.*

**Figure S6 seems an important and interesting result. For Figure 3 on the other hand, no mobility seems to be an unfair comparison. Coarsely, allowing mobility allows more parameters and allows 'smoothing' differences between observed/adjusted cases and expected cases by 'borrowing' cases from other patches which will always result in a better fit?**

**Additionally, point taken that the distance matrices are all likely to be highly correlated but it is a bit curious that results in Figure 3 were so similar across the different mobility models that had quite different assumptions? This might suggest the model structure rather than human mobility networks per se are forcing the results?**

We have added additional discussion to the mobility sensitivity analysis results (line 762):

*In addition, source-sink dynamics of malaria have been observed at the national scale in Madagascar [76], and the increased effectiveness of interventions at higher mobility rates may be related to similar dynamics at a fine scale, allowing interventions to impact neighboring regions at higher population mobility.*

In addition, we describe in more detail our findings regarding the importance of including spatial structure to address unmeasured spatial covariance, and that the exact model to do so is relatively flexible (line 740):

*Our finding that different models performed similarly suggests that it is the inclusion of any fit spatial structure, and associated unmeasured covariance, that improved model performance, rather than a specific model formulation, provided the parameters are flexible enough to fit the underlying spatial covariance.*

**Minor comments by section:**

**Abstract and author summary:**

**- The text "although a “perfect” ACD intervention could avert 100% of severe malaria cases" is still coming across unclear/circular. Perhaps rephrasing would benefit the reader?**

Yes, we see this circularity. We have updated the text to read:

*No combination of interventions was able to eliminate malaria in the district, although detecting 100% of cases through ACD could avert 100% of severe malaria cases.*

**- I suggest specifying "supplemental" or "reinforced" (or some other adjective) for the LLIN distributions - distinguishing from the routine LLIN distributions seems important**

We agree and have updated this language to refer to it as “supplemental coverage” throughout the manuscript.

**Introduction:**

**- The text "The model includes fine resolution spatial data of human movement" is misleading if there are no observations of human movement. See also the discussion [Line 668] where "Notably, the model was based on empirical environmental data and distance networks" seems to imply the presence of empirical human movement data. Likewise Line 696 states "We found that including realistic spatial structure in human mobility", but tht seems unjustified justified without comparison to real mobility data?**

We have adjusted our language to better represent our use of a transport network and not empirical data on human mobility:

*line 103: Here, we develop a spatially-explicit dynamic model of malaria that includes spatial structure via a transport network and environmental forcing...*

*line 106: The model includes fine resolution spatial structure via a transport network based on…*

*line 297: Spatial structure was included via modeled daily movements between patches estimated from field-based distance networks.*

*line 668: Notably, the model was based on empirical environmental data and used transport networks to inform spatial structure.*

*line 696: We found that including realistic spatial structure via a distance matrix increased model performance*

We do, however, continue to refer to mobility models and host movement with regards to model structure, as this is the way it is explicitly written in the model.

**Methods:**

**- Returning to the CHW discussion, the usefulness of statements such as "Each fokontany has a community health site staffed by CHWs who are able to treat malaria cases in children under 5 years old and pregnant women [33]." is unclear. Does this happen regularly? I understand the aspiration is for this to happen but do CHWs actually have tests and drugs most of the time? And if so, is it likely happening in many districts in Madagascar or a unique feature of Ifanadiana?**

The goal of this sentence is to inform the reader of the CHW policies in Madagascar and specifically in Ifanadiana District (the “Study Setting”), i.e how many CHWs there are per fokontany, their role in treating malaria, etc.. As the reviewer notes, patients’ use of CHWs can differ by region. In Ifanadiana, which benefits from a community-health intervention, CHW per capita all-cause consultations for children under 5 years old range from 1-2 per year (Garchitorena et al. 2026, *PlosMed* [*https://journals.plos.org/plosmedicine/article?id=10.1371/journal.pmed.1004549*](https://journals.plos.org/plosmedicine/article?id=10.1371/journal.pmed.1004549)*).* Ifandiana is not an exception, a recenty survey showed that CHW involvement in malaria care was near universal (98.5%) across four regions of Madagascar (Andriamasy et al. 2026, *BMJ GH, https://doi.org/10.1136/bmjgh-2025-023393*). Although this survey relied on CHW responses, the nearby district of Farafangana also reported that 76.1% of individuals had visited a CHW in the prior year, the majority for febrile illness, suggesting they are being used in other districts as well (Sayre et al. 2021, *Mal Journal,* [*https://doi.org/10.1186/s12936-021-03956-z*](https://doi.org/10.1186/s12936-021-03956-z)*). W*hile there are certainly disruptions to care due to supply chain issues, absenteeism, geographic barriers, etc., CHWs in Madagascar are a resource that the population uses for malaria care. We have added some additional nuance to the text to this effect (line 131):

*While disruptions to the supply chain and staffing can impact the availability of malaria treatment and care from CHWs, the all-cause consultation rate at CHWs in Ifanadiana for children under 5 ranges from 1 -2 per capita consults per year [1], suggesting that they are serving the population in spite of these disruptions.*

**- How does the bednet distribution intervention (presumably kept at residence) work in the context of movement? Highly mobile populations need to carry their bednets with them or faced reduced effectiveness? Perhaps this warrants comment to link the spatial mobility and interventions more concretely**

**- Could the discussion around bednet interventions disaggregate the intervention into more specific strategies? Some of the discussion in response to previous reviewer comments gets at this but some nuance missing in main text re simply passing out more bednets vs increasing usage rates vs quality etc?**

We have added additional discussion around specific strategies surrounding LLIN interventions, and not just distribution campaigns (line 774):

*Our results should not be interpreted to mean that LLIN distribution campaigns should be decreased or that they should no longer strive for universal coverage, as these campaigns have averted over 400 million cases [77]. Rather, LLIN-related interventions should focus on specific strategies to increase their effectiveness. For example, in Ifanadiana district, the IHOPE survey has found household coverage rates of over 90%, however fewer than 50% of households meet the WHO recommendation of one net per two people. Increased returns on LLIN-based interventions may therefore be more likely by focusing on increasing use rates themselves through sensibilizations or ensuring net quality, rather than simply increasing the household-level coverage of distribution campaigns, which can have limited returns at very high coverage values [78]. A highly-mobile population further complicates a LLIN intervention, as individuals are not likely to travel with their own LLIN, resulting in individuals losing LLIN protection or households exceeding the WHO recommendation. In instances where travel is repeated, such as to a farm or rice field, a second net could be installed in the temporary residence and LLIN distribution campaigns could take this into consideration during the census stage.*

**Optional sensitivity analyses:**

**- Could one simply inflate infection rate in high incidence localities by a range of values and see what happens if you are undercounting infections by say X%? I believe this is equivalent to making the ZERO-G adjustment, but adding a 'heavier' adjustment in high incidence districts if going for number of infections rather than just positive/negative**

It was not fully clear to us what this requested sensitivity analysis would be assessing. If it is meant to test how robust the results are to any bias introduced by the ZERO-G adjustment, it would involve replicating the full model fitting and intervention simulation process on a completely new dataset, which is big computational undertaking. It is then not clear how we would assess which dataset is more “correct” besides the assessments already undertaken through the ZERO-G method (comparing to malaria seroprevalence, attempting to control for known biases in data due to differing reimbursement policies, etc.). Further, the raw incidence rates are generally highest in those fokontany that contain or are neighboring a fokontany that contains a health facility, due to the close geographic proximity. In our opinion, further inflating these incidence rates would worsen the existing geographic bias in the data. This is further supported by data from the complementary IHOPE survey which finds higher health-seeking rates in those communities closest to health facilities (see Evans et al. 2023 <https://doi.org/10.1038/s41598-023-48390-0>). Perhaps a better test of the applicability to another dataset would be to replicate the model fitting and intervention simulation process for a completely different district in Madagascar or another country entirely, which we believe to be outside the scope of our current work. We have therefore chosen not to conduct this sensitivity analysis but do mention in the discussion that replicating this study in other malaria endemic contexts would further test how generalizable the results are (line 840):

*Applying this method to data from other malaria endemic contexts could further test the robustness of these conclusions.*

**- Also not required but could be interesting to see a further sensitivity analysis where treatment rates were lower? Could aid in interest for a broader audience working in areas without Ifanadiana's health system strengthening projects?**

We agree that focusing on Ifanadiana District, where we have the most data but which is very unique, could make our results less applicable to other health districts in Madagascar and have chosen to include this sensitivity analysis to assess the generalizability of our results.

*Methods (line 504):*

*Because it benefits from on-going health system strengthening initiative, Ifanadiana’s baseline malaria testing and treatment rates may not be representative of national trends. Indeed, in the neighboring district of Farafangana, malaria treatment rates range between 19.6 – 23.4% [1], compared to rates of 33.2 – 56.6% in Ifanadiana. We therefore conducted a sensitivity analysis to explore the impact of ACD interventions at a lower baseline treatment rate, while keeping all other parameters as estimated. Following [1], we used a 20% baseline treatment rate for both children and adults and explored both low and high coverage rates of 40% and 80% respectively. For targeted interventions, ACD coverage responded to 80% and 100% for low and high coverage, respectively. We then simulated these interventions for all targeting strategies and both coverage rates and compared the average number of annually averted cases across 19 iterations.*

*Results (line 626):*

*The sensitivity analysis exploring the impact of an ACD intervention when baseline treatment rates were lower found similar results as the main analysis. ACD averted between 0.803-1.42% of simple cases, with little difference across low and high coverage. The number of severe cases averted was higher with higher coverage, ranging between 46.8-48.5% at low coverage and 59.6-61.4% at high levels (Supp. Table 3).*

**- Could one explore variation in the prophylactic protective period? Since it is now a fixed parameter, one could try for interventions with drugs with longer lasting half lives. Again I will leave it up to authors and no problems if it is left for a future study**

We agree this could be a useful additional analysis and find it particularly interesting among the sensitivity analyses proposed. Given it will expand the number interventions used to four and is not an intervention currently being considered by our Ministry partners, we have opted to leave this for a future study, but do mention it in the discussion as a line of future research (line 846).

**Data Collection:**

**- Could the authors include the scale of change to the data introduced by their ZERO-G adjustment? The reader would benefit from explicitly stating the magnitude of the difference between the raw and adjusted values. The other reviewer requested this (now in Figure S2 it appears) but it could be mentioned in the main text**

We have added this requested information (line 355):

*The ZERO-G adjustment was unique to each month and fokontany and resulted in case rates on average 5.72 times higher than the raw case rates (IQR: 2 – 6.6), dependent on the level of health care access (Supp. Fig. 2).*

**Author contributions:**

**- Thank you to to the authors for adding further detail for the author contributions. I still have some concerns so request the authors discuss with the editor to ensure compliance with policy on authorship**

*We will discuss further with the editor regarding authorship, thank you for your understanding.*
