# Supplementary material for: Leveraging spatial structure to design spatially-targeted malaria interventions at the community-scale
Source: PLoS Comput Biol. 2026 Oct 15;22(10):e1014857. doi: 10.1371/journal.pcbi.1014857 (PMC13690273; doi:10.1371/journal.pcbi.1014857)
Supplement: Peer review history

# Peer review: Decision Letter 0

**DOI:** 10.1371/journal.pcbi.1014857.r001

**Authors:** Roger Dimitri Kouyos, Chadi M. Saad-Roy

8 Apr 2026

PCOMPBIOL-D-26-00387

Leveraging spatial structure to design spatially-targeted malaria interventions at the community-scale

PLOS Computational Biology

Dear Dr. Evans,

Thank you for submitting your manuscript to PLOS Computational Biology. After careful consideration, we feel that it has merit but does not fully meet PLOS Computational Biology's publication criteria as it currently stands. Therefore, we invite you to submit a revised version of the manuscript that addresses the points raised during the review process.

Please submit your revised manuscript by Jun 08 2026 11:59PM. If you will need more time than this to complete your revisions, please reply to this message or contact the journal office at ploscompbiol@plos.org. When you're ready to submit your revision, log on to https://www.editorialmanager.com/pcompbiol/ and select the 'Submissions Needing Revision' folder to locate your manuscript file.

Please include the following items when submitting your revised manuscript:

* A letter that responds to each point raised by the editor and reviewer(s). You should upload this letter as a separate file labeled 'Response to Reviewers'. This file does not need to include responses to formatting updates and technical items listed in the 'Journal Requirements' section below.

* A marked-up copy of your manuscript that highlights changes made to the original version. You should upload this as a separate file labeled 'Revised Manuscript with Track Changes'.

* An unmarked version of your revised paper without tracked changes. You should upload this as a separate file labeled 'Manuscript'.

If you would like to make changes to your financial disclosure, competing interests statement, or data availability statement, please make these updates within the submission form at the time of resubmission. Guidelines for resubmitting your figure files are available below the reviewer comments at the end of this letter

We look forward to receiving your revised manuscript.

Kind regards,

Chadi M. Saad-Roy

Academic Editor

PLOS Computational Biology

Roger Kouyos

Section Editor

PLOS Computational Biology

**Journal Requirements:**

1) Please ensure that the CRediT author contributions listed for every co-author are completed accurately and in full.

At this stage, the following Authors/Authors require contributions: Michelle V. Evans, Benjamin Roche, Vincent Herbreteau, Christophe Revillion, Thibault Catry, Matthew H Bonds, Karen E Finnegan, Ezra Mitsinjoniala, Felana Ihantamalala, Mauricianot Randriamihaja, Omega Raobela, and Andres Garchitorena. Please ensure that the full contributions of each author are acknowledged in the "Add/Edit/Remove Authors" section of our submission form.

The list of CRediT author contributions may be found here: https://journals.plos.org/ploscompbiol/s/authorship#loc-author-contributions

2) We ask that a manuscript source file is provided at Revision. Please upload your manuscript file as a .doc, .docx, .rtf or .tex. If you are providing a .tex file, please upload it under the item type u2018LaTeX Source Fileu2019 and leave your .pdf version as the item type u2018Manuscriptu2019.

3) Please upload all main figures as separate Figure files in .tif or .eps format. For more information about how to convert and format your figure files please see our guidelines:

https://journals.plos.org/ploscompbiol/s/figures

4) We have noticed that you have uploaded Supporting Information files, but you have not included a list of legends. Please add a full list of legends for your Supporting Information files after the references list.

5) Please amend your detailed Financial Disclosure statement. This is published with the article. It must therefore be completed in full sentences and contain the exact wording you wish to be published.

1) State the initials, alongside each funding source, of each author to receive each grant. For example: "This work was supported by the National Institutes of Health (####### to AM; ###### to CJ) and the National Science Foundation (###### to AM)."

6) Some material included in your submission may be copyrighted. According to PLOS’s copyright policy, authors who use figures or other material (e.g., graphics, clipart, maps) from another author or copyright holder must demonstrate or obtain permission to publish this material under the Creative Commons Attribution 4.0 International (CC BY 4.0) License used by PLOS journals. Please closely review the details of PLOS’s copyright requirements here: PLOS Licenses and Copyright. If you need to request permissions from a copyright holder, you may use PLOS's Copyright Content Permission form.

Please respond directly to this email and provide any known details concerning your material's license terms and permissions required for reuse, even if you have not yet obtained copyright permissions or are unsure of your material's copyright compatibility. Once you have responded and addressed all other outstanding technical requirements, you may resubmit your manuscript within Editorial Manager.

Potential Copyright Issues:

i) Figures 1C, 1D, 2, and S1. please (a) provide a direct link to the base layer of the map (i.e., the country or region border shape) and ensure this is also included in the figure legend; and (b) provide a link to the terms of use / license information for the base layer image or shapefile. We cannot publish proprietary or copyrighted maps (e.g. Google Maps, Mapquest) and the terms of use for your map base layer must be compatible with our CC BY 4.0 license.

Note: if you created the map in a software program like R or ArcGIS, please locate and indicate the source of the basemap shapefile onto which data has been plotted.

If your map was obtained from a copyrighted source please amend the figure so that the base map used is from an openly available source. Alternatively, please provide explicit written permission from the copyright holder granting you the right to publish the material under our CC BY 4.0 license.

If you are unsure whether you can use a map or not, please do reach out and we will be able to help you. The following websites are good examples of where you can source open access or public domain maps:

* U.S. Geological Survey (USGS) - All maps are in the public domain. (http://www.usgs.gov)

* PlaniGlobe - All maps are published under a Creative Commons license so please cite “PlaniGlobe, http://www.planiglobe.com, CC BY 2.0” in the image credit after the caption. (http://www.planiglobe.com/?lang=enl)

* Natural Earth - All maps are public domain. (http://www.naturalearthdata.com/about/terms-of-use/)".

ii) Figure [5] contains screenshots. We are not permitted to publish these under our CC BY 4.0 license; websites are usually intellectual property and are copyrighted.This includes peripheral graphics of the web browser such as the [X] buttons. We ask that you please remove or replace it. .

Note: If the reviewer comments include a recommendation to cite specific previously published works, please review and evaluate these publications to determine whether they are relevant and should be cited. There is no requirement to cite these works unless the editor has indicated otherwise.

**Reviewers' comments:**

Reviewer's Responses to Questions

**Comments to the Authors:**

**Please note that one review is uploaded as an attachment.**

Reviewer #1: Review of: Leveraging spatial structure to design spatially-targeted malaria interventions at the community-scale by Evans et al

Manuscript Number: PCOMPBIOL-D-26-00387

Journal: PLOS Computational Biology

2026 March 06

Summary:

Evans et al present a model-based exploration of targeting malaria interventions, parameterizing their model with data from a district in Madagascar. Three intervention types are investigated for a suite of spatial targeting strategies, and are compared to a non-targeted scenario. Such work is timely, as stalled progress in controlling malaria and funding shortages are necessitating conversation about new approaches to the deployment of interventions. The reviewer commends the authors on a well-written manuscript and thorough development of a model incorporating much of the complexity of the system. If the authors are able to address a few major and some minor comments, it is the opinion of this reviewer that this work could be an important contribution to our understanding of malaria epidemiology in the present moment. See below for the comments organized by section, with major comments focusing on (i) the mobility analyses, where improvements are needed to clarify for the reader and aid in reproducibility, (ii) potential circularity/bias in estimating the effectiveness of bednet distributions, and (iii) accounting for immunity (or the lack thereof) in malaria transmission models

Major comments:

(1) Mobility - This section of the study would benefit from greater clarity.

First, the methods section on mobility (starting at line 261) was difficult to follow. Are data on observed rates of movement by human hosts in this district included in the study? As it currently reads I think movement is assumed, and just straight line distances or distances along paths is considered but could the authors clarify? If just using distance, "distance dataset" is more appropriate I believe than "mobility data" (e.g., Lines 270, 292 etc), which implies some measurement of actual movement. Likewise "daily commuting" was confusing to this reviewer, and may be so to a general audience. I wouldn't expect much daily travel in a rural, subsistence agriculture area and 'daily' commuting implies people are sleeping at their residence (and given malaria is transmitted at night, daytime commuting would not be relevant to transmission).

Additionally, could the authors report and assess the scale of travel they are seeing in their models? In other words, is the amount of travel needed to get the increased performance as seen in the summary statistics realistic? Is it consistent with data on actual observations of overnight intra-district travel? Or is inter-patch travel as included in the model capturing some other source of variation in malaria infection risk across the district (e.g., travel times correlate with bednet deployment success historically or something along those lines)? I ask because I struggle to see the biological mechanism underpinning the explanatory power of a distance-matrix here - would this not require a significant proportion of hosts to be spending nights in patches with different infection probabilities?

(2) Bednets as an intervention of focus

The observed case rate and malaria prevalence data are presumably measuring malaria infections that occurred on a background of variable but high bednet ownership and usage in the district historically. It is not clear how one can cleanly account for this. For example, it seems necessary to parse out the contribution of 'bednet-preventable cases' (the cases from those without a bednet but that would use a bednet if more were deployed) vs 'residual cases' (cases from those already with access to a bednet but that bednets alone failed to prevent, due to many other factors beyond outdoor biting) to total infection burden. I suspect that, unfortunately, much of the malaria burden that can be averted by bednets is already being averted and is therefore baked into the estimates of infection and biting rates that inform FOI in the model. One would need a 'clean' estimate of what FOI and biting rates would be if 'untainted' by existing bednet usage in the area to fully explore the benefits of bednets. The IRS and ACD interventions avoid this concern as they have not yet been implemented in the district, so I suggest focusing further on those

From an epidemiological perspective - universal coverage by bednets from mass distribution campaigns has been the core of malaria control for a while. The scenarios explored here seem to be a retreat from that. Rather than a new "targeted" approach, the authors seem to suggest restricting bednet distribution below the current coverage, for the first time foregoing areas that would typically be included in distribution efforts. There seem to be both ethical considerations (removing public health goods from communities that have become reliant on them) and a broader problem: if the malaria control program is no longer able to even try to distribute bednets across districts where it normally does so this would undoubtedly be associated with a broader system collapse and a fundamentally different malaria control regime than was in place over the time series considered in this analysis

Finally, 'subnational tailoring' of bednet distributions has been explored before. For example see work from Thomas Churcher's group in eLife (https://elifesciences.org/reviewed-preprints/108745), where they use a statistical approach to estimate the impact of changing bednet distribution strategy, avoiding some of the pitfalls outlined above and below. There are probably limitations to that approach as well, but it speaks to the general difficulty of focusing the present study on bednets given the approach and data available

(3) Accounting for biological realities/complexities of malaria transmission

As the authors note in the discussion, a full accounting of the complex transmission biology of malaria is impossible. But a few items are worth consideration. Mainly, as is well known, using SEIR type models for malaria is difficult due to concurrent infections (ie the infected class is susceptible to being multiply infected). A high proportion of falciparum infections contain multiple parasite genotypes indicating superinfection (and this has been shown to be the case in Madagascar too https://pubmed.ncbi.nlm.nih.gov/34044837/ https://pubmed.ncbi.nlm.nih.gov/27998292/). As a result, tying gamma_A to the patent period violates our understanding of malaria transmission.

My guess is collapsing all malaria multiplicities of infection to dichotomous malaria present/absent would result in substantially underestimating FOI with downstream consequences for estimating the effect of interventions. In the literature, FOI for malaria is thus usually estimated from laborious genotyping methods (see Ivo Mueller's mFOI work from New Guinea https://pubmed.ncbi.nlm.nih.gov/22665809/) or from cohorts with e.g., follow-up of infections cleared by treatment. My intuition is that ignoring multiple infections blunts the effectiveness of spatial targeting: Individuals with a high risk of infection get repeatedly infected, but these additional infection events are uncounted as they are unobserved during the patent period. As a result FOI is even higher than estimated in high FOI areas, whereas this undercounting is less strong in low FOI areas where the probability of multiple infection is lower. One could consider various approaches to account for this, but it seems to be a limitation for the current manuscript that warrants attention.

Minor comments by section:

Abstract and author summary:

- The third sentence of the abstract reads as a long list of limitations for targeted interventions. This may not be necessary here as not all limitations listed are relevant to the context considered in the manuscript (e.g., non-endemic settings) and it takes away space from building the narrative

- By definition a complete ACD program would catch all cases?

- Could the authors define the equally distributed scenario? Is this un-targeted? If so the reader may benefit from further clarity here (i.e., stating seomthing along the lines of 'We compared X scenarios, one with no targeting and then others with targeting by XYZ...'). Later in the manuscript (e.g., line 603) this is confusing as well. Is "equally distributed targeting" the absence of targeting? Perhaps "non-targeted" is clearer

Introduction:

- Refs 6 and 7 don't support the sentence as written. The Carlson et al 2023 piece is a short opinion piece without any data and the Filho et al Malaria Journal article only shows some coarse trends in national incidence stats and mean annual temp for a handful of countries

- The discussion of CHWs is rather aspirational. While on paper CHWs are charged with the listed tasks, it is unclear if it actually often happens on the ground outside of a single, well supported district in Madagascar. The authors point to some success stories where CHWs were used for TB, maternal health, and pandemic response but the discussion in the intro would benefit from balance to better represent the state of the field. I suggest authors mention the numerous counter examples where a lack of funding for CHWs, infrastructure, stockouts of consummables etc result in major challenges. For example, previous work by the author group identified challenges even within controlled study supported areas (https://pmc.ncbi.nlm.nih.gov/articles/PMC12874809/). In the Madagascar context, stock-outs have received attention (e.g., see https://pmc.ncbi.nlm.nih.gov/articles/PMC6144492/). In other locales, similar challenges have been reported (e.g., https://link.springer.com/article/10.1186/s12889-018-6040-2, https://link.springer.com/article/10.1186/s12913-020-05458-1, https://pmc.ncbi.nlm.nih.gov/articles/PMC4155545/). While I agree CHWs have potential in this role, the intro should be framed accordingly, making it clear this is an aspiration rather than a current capacity for CHWs across Madagascar and similar settings.

Methods:

There are limitations of using the selected district (Ifanadiana) to guide general policy as it is likely not representative of the 113+ other districts in Madagascar. For example, the two decades of (commendable) health system strengthening activities likely make the passive case detection and treatment rates different in the district than in other districts (with an effect on parameter tau_s*). Base treatment rates are quite high in the district (30-55%) [Lines 397-399]. In a nearby district, Mananjary, we saw treatment rates were much lower (less than 5%) (https://www.science.org/doi/10.1126/science.adp5365)

- Is waning for symptomatic cases (gamma_P) tied to the known half-lives / clearance rates of the drugs used? I wasn't sure why this parameter is estimated from the data (if I am reading Table S1 correctly), when the time duration of clinical efficacy of the drugs is known

- Others have explored adding a longer lasting antimalarial (such as SP or DP) to ACD. The framework here seems well suited to exploring the potential benefits of interventions that use drugs with different durations of protection and is perhaps worth exploring

- The "m" parameter does not appear to be defined in the initial model definition section and as this is often used as the number of mosquitoes in malaria models it created momentary confusion, for me at least, until defined later as the mobility matrix. Could the authors flag this in the first model description (i.e., Eqns 7 and 12)?

- Important to note that the Mordecai 2013 thermal response curves should be used with caution. It remains unclear how data from lab reared mosquitoes held at constant temperatures translate to the real world where mosquitoes can navigate complex landscapes to buffer against high temperatures (e.g., they can hide in the shade or in wells on hot days). Additionally, the Mordecai 2013 study used mosquito species that are not present in Madagascar (e.g., biting rate as a function of time is inferred for Anopheles pseudopunctipennis, a species only found in the Americas) or used lineages of An. gambiae from Lagos, Nigeria that are unlikely to represent those in Madagascar

Data Collection:

- The (large) scale of under-ascertainment and thus the scale of adjustment needed for case rates should be mentioned. Per the authors' 2023 Sci Rep paper, "case notifications are capturing on average 26.5% of symptomatic malaria cases in the district". Given this level of under-ascertainment, there is likely considerable uncertainty in true case rates even after corrections are applied and the reader should be made aware of this limitation - especially as these 'corrected' case rates are referred to as 'observed' cases later in the manuscript it seems

- For clarity: "Targeted" intervention brings up connotations of "supplemental" intervention (e.g., chemoprophylaxis, vaccination or something new and fancy) that is layered on top of broad coverage of base/routine interventions (e.g., bednets) for a specific targeted population (e.g., individuals with an occupation that predicts higher exposure risk). Targeted seems to be used in a different, and perhaps unexpected way here, where routine interventions (e.g., bednets, spraying) are restricted in some scenarios. I'm not sure how this would work in practice - future mass bednet distribution campaigns which would have by default aimed at 100% coverage, instead forego distribution in some areas I guess? The manuscript would benefit from clarity here and given the point above about nets confounding the parameterization of the model it is likely more useful to focus on other intervention types

Author contributions:

- I have a few concerns re authorship as currently listed. PLOS Computational Biology states the requirements for authorship include: "Substantial contributions to one or more of the following: conception or design of the work; acquisition, analysis, or interpretation of data; creation of new software used in the work; drafting or substantially revising the article" (https://journals.plos.org/ploscompbiol/s/authorship). Currently, authors KEF and OR are listed as only providing "project supervision" and author EM does not appear to be mentioned in the author contributions section. I understand it is sometimes convention to add senior organization leadership as authors regardless of their scientific contribution. I empathize with the pressure to do so - and the politcal complexities - but encourage the authors to consider carefully - not to simply tweak the author contribution statement. I also encourage the editor to clarify as needed

Reviewer #2: Please see attached file.

**********

**Have the authors made all data and (if applicable) computational code underlying the findings in their manuscript fully available?**

The PLOS Data policy requires authors to make all data and code underlying the findings described in their manuscript fully available without restriction, with rare exception (please refer to the Data Availability Statement in the manuscript PDF file). The data and code should be provided as part of the manuscript or its supporting information, or deposited to a public repository. For example, in addition to summary statistics, the data points behind means, medians and variance measures should be available. If there are restrictions on publicly sharing data or code —e.g. participant privacy or use of data from a third party—those must be specified.

Reviewer #1: Yes

Reviewer #2: Yes

**********

PLOS authors have the option to publish the peer review history of their article (what does this mean?). If published, this will include your full peer review and any attached files.

If you choose “no”, your identity will remain anonymous but your review may still be made public.

**Do you want your identity to be public for this peer review?**  For information about this choice, including consent withdrawal, please see our Privacy Policy.

Reviewer #1: **Yes:**  Benjamin Rice

Reviewer #2: No

[NOTE: If reviewer comments were submitted as an attachment file, they will be attached to this email and accessible via the submission site. Please log into your account, locate the manuscript record, and check for the action link "View Attachments". If this link does not appear, there are no attachment files.]

**Figure resubmission:**

While revising your submission, we strongly recommend that you use PLOS’s NAAS tool (https://ngplosjournals.pagemajik.ai/artanalysis) to test your figure files. NAAS can convert your figure files to the TIFF file type and meet basic requirements (such as print size, resolution), or provide you with a report on issues that do not meet our requirements and that NAAS cannot fix.

After uploading your figures to PLOS’s NAAS tool - https://ngplosjournals.pagemajik.ai/artanalysis, NAAS will process the files provided and display the results in the "Uploaded Files" section of the page as the processing is complete. If the uploaded figures meet our requirements (or NAAS is able to fix the files to meet our requirements), the figure will be marked as "fixed" above. If NAAS is unable to fix the files, a red "failed" label will appear above. When NAAS has confirmed that the figure files meet our requirements, please download the file via the download option, and include these NAAS processed figure files when submitting your revised manuscript.

**Reproducibility:**

To enhance the reproducibility of your results, we recommend that authors of applicable studies deposit laboratory protocols in protocols.io, where a protocol can be assigned its own identifier (DOI) such that it can be cited independently in the future. Additionally, PLOS ONE offers an option to publish peer-reviewed clinical study protocols. Read more information on sharing protocols at https://plos.org/protocols?utm_medium=editorial-email&utm_source=authorletters&utm_campaign=protocols

Attachment Submitted filename: Review of PLOS CB spatial malaria.docx

# Author comment: Author response to Decision Letter 1

**DOI:** 10.1371/journal.pcbi.1014857.r002

9 Jul 2026

Attachment Submitted filename: spatial-malaria-response.pdf

# Peer review: Decision Letter 1

**DOI:** 10.1371/journal.pcbi.1014857.r003

**Authors:** Roger Dimitri Kouyos, Chadi M. Saad-Roy

17 Aug 2026

PCOMPBIOL-D-26-00387R1

Leveraging spatial structure to design spatially-targeted malaria interventions at the community-scale

PLOS Computational Biology

Dear Dr. Evans,

Thank you for submitting your manuscript to PLOS Computational Biology. After careful consideration, we feel that it has merit but does not fully meet PLOS Computational Biology's publication criteria as it currently stands. Therefore, we invite you to submit a revised version of the manuscript that addresses the points raised during the review process.

Please submit your revised manuscript by Oct 17 2026 11:59PM. If you will need more time than this to complete your revisions, please reply to this message or contact the journal office at ploscompbiol@plos.org. When you're ready to submit your revision, log on to https://www.editorialmanager.com/pcompbiol/ and select the 'Submissions Needing Revision' folder to locate your manuscript file.

Please include the following items when submitting your revised manuscript:

* A letter that responds to each point raised by the editor and reviewer(s). You should upload this letter as a separate file labeled 'Response to Reviewers'. This file does not need to include responses to formatting updates and technical items listed in the 'Journal Requirements' section below.

* A marked-up copy of your manuscript that highlights changes made to the original version. You should upload this as a separate file labeled 'Revised Manuscript with Track Changes'.

* An unmarked version of your revised paper without tracked changes. You should upload this as a separate file labeled 'Manuscript'.

If you would like to make changes to your financial disclosure, competing interests statement, or data availability statement, please make these updates within the submission form at the time of resubmission. Guidelines for resubmitting your figure files are available below the reviewer comments at the end of this letter.

As the corresponding author, your ORCID iD is verified in the submission system and will appear in the published article. PLOS supports the use of ORCID, and we encourage all coauthors to register for an ORCID iD and use it as well. Please encourage your coauthors to verify their ORCID iD within the submission system before final acceptance, as unverified ORCID iDs will not appear in the published article. Only the individual author can complete the verification step; PLOS staff cannot verify ORCID iDs on behalf of authors.

We look forward to receiving your revised manuscript.

Kind regards,

Chadi M. Saad-Roy

Academic Editor

PLOS Computational Biology

Roger Kouyos

Section Editor

PLOS Computational Biology

**Additional Editor Comments (if provided):**

**Journal Requirements:**

If the reviewer comments include a recommendation to cite specific previously published works, please review and evaluate these publications to determine whether they are relevant and should be cited. There is no requirement to cite these works unless the editor has indicated otherwise.

1) Please ensure that the CRediT author contributions listed for every co-author are completed accurately and in full.

At this stage, the following Authors/Authors require contributions: Michelle V. Evans, Benjamin Roche, Vincent Herbreteau, Christophe Revillion, Thibault Catry, Matthew H Bonds, Karen E Finnegan, Ezra Mitsinjoniala, Felana Ihantamalala, Mauricianot Randriamihaja, Omega Raobela, and Andres Garchitorena. Please ensure that the full contributions of each author are acknowledged in the "Add/Edit/Remove Authors" section of our submission form.

The list of CRediT author contributions may be found here: https://journals.plos.org/ploscompbiol/s/authorship#loc-author-contributions

2) Please upload a copy of Figure Fig 1-5 which you refer to in your text on pages 5, and 17. Or, if the figure is no longer to be included as part of the submission please remove all reference to it within the text.

3) Please amend your detailed Financial Disclosure statement. This is published with the article. It must therefore be completed in full sentences and contain the exact wording you wish to be published.

i) State what role the funders took in the study. If the funders had no role in your study, please state: "The funders had no role in study design, data collection and analysis, decision to publish, or preparation of the manuscript."

ii) If any authors received a salary from any of your funders, please state which authors and which funders..

If you did not receive any funding for this study, please simply state: u201cThe authors received no specific funding for this work.u201d

**Reviewers' comments:**

Reviewer's Responses to Questions

**Comments to the Authors:**

**Please note here if the review is uploaded as an attachment.**

Reviewer #1: Please see attached document

Reviewer #2: I thank the authors for a detailed and thoughtful response to all comments. I found the paper substantially improved in the revision, and I appreciated the attention to incorporating new sensitivity analyses.

One minor note — the authors included a supplemental figure in the response on mosquito mortality as a function of water, and I agree with them that this figure need not be included in the manuscript’s supplemental.

**********

**Have the authors made all data and (if applicable) computational code underlying the findings in their manuscript fully available?**

The PLOS Data policy requires authors to make all data and code underlying the findings described in their manuscript fully available without restriction, with rare exception (please refer to the Data Availability Statement in the manuscript PDF file). The data and code should be provided as part of the manuscript or its supporting information, or deposited to a public repository. For example, in addition to summary statistics, the data points behind means, medians and variance measures should be available. If there are restrictions on publicly sharing data or code —e.g. participant privacy or use of data from a third party—those must be specified.

Reviewer #1: Yes

Reviewer #2: None

**********

PLOS authors have the option to publish the peer review history of their article (what does this mean?). If published, this will include your full peer review and any attached files.

If you choose “no”, your identity will remain anonymous but your review may still be made public.

**Do you want your identity to be public for this peer review?**  For information about this choice, including consent withdrawal, please see our Privacy Policy.

Reviewer #1: **Yes:**  Benjamin L Rice

Reviewer #2: **Yes:**  Chirag K Kumar

[NOTE: If reviewer comments were submitted as an attachment file, they will be attached to this email and accessible via the submission site. Please log into your account, locate the manuscript record, and check for the action link "View Attachments". If this link does not appear, there are no attachment files.]

**Figure resubmission:**

While revising your submission, we strongly recommend that you use PLOS’s NAAS tool (https://ngplosjournals.pagemajik.ai/artanalysis) to test your figure files. NAAS can convert your figure files to the TIFF file type and meet basic requirements (such as print size, resolution), or provide you with a report on issues that do not meet our requirements and that NAAS cannot fix.

After uploading your figures to PLOS’s NAAS tool - https://ngplosjournals.pagemajik.ai/artanalysis, NAAS will process the files provided and display the results in the "Uploaded Files" section of the page as the processing is complete. If the uploaded figures meet our requirements (or NAAS is able to fix the files to meet our requirements), the figure will be marked as "fixed" above. If NAAS is unable to fix the files, a red "failed" label will appear above. When NAAS has confirmed that the figure files meet our requirements, please download the file via the download option, and include these NAAS processed figure files when submitting your revised manuscript.

**Reproducibility:**

To enhance the reproducibility of your results, we recommend that authors of applicable studies deposit laboratory protocols in protocols.io, where a protocol can be assigned its own identifier (DOI) such that it can be cited independently in the future. Additionally, PLOS ONE offers an option to publish peer-reviewed clinical study protocols. Read more information on sharing protocols at https://plos.org/protocols?utm_medium=editorial-email&utm_source=authorletters&utm_campaign=protocols

# Author comment: Author response to Decision Letter 2

**DOI:** 10.1371/journal.pcbi.1014857.r004

10 Sep 2026

Attachment Submitted filename: spatial-malaria-intervention-response2.docx

# Peer review: Decision Letter 2

**DOI:** 10.1371/journal.pcbi.1014857.r005

**Authors:** Roger Dimitri Kouyos, Chadi M. Saad-Roy

25 Sep 2026

Dear Dr. Evans,

We are pleased to inform you that your manuscript 'Leveraging spatial structure to design spatially-targeted malaria interventions at the community-scale' has been provisionally accepted for publication in PLOS Computational Biology.

Before your manuscript can be formally accepted you will need to complete some formatting changes, which you will receive in a follow up email. A member of our team will be in touch with a set of requests.

Please note that your manuscript will not be scheduled for publication until you have made the required changes, so a swift response is appreciated.

IMPORTANT: The editorial review process is now complete. PLOS will only permit corrections to spelling, formatting or significant scientific errors from this point onwards. Requests for major changes, or any which affect the scientific understanding of your work, will cause delays to the publication date of your manuscript.

Should you, your institution's press office or the journal office choose to press release your paper, you will automatically be opted out of early publication. We ask that you notify us now if you or your institution is planning to press release the article. All press must be co-ordinated with PLOS.

Thank you again for supporting Open Access publishing; we are looking forward to publishing your work in PLOS Computational Biology.

Best regards,

Chadi M. Saad-Roy

Academic Editor

PLOS Computational Biology

Roger Kouyos

Section Editor

PLOS Computational Biology

***********************************************************

Reviewer's Responses to Questions

**Comments to the Authors:**

**Please note here if the review is uploaded as an attachment.**

Reviewer #1: Reviewer comments uploaded as attachment

**********

**Have the authors made all data and (if applicable) computational code underlying the findings in their manuscript fully available?**

The PLOS Data policy requires authors to make all data and code underlying the findings described in their manuscript fully available without restriction, with rare exception (please refer to the Data Availability Statement in the manuscript PDF file). The data and code should be provided as part of the manuscript or its supporting information, or deposited to a public repository. For example, in addition to summary statistics, the data points behind means, medians and variance measures should be available. If there are restrictions on publicly sharing data or code —e.g. participant privacy or use of data from a third party—those must be specified.

Reviewer #1: Yes

**********

PLOS authors have the option to publish the peer review history of their article (what does this mean?). If published, this will include your full peer review and any attached files.

If you choose “no”, your identity will remain anonymous but your review may still be made public.

**Do you want your identity to be public for this peer review?**  For information about this choice, including consent withdrawal, please see our Privacy Policy.

Reviewer #1: **Yes:**  Benjamin L Rice

# Editor report: Acceptance letter

**DOI:** 10.1371/journal.pcbi.1014857.r006

**Authors:** Roger Dimitri Kouyos, Chadi M. Saad-Roy

PCOMPBIOL-D-26-00387R2

Leveraging spatial structure to design spatially-targeted malaria interventions at the community-scale

Dear Dr Evans,

I am pleased to inform you that your manuscript has been formally accepted for publication in PLOS Computational Biology. Your manuscript is now with our production department and you will be notified of the publication date in due course.

The corresponding author will soon be receiving a typeset proof for review, to ensure errors have not been introduced during production. Please review the PDF proof of your manuscript carefully, as this is the last chance to correct any errors. Please note that major changes, or those which affect the scientific understanding of the work, will likely cause delays to the publication date of your manuscript.

Soon after your final files are uploaded, unless you have opted out, the early version of your manuscript will be published online. The date of the early version will be your article's publication date. The final article will be published to the same URL, and all versions of the paper will be accessible to readers.

For Research, Software, and Methods articles, you will receive an invoice from PLOS for your publication fee after your manuscript has reached the completed accept phase. If you receive an email requesting payment before acceptance or for any other service, this may be a phishing scheme. Learn how to identify phishing emails and protect your accounts at https://explore.plos.org/phishing.

Thank you again for supporting PLOS Computational Biology and open-access publishing. We are looking forward to publishing your work!

With kind regards,

Janani Seenivasan

PLOS Computational Biology | Carlyle House, Carlyle Road, Cambridge CB4 3DN | United Kingdom ploscompbiol@plos.org | Phone +44 (0) 1223-442824 | ploscompbiol.org | @PLOSCompBiol
